# Lathyrol inhibits the proliferation of Renca cells by altering expression of TGF-β/Smad pathway components and subsequently affecting the cell cycle

**DOI:** 10.3389/fonc.2025.1629962

**Published:** 2025-10-08

**Authors:** Shengyou Song, Yalin Song

**Affiliations:** 1 Department of Education, Shandong Provincial Hospital, Jinan, China; 2 Department of Urology, Zaozhuang Municipal Hospital, Zaozhuang, China

**Keywords:** lathyrol, renal cell cancer, TGF-β/Smad signalingsmad signal pathway, cell cycle, proliferation

## Abstract

**Background:**

Renal cell carcinoma (RCC) is a prevalent type of malignant tumor with high morbidity and mortality. The TGF-β/Smad signaling pathway plays a significant role in the development and progression of RCC.

**Methods:**

This study explored the effects of lathyrol on the proliferation of Renca mouse RCC cells through the inhibition of the TGF-β/Smad signaling pathway and cell cycle arrest. Bioinformatics analysis, cell culture experiments, and animal experiments were conducted to determine the effects of lathyrol on the activity, mRNA expression, and protein expression of RCC cells and RCC xenograft tumors, as well as the expression of cell cycle and cell cycle regulatory proteins.

**Results:**

Lathyrol treatment was positively correlated with the inhibitory effect on cell proliferation. The IC values of 786-O cells and Renca cells were comparable. *In vitro*, lathyrol promoted both protein and mRNA expression of TGF-β1 while increasing Smad6 protein expression and Smad2, Smad3, and Smad4 mRNA expression; concurrently, it suppressed the protein expression of Smad2, Smad3, Smad4, and Smad9. *In vivo*, lathyrol suppressed the mRNA and protein expression of TGF-β1, TGF-βR1, Smad2, Smad3, Smad4, and Smad9 in RCC xenografts; promoted the protein expression of Smad6; and decreased the protein expression of cyclin D1, cyclin B1, cyclin A1, cyclin E1, CDK6, CDK4, and CDK1 while increasing the expression of P16, P21, and P27.

**Conclusion:**

Lathyrol can repress the expression of key proteins in the TGF-β/Smad signaling pathway, impede signal transduction, arrest the cell cycle progression of Renca cells, and subsequently inhibit the proliferation of RCC cells. Future studies are needed to further explore the mechanism of lathyrol in RCC treatment.

## 1 Introduction

Renal cell carcinoma (RCC) constitutes roughly 2% to 3% of adult malignant neoplasms- with a global male: female ratio of approximately 1.5:1. This disease can emerge in all age brackets, with the highest incidence occurring between 60 and 70 years of age and a median age of approximately 64 years (1). Currently, the incidence of RCC increasing annually, rendering it the second most prevalent urinary system neoplasm. Despite its lower incidence than prostate cancer (PCa), among patients with malignant tumors affecting the urinary system, the prognosis of RCC is poorer than that of other urinary system tumors (2). Recent advances in renal cell carcinoma (RCC) treatment highlight combination therapies, particularly immune checkpoint inhibitors (ICIs) with vascular endothelial growth factor tyrosine kinase inhibitors (VEGF TKIs), which are now established as the standard first-line approach for clear-cell RCC and significantly improve overall survival rates (3, 4). However, ongoing studies continue to elucidate the mechanisms of immunotherapy resistance in RCC, revealing novel epigenetic therapeutic targets and opening new avenues for next-generation immune-targeted therapies (5).

The TGF-β/Smad signaling pathway is a multifunctional cytokine signaling pathway that plays a vital role in the genesis and development of neoplasms and facilitates the advancement of neoplastic cells via diverse mechanisms. On the one hand, the activation of the TGF-β/Smad signaling pathway in cancer and related disorders can stimulate the proliferation of carcinomas and increase the proliferation and dissemination of carcinoma cells, thereby expediting the progression of cancerous diseases (6) and leading to a decrease in the sensitivity of cancer cells to clinical drugs and thus reducing the effectiveness of anticancer drugs (7). It can also induce carcinoma cells to undergo epithelial–mesenchymal transition (EMT), among other effects (8)., such that the originally closely-connected epithelial cells transform into cells with mesenchymal characteristics, thereby attaining stronger invasion and migration capabilities. This transformation enables neoplasm cells to more readily detach from the primary tumor. It also allows them to enter the bloodstream and ultimately form metastases, significantly increasing the risk of prognosis deterioration in cancer patients (9). On the other hand, the TGF-β/Smad signaling pathway is closely associated with the cell cycle. Regulating the expression and signal transduction of the TGF-β/Smad signaling pathway can thereby influence the normal operation of the cell cycle of cancer cells, thereby affecting the proliferation, apoptosis and other phenotypes of carcinoma cells, and influencing the malignant behavior of neoplasm cells (10, 11). Consequently, the TGF-β/Smad signaling pathway plays crucial roles in the proliferation of carcinoma cells, regulation of cell cycle activity, and EMT, as well as other biological behaviors, and has emerged as an important therapeutic target in neoplasm treatment research. Modulation of this signaling pathway is anticipated to offer novel strategies for improving the prognosis of cancer patients.

Lathyrol (chemical formula: C20H30O4) is one of the active ingredients of the traditional Chinese medicine Semen Euphorbiae Lathyridis and can eliminate water, resolve blood stasis, and dissipate masses (12). It is mainly used in the clinical treatment of constipation, edema, phlegm retention, abdominal distension, blood stasis and amenorrhea, and can be applied externally to treat stubborn tinea and warts (13). Through a literature review and experimental studies, we found that lathyrol monomers can exhibit antitumor effects both *in vivo* and *in vitro* and can quench the malignant behavior of tumors (14, 15). Although lathyrol has significant antineuronal effects, its mechanism has not been fully explored; in particular, the mechanism underlying its inhibitory effect on cancer cell proliferation requires further in-depth study. Moreover, the pathogenesis of RCC requires further exploration. On this basis, we hypothesize that lathyrol might suppress the proliferation of RCC cells by inhibiting expression of TGF-β/Smad signaling pathway components in RCC, affecting the function of the RCC cell cycle and thus exerting an antitumor effect. To verify this hypothesis, we further investigated whether lathyrol could inhibit progression of the RCC cell cycle by suppressing the activity of the TGF-β/Smad signaling pathway, thereby inhibiting the proliferation of RCC cells, by culturing RCC cells and constructing a RCC mouse model.

## 2 Main materials and methods

### 2.1 Main materials

The following reagents and materials were used in this study: 786-O human RCC cell line (Procell Life Science & Technology Co., CL-0010) and Renca mice RCC cell line (Shanghai Zhong Qiao Xin Zhou Biotechnology Co., Ltd., ZQ0996) authenticated by RTS (Short Tandem Repeat) authentication and mycoplasma tests; lathyrol (Weikeqi-Biotech, Sichuan, China, Wakq-00424); carboplatin (Qilu Pharmaceutical Co., LTD. H10920028); BALB/c male SPF mice (SPF (Beijing) Biotechnology Co., Ltd., license: SCXK (Beijing) 2019–0010), 1.5-2–month-old and weighing 20 ± 2 g, which were reared in the Laboratory Animal Centre at 25°C in individually ventilated cages (IVCs) with food and water provided ad libitum; RPMI 1640 medium(Procell Biotechnology Co., Ltd.); fetal bovine serum (FBS, Shanghai Univ bio-Co., Ltd.); a CCK-8 kit and phosphate-buffered saline (PBS) buffer (Biosharp Co.); trypsin-ethylenediaminetetraacetic acid (EDTA) (0.25%) digestion solution, enhanced chemiluminescence (ECL) developer, cell culture grade dimethyl sulfoxide (DMSO), tissue and cell radioimmunoprecipitation assay (RIPA) lysis buffer, phenyl methyl sulfonyl fluoride (PMSF), goat blocking serum, hydrogen peroxide (H_2_O_2_), Mayer’s hematoxylin staining solution and neutral gum for immunohistochemistry (IHC) (Solarbio Co., Ltd.); a bicinchoninic acid (BCA) protein quantification kit and horseradish peroxidase (HRP)-labeled goat anti-rabbit secondary antibody (Beyotime Biotechnology Company) and a real-time polymerase chain reaction(RT-PCR) kit, bovine serum albumin (BSA), prestained protein marker (10-180KD), cell membrane breaking solution, and IHC staining reagent (Servicebio Co., Ltd). *In vivo* antibodies included the following: TGF-β1, TGF-βR1, Smad2 + 3, Smad6, and Smad9 antibodies (Servicebio Co., Ltd.); Smad4, CDK1, PCNA, and Ki67 (Wuhan ProteinTech Co.); and CDK2, CDK4, CDK6, P16, P21, P27, cyclin D1, cyclin A1, cyclin B1, and cyclin E1 (Shenzhen Youpin biotech-Co.). *In vitro* antibodies included the following: TGF-β1, TGF-βR1, PCNA, Ki67, Smad6, Smad9, CDK6, cyclin A1, cyclin B1, cyclin D1, and P16 (Servicebio Co., Ltd.) and Smad2, Smad3, and Smad4 (Wuhan ProteinTech Co.).

### 2.2 Methods

#### 2.2.1 General bioinformatics analysis

Using bioinformatics analysis, the differential expression and clinical prognosis of the TGF-β1, TGF-βR1, Smad1, Smad2, Smad3, Smad4, Smad6, and Smad9 proteins in the TGF-β/Smad signaling pathway in RCC patients were analyzed. Patient information was downloaded from the TCGA database (https://portal.gdc.cancer.gov), the RNAseq data of the TCGA-KIRC (renal clear cell carcinoma) project STAR process were analyzed, and the data in TPM format and clinical data were extracted. The data was filtered with the following parameters: remove normal + remove no clinical information. The data processing method was a follows: log2(value+1). Differences in expression were selected according to the data distribution as follows: T test (satisfying a normal distribution + a homogenous variance); Welch t test (satisfying a normal distribution + not satisfying a homogeneous variance); Wilcoxon rank sum test (not satisfying a normal distribution, nonparametric test); and Cox regression analysis (clinical prognosis analysis). The data were subsequently imported into the Xiantao Academic Tool (https://www.xiantao.love) for visualization and processing.

#### 2.2.2 Cell culture

The 786-O and Renca RCC cells were regularly grown in a constant-temperature cell incubator (with 37°C, 5% CO2 and 95% humidity) in cell culture medium [containing RPMI 1640 medium, 10% FBS and 1% penicillin and streptomycin (PS)]. When the cells reached approximately 85% confluence on the culture flask surface, the cells in the logarithmic phase of growth were digested and utilized in subsequent experiments.

#### 2.2.3 Establishment of a cell viability–drug concentration curve

A CCK-8 assay was used to determine the cell viability of 786-O and Renca RCC cells treated with different concentrations of lathyrol solution for 24 hours, and the RCC cell viability–drug concentration and regression curve formula were used to calculate the concentrations of specific drugs (IC10, IC25, IC75, and IC90), after which the differences in the effects of the drugs on the two types of RCC cells were compared.

#### 2.2.4 Cell grouping

On the basis of the results of preliminary experiments, the cells were divided into a DMSO control group (A group), an experimental group (B group) and a negative control group (C group), and the IC_50_ values were selected as the intervention concentrations of the B group and C group for 24h. Cells in the DMSO control group were grown in DMSO working medium (containing RPMI 1640 medium and 1% DMSO), those in the negative control group were cultured in paraplatin working medium (containing DMSO working medium and corresponding carboplatin drug concentrations), and those in the experimental group were cultured in lathyrol working medium (containing DMSO working medium and corresponding lathyrol drug concentrations).

#### 2.2.5 Construction of the RCC mouse model

Upon centrifugation for cell collection, the initial cell suspension was formulated in complete RPMI-1640 medium supplemented with 10% FBS, and adjusted to a concentration of 1.5 × 107 cells/ml. Depilatory cream was used to eliminate hair and expose the skin of the axilla (right forelimb). The Renca cell suspension (0.10 mL) was subsequently injected into the subcutaneous tissue of each mouse in the axilla (right forelimb), and the mice were housed in SPF-grade IVCs. The mice were permitted to consume food and water normally. The entire animal husbandry process and all the experimental procedures in this study adhered to the relevant experimental management requirements and ethical standards for experimental animal welfare. All the experimental protocols used in this study were approved by the Ethics Committee of Zaozhuang Municipal Hospital (approval number: zzslyykyII20241101003). The Ethics Committee allows a tumor burden of 3 cm or approximately 15% of the body weight in mice. The maximum tumor burden recorded in the current study did not surpass 20% of the body weight.

#### 2.2.6 Grouping and treatment

By employing the SPSS random number generator, the mice were randomly assigned to a model control group (Group A), an experimental group (Group B), and a negative control group (Group C), with 5 mice per group after excluding mice that succumbed to tumor ulceration, infection, and fighting. After the RCC mouse models were established, the growth of the xenografts was closely monitored. Approximately 15 days were required for the xenografts to reach a size of 5–6 mm^3^. The model group was administered normal saline via gavage, the lathyrol experimental group was gavaged with 25 mg/kg lathyrol solution once daily, and the negative control group was intraperitoneally injected with 2 mg/kg paraplatin on days 0, 3, 7, 10, and 14, twice per week. After 14 days of treatment, the mice were anesthetized, blood was collected from the eyeball, and the xenografted tumor mass was isolated, with impurities and contaminated blood removed by washing with PBS.

#### 2.2.7 HE staining to observe the morphology of RCC xenograft tumors

The xenograft samples were fixed with 4% paraformaldehyde, dehydrated after 72 hours of fixation, embedded in paraffin, sectioned, and stained according to the steps outlined in the HE staining kit, after which the pathological changes in RCC Renca xenograft tumor tissues at high and low magnification were observed under an optical microscope.

#### 2.2.8 RT–PCR to detect gene expression

After washing with PBS, adding TRIzol, adding chloroform and shaking, the samples were centrifuged and stratified. Isopropanol was added to the water phase to precipitate RNA, which was washed with ethanol and centrifuged to remove the supernatant. The RNA was dried and dissolved in DEPC water, and the concentration and purity were measured before storage. For reverse transcription, the reagents were thawed, mixed and centrifuged, the RNA–primer mixture was prepared, the mixture was denatured at 65°C and placed on ice, the reverse transcription reaction solution was prepared, the mixture was incubated at 37°C for 1 hour, and the mixture was inactivated at 85°C and stored. The PCR mix was prepared, NTC quality control contamination was established, the reaction program for PCR amplification was set, and melting curve analysis was performed. For quantitative PCR data analysis, the ΔΔCt data analysis method was used to analyze the amplification curve and melting curve. The target genes and primers used are listed in **Table 1**.

**Table 1:** Primer sequence table.

Gene	Primer	Sequence (5’-3’)	PCR Products
Mus GAPDH	Forward	ATGGCCTTCCGTGTTCCTAC	167 bp
	Reverse	AAGTCGCAGGAGACAACCTG	
Mus TGF-β1	Forward	TGACGTCACTGGAGTTGTACGG	170 bp
	Reverse	GGTTCATGTCATGGATGGTGC	
Mus TGF-βR1	Forward	TTGCAGACTTGGGACTTGCT	211 bp
	Reverse	CCACCAATAGAACAGCGTCG	
Mus Smad2	Forward	CTACACCCACTCCATTCC	231 bp
	Reverse	GCAGGTTCCGAGTAAGTAA	
Mus Smad3	Forward	GGAACTTACAAGGCGACAC	107 bp
	Reverse	TGGGAGACTGGACGAAA	
Mus Smad4	Forward	AGGCAGAGCATCAAGGAA	116 bp
	Reverse	CAGTCTAAAGGCTGTGGGT	
Mus SMAD6	Forward	CAGCAAGATCGGTTTTGGCAT	293 bp
	Reverse	AGGAGGTGATGAACTGTCGC	
Mus Smad9	Forward	CCATACCATTACCGCAGAGTG	155 bp
	Reverse	TCAGGGTAGGTGGCGTTGT	

#### 2.2.9 Western blot analysis of protein expression in Renca RCC cells and tumor xenograft tissues

Sufficient Renca cell samples and clean rice-sized mouse Renca xenograft samples were removed, the prepared PIPA lysis buffer was added, and the cells were lysed on ice to extract proteins. The protein content was determined by the BCA method, after which the proteins were separated by SDS–PAGE, transferred to polyvinylidene fluoride membranes (PVDF membranes), soaked in TBST (containing 5% skim milk powder), and incubated overnight on a shaker at 4°C. The antibody concentrations used for the *in vivo* and *in vitro* experiments are shown in **Tables 2** and **3**. The HRP-labeled secondary antibody was incubated on a 37°C room temperature shaker for 2.5 hours, after which ECL immunoblotting was performed. The belts were scanned, and the gray values of the belts were quantitatively analyzed.

**Table 2:** Dilution of WB primary antibodies (*in vitro*).

Primary antibody	Dilution ratio
TGFβ1	1:1000
TGF-βR1	1:1000
Smad2	1:5000
Smad3	1:6000
Smad4	1:1000
CyclinE1	1:5000
CDK1	1:1000
GAPDH	1:1000

**Table 3:** Dilution of WB primary antibodies (*in vivo*).

Primary antibody	Dilution ratio
Smad4	1:1000
Ki-67	1:1000
PCNA	1:3000
GAPDH	1:1000

#### 2.2.10 Immunohistochemistry to detect protein expression in RCC cells and RCC xenografts

The fixation, dehydration, and embedding procedures for RCC xenograft tissues were the same as those used in the HE staining steps. In accordance with the instructions supplied in the IHC kit manual, 0.01 M citrate buffer was added for antigen repair, endogenous peroxidase activity was blocked with 3% H2O2, and primary antibody was added and incubated overnight at 4°C. The dilution ratios of the primary antibodies are shown in **Tables 4**, **5**. On the second day, HRP-labeled secondary antibody was added, followed by DAB color development, Mayer hematoxylin counterstaining, conventional dehydration and transparency, and neutral gum sealing. For immunocytochemistry (ICC) and IHC, images of 2 random fields of view were acquired for each section, and the average optical density (AOD) value was quantitatively analyzed using ImageJ software.

**Table 4:** Dilution of IHC primary antibodies (*in vitro*).

Primary antibody	Dilution ratio
Smad6	1:500
Smad9	1:500
CyclinD1	1:500
CyclinA1	1:500
CyclinB1	1:300
CDK2	1:200
CDK4	1:400
CDK6	1:200
P16	1:500
P21	1:100
P27	1:200
Ki67	1:500
PCNA	1:500

**Table 5:** Dilution of IHC primary antibodies (*in vivo*).

Primary antibody	Dilution ratio
TGFβ1	1:500
TGF-βR1	1:500
Smad2 + 3	1:500
Smad6	1:500
Smad9	1:500
CyclinD1	1:500
CyclinA1	1:500
CyclinB1	1:300
CyclinE1	1:500
CDK1	1:500
CDK2	1:200
CDK4	1:400
CDK6	1:200
P16	1:400
P21	1:100
P27	1:200

### 2.3 Statistical analysis

The data were analyzed via IBM SPSS 26.0, and the data were visualized with GraphPad Prism 9.0. All cell experiments performed in triplicate with independent biological replicates. A difference was deemed statistically significant if P < 0.05, *P < 0.05, **P < 0.01, or ***P < 0.001; otherwise, a difference was considered not significant (ns) if P > 0.05.

## 3 Results

### 3.1 TGF-β/Smad signaling components are highly expressed in RCC and impact the clinical prognosis

**Figure 1:**
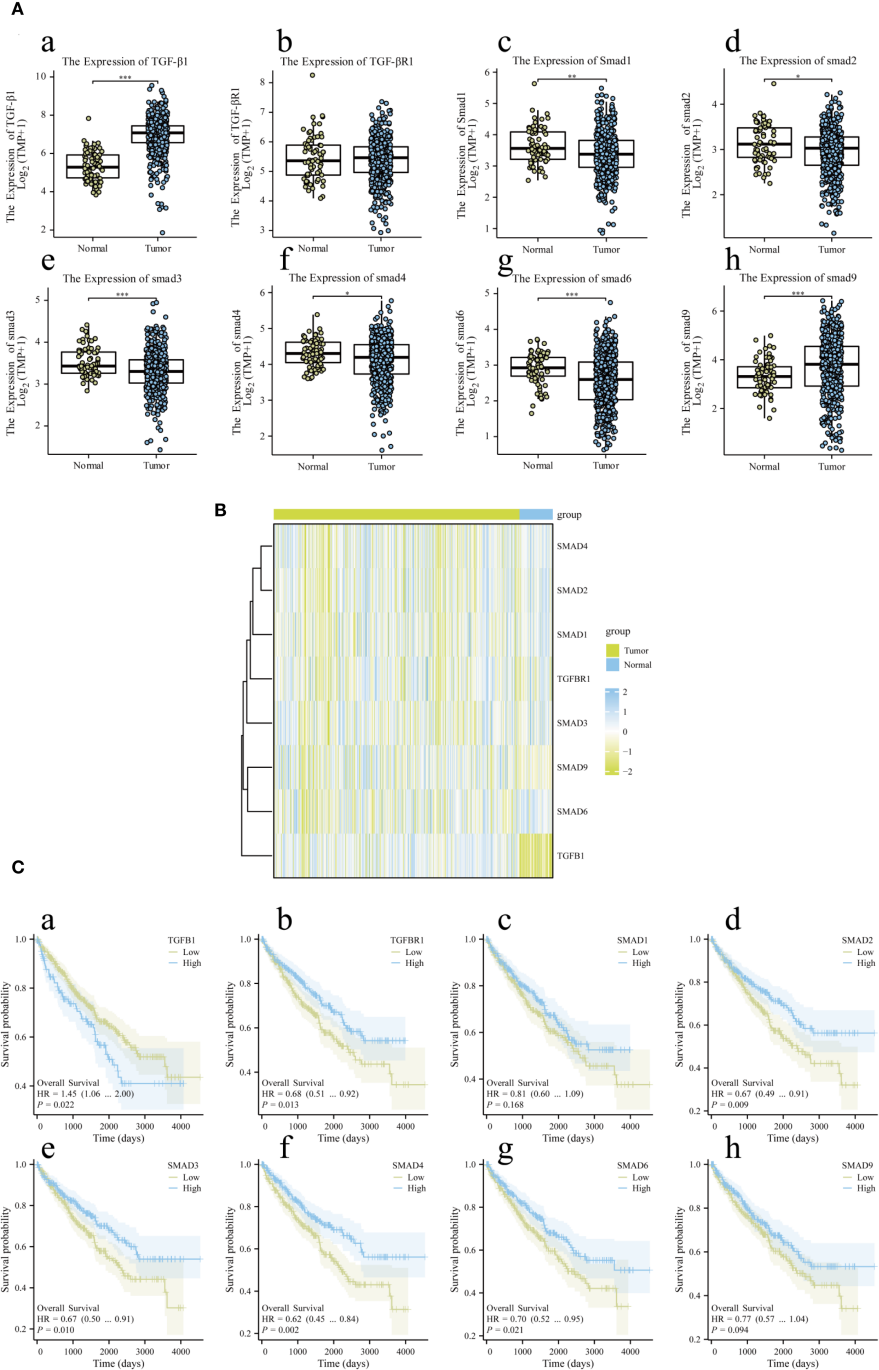
Bioinformatics gene expression and clinical prognosis analysis of the main target proteins of the TGF-β/Smad signaling pathway. Part **(A)** presents the bioinformatics gene expression results for the TGF-β1 (a), TGF-βR1 (b), Smad1 (c), Smad2 (d), Smad3 (e), Smad4 (f), Smad6 (g), and Smad9 (h) proteins, Part **(B)** shows the simple heatmap integration of the gene expression results. Part **(C)** shows the clinical prognosis analysis of the gene expression of TGF-β1 (s), TGF-βR1 (b), Smad1 (c), Smad2 (d), Smad3 (e), Smad4 (f), Smad6 (g), and Smad9 (h). A difference was considered statistically significant if *P* < 0.05, *P < 0.05, **P < 0.01, or ***P < 0.001; alternatively, a difference is considered not significant (ns) if *P* > 0.05.

The RCC sample used to investigate the relative expression level of the target gene included 72 model control group samples and 532 cancer group samples. Integration and analysis of the relevant data revealed that the expression of TGF-β1, Smad1, Smad2, Smad3, Smad4, and Smad9 in malignant tissues was greater than that in the model control group, whereas the expression of Smad6 was significantly higher in the model control group than in the tumor group. There was no significant difference in the expression of TGF-βR1 between the normal and tumor groups. The clinical prognosis analysis of target gene expression (**Figure 1C**) revealed 270 samples in the low-expression group, with a total of 99 events and 171 censored cases, and 271 samples in the high-expression group, with a total of 76 events and 195 censored cases. Clinical prognosis analysis revealed that RCC patients with high expression of Smad1, Smad2, Smad3, Smad4, Smad9, Smad6, and TGF-βR1 had a better prognosis than did those with low expression, while RCC patients with low expression of TGF-β1 had a better prognosis than did those with high expression, and the difference was statistically significant.

### 3.2 Lathyrol limits the proliferation of 786-O cells and Renca cells to comparable extents

**Figure 2:**
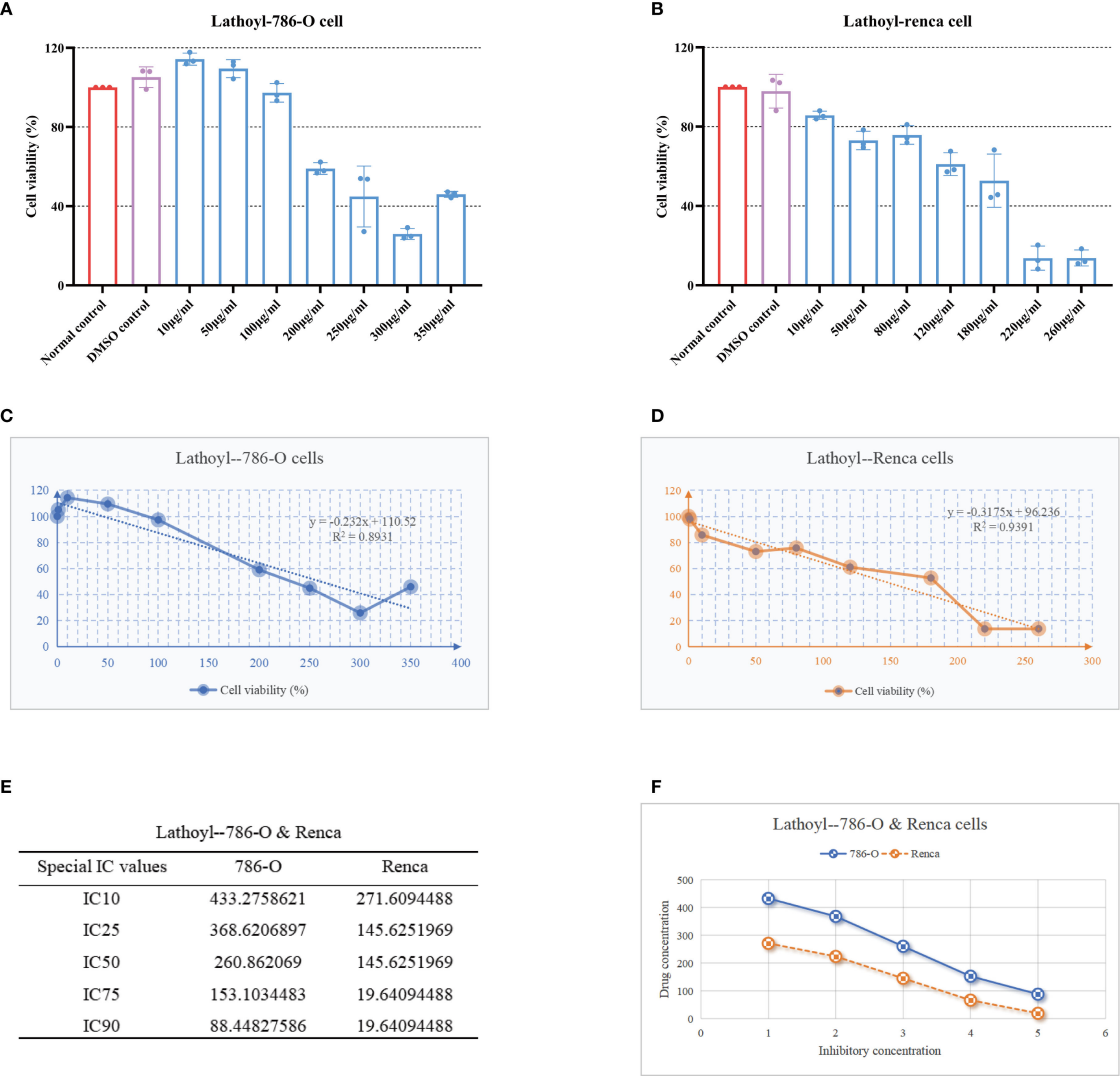
After 24 hours of lathyrol treatment, the activity of these two cells was inhibited in 786-O **(A)** and Renca cells **(B)**. Owing to the poor solubility of lathyrol in water, an appropriate amount of DMSO was used as a cosolvent. In order to evaluate the effect of DMSO on cell viability, we compared the viability of RCC cells cultured in medium supplemented with 1% DMSO with that of RCC cells cultured in normal medium. The results revealed that there was no significant difference in cell viability between the two groups; thus, in subsequent experiments, different concentrations of the drug were added to medium containing 1% DMSO. Parts **(C, D)** show the regression curve equations between the activity of 786-O cells and Renca cells and the drug concentration based on the results of the CCK-8 assay. Part **(E)** calculates the drug concentration required to reach a specific inhibitory concentration (IC value) on the basis of these equations. Finally, part **(F)** performs a statistical analysis on the drug concentration data of the two cells in part **(E)**.

After 24 hours of treatment, we evaluated cell viability by performing a CCK-8 assay. The results indicated that lathyrol could effectively inhibit the proliferation of human RCC 786-O cells (**Figure 2A**) and mouse RCC Renca cells (**Figure 2B**) *in vitro*, and the inhibitory effect was positively correlated with the drug concentration. We subsequently analyzed the average cell viability under different drug concentrations and plotted the regression curve of drug concentration and cell viability (**Figures 2C, D**). As depicted in **Figure 2E**, there was no statistically significant difference between the IC values of human RCC cancer 786-O cells and mouse RCC cancer Renca cells (*t*=-1.447; *P*=0.186), suggesting that the therapeutic effects of lathyrol on these two cell lines were comparable (**Figure 2F**). To simulate the tumor microenvironment more precisely, we selected mouse RCC Renca cells to construct xenograft animal models for subsequent *in vitro* and *in vivo* experiments.

### 3.3 Lathyrol represses the protein and gene expression of factors involved in the TGF-β/Smad signaling pathway in Renca RCC cells *in vitro*

**Figure 3:**
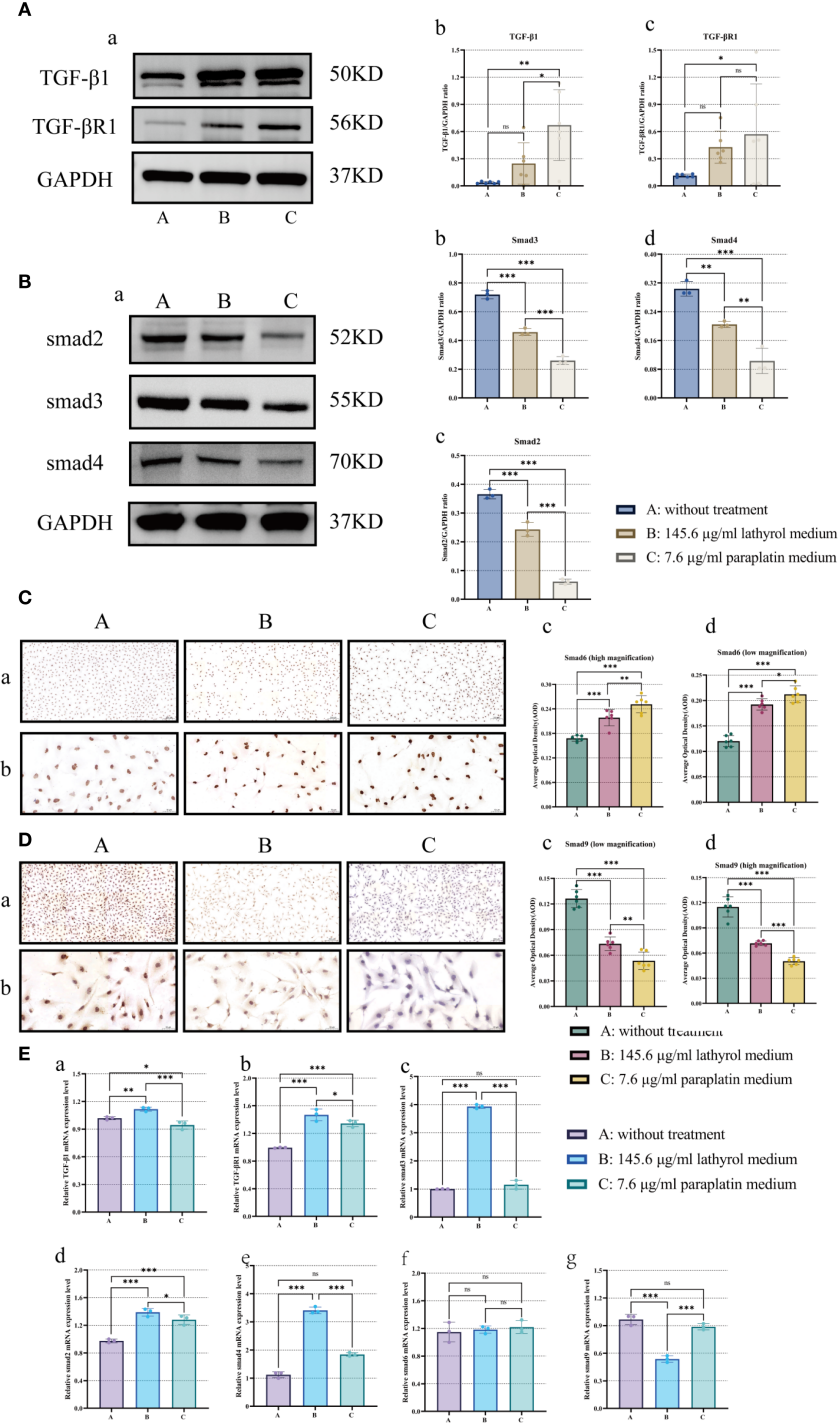
Experimental analysis of the effects of lathyrol on the expression of TGF-β/Smad pathway proteins and mRNA in Renca cells. Part **(A)** presents the results of the detection of TGF-β1 and TGF-βR1 protein expression, which was determined by WB. (a) represents the WB band, while b and c constitute the statistical analysis. Part **(B)** displays the results of Smad2, Smad3, and Smad4 protein expression, using an experimental approach identical to that in **(A)** a indicates the WB band, and (b, c), and d represent the statistical analysis. Part **(C)** shows the detection results of smad6 protein expression, where the experimental method is the ICC. a is the result of low magnification, b at high magnification, and c and d show the statistical analysis of the AOD value. Part **(D)** presents the results obtained for Smad9 protein expression, with the experimental method and parts (a–c), and d being the same as those in **(C)** Part **(E)** shows the mRNA expression results for the TGF-β1 (s), TGF-βR1 (b), Smad2 (c), Smad3 (d), Smad4 (e), Smad6 (f), and Smad9 (g) proteins in Renca cells. A difference was considered statistically significant if *P* < 0.05, *P < 0.05, **P < 0.01, or ***P < 0.001; alternatively, a difference is considered not significant (ns) if *P* > 0.05.

Lathyrol could reduce the protein and gene expression of factors involved in the TGF-β/Smad signaling pathway in Renca RCC cells *in vitro* (**Figure 3**). After 24 hours of treatment, the gene expression trends of TGF-β1, TGF-βR1, Smad2, Smad3, Smad4, Smad6, and Smad9 in Renca cells were partially distinct from the protein expression trends. Compared with group A, the protein and mRNA expression of TGF-β1 and TGF-βR1 in groups B and C was increased (**Figures 3A, Ea, b**). The mRNA and protein expression trends of these two proteins were consistent. The expression trends of the Smad series of proteins were dissimilar to the trends of their mRNA expression. After treatment, lathyrol (group B) and paraplatin (group C) increased the mRNA expression of Smad2, Smad3, and Smad4 in Renca cells (**Figures 3Ec, d**) but decreased the expression of Smad2, Smad3, and Smad4 (**Figure 3B**); however, lathyrol decreased the expression of Smad9 mRNA, and paraplatin had a minimal effect on Smad9 expression (**Figure 3Eg**). However, both drugs inhibited the expression of Smad9 protein (**Figure 3D**). Lathyrol and paraplatin had no impact on Smad6 mRNA expression (**Figure 3Ef**), but both could increase the expression of Smad6 protein (**Figure 3C**). This finding suggested that lathyrol and paraplatin might exert their anti-RCC cell effects by influencing the synthesis and expression of the TGF-β/Smad signaling pathway proteins in RCC cells, rather than affecting the transcription of the genes of the TGF-β/Smad pathway proteins.

### 3.4 Lathyrol increases the expression of cyclin proteins in Renca cells *in vitro* and blocks cell cycle progression

**Figure 4:**
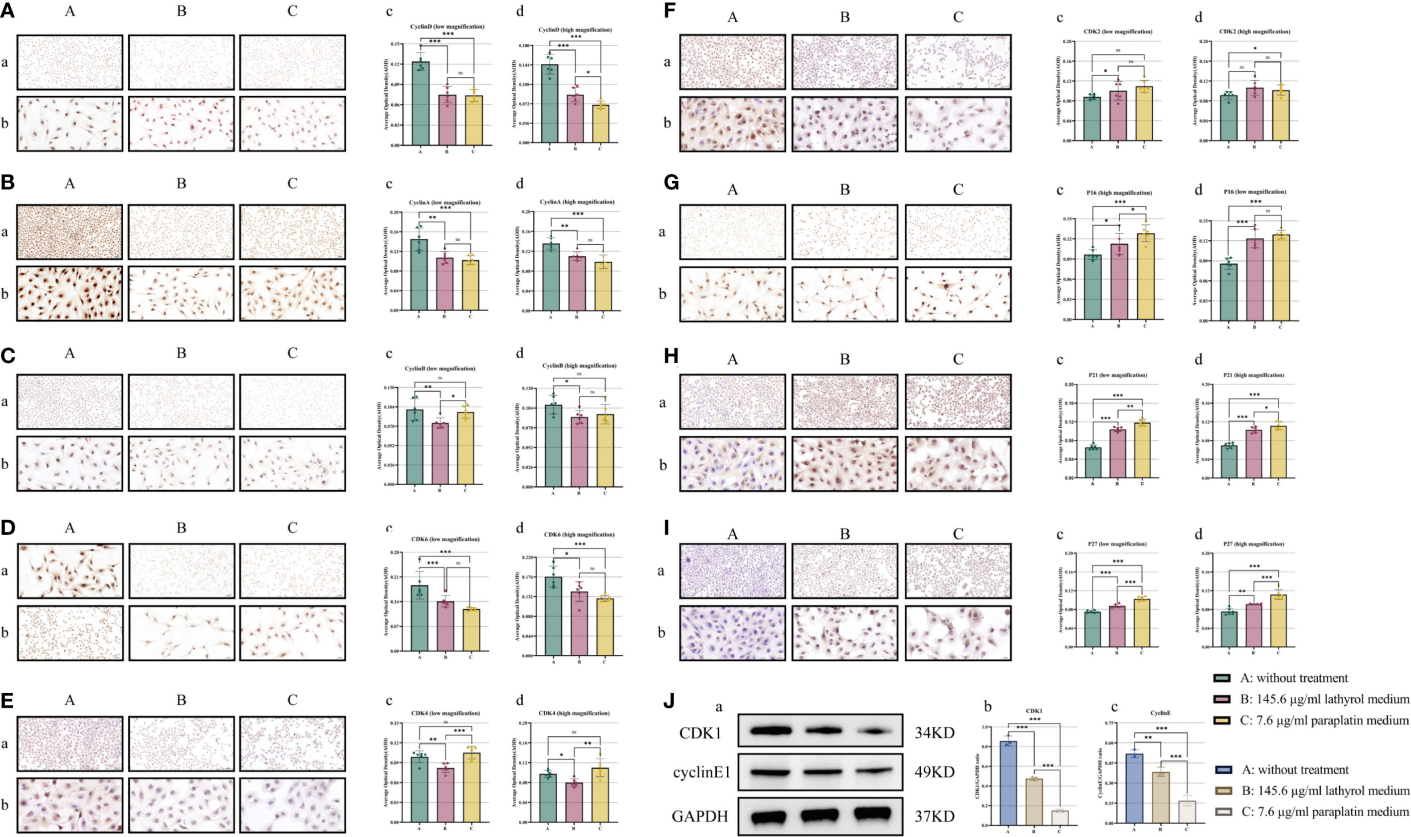
ICC and WB experiments were conducted to examine the effects of lathyrol on the expression of cell cycle regulatory proteins. The outcomes of the ICC experiment are displayed for components **(A–I)**, where a depicts the observational findings under a low-power microscope, while b shows the results under a high-power microscope; c and d represent the statistical analysis results of the AOD value. Part **(J)** presents the results of the WB experiment. Panel a shows the WB bands, and panels b and c illustrate the corresponding statistical analysis. **(A)** cyclin D1, **(B)** cyclin A1, **(C)** cyclin B1, **(D)** CDK6, **(E)** CDK4, **(F)** CDK2, **(G)** P16, **(H)** P21, **(I)** P27, and **(J)** cyclin E1 and CDK1. A difference was considered statistically significant if *P* < 0.05, *P < 0.05, **P < 0.01, or ***P < 0.001; alternatively, a difference is considered not significant (ns) if *P* > 0.05.

The results (**Figure 4**) demonstrated that both lathyrol and paraplatin effectively suppressed the expression of cyclin proteins in RCC cells *in vitro*. Among the proteins in the cyclin series, lathyrol could decrease the protein expression of cyclin D1, cyclin B1, cyclin A1, and cyclin E1 in Renca cells. In contrast, paraplatin inhibited the protein expression of cyclin D1, cyclin A1, and cyclin E1 in these cells. Among the CDK proteins, lathyrol could inhibit the expression of the CDK6, CDK4, and CDK1 proteins in Renca cells, whereas paraplatin reduced the expression of the CDK6 and CDK1 proteins in these cells. Among the cyclin-dependent kinase inhibitor (CKI) proteins, P16, P21, and P27, the three cell cycle inhibitory proteins, were expressed at significantly higher levels in groups B and C cells than in group A. Lathyrol did not affect CDK2 expression, and paraplatin did not affect cyclin B1, CDK4, or CDK2 protein expression. These findings suggested that lathyrol and paraplatin might exert anti-RCC effects by influencing the expression of cell cycle proteins.

### 3.5 Lathyrol reduces the proliferation of RCC carcinomas xenograft

**Figure 5:**
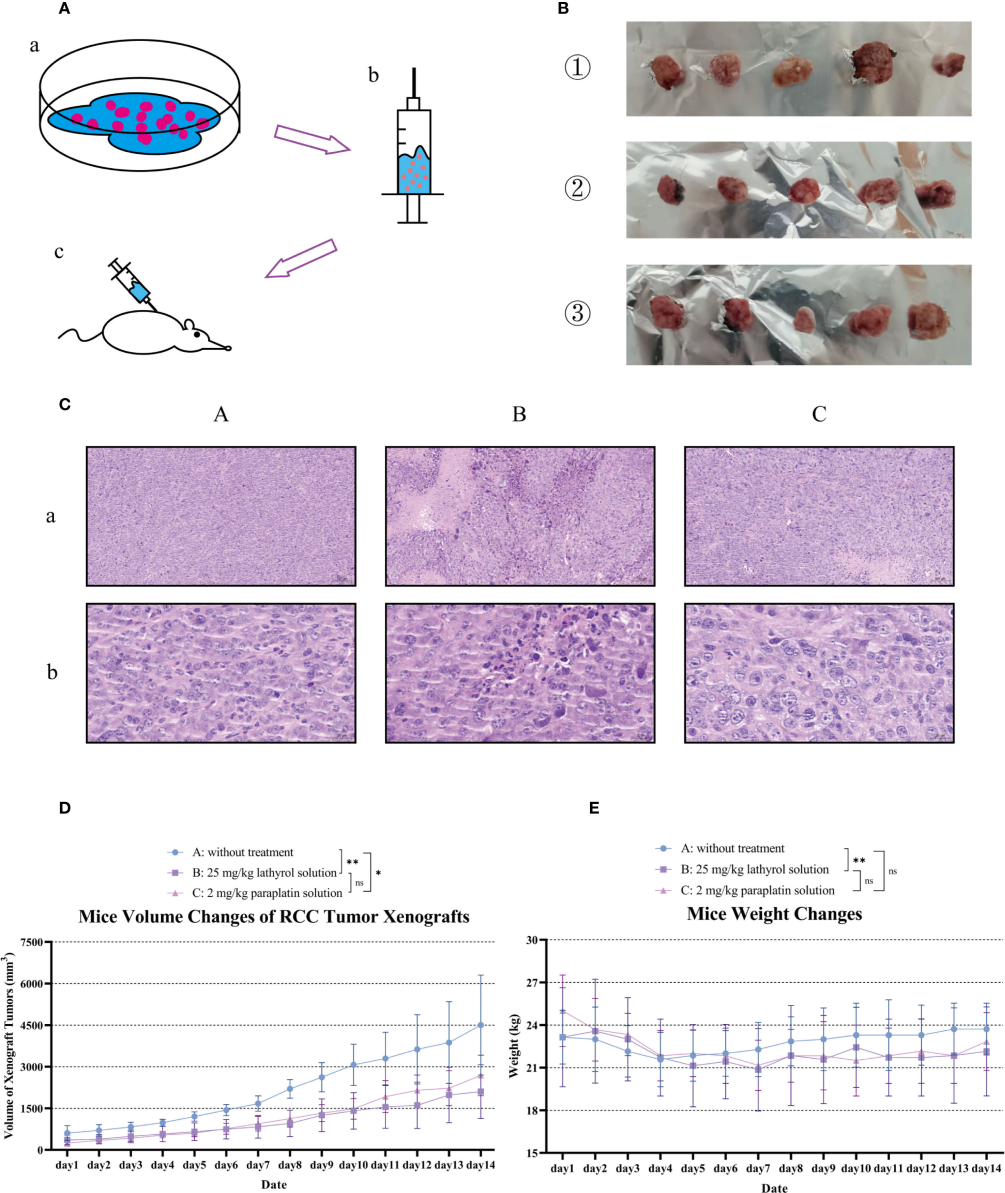
Construction and evaluation of therapeutic results in a mouse RCC model. Part **(A)** describes the construction process of the mouse RCC model. First, the cells were digested with trypsin, and appropriate amounts of mouse RCC Renca cells were collected. Then, these cells were injected subcutaneously into the axilla of the mice. After more than one week, the formation of xenografts was observed in the axilla of the mice. When the RCC xenograft volumes of mice in each experimental group were similar, treatment was initiated. Part **(B)** shows the RCC xenograft samples isolated from mice in each group after 14 days of treatment. Among them, ① represents the RCC xenograft samples of mice in group A, ② represents those in group B, and ③ represents those in group C. This figure was previously presented in the author’s (Shengyou Song) unpublished postgraduate dissertation. Part **(C)** shows the analysis of hematoxylin-eosin (HE) pathological staining of RCC xenograft tumors in each group of mice. Part **(D)** presents the growth curves of RCC xenografts in each group of mice to evaluate the growth dynamics of the cancers. Part **(E)** shows the weight changes of the mice in each group during the experiment. A difference was considered statistically significant if P < 0.05, *P < 0.05, **P < 0.01, or ***P < 0.001; alternatively, a difference is considered not significant (ns) if *P* > 0.05.

After 14 days of treatment, the xenograft volume–growth curve of the mice (**Figure 5D**) revealed the diminution effects of lathyrol and paraplatin on the proliferation of RCC xenograft tumors in the mice. Specifically, the volume growth trend of the xenografts of mice in Group A was reduced compared with those in groups B and C, and this difference was statistically significant. Additionally, the body weights of mice in group B were significantly lower than those in group A, suggesting that lathyrol might have a weight reduction side effect in mice (**Figure 5E**). Based on HE pathological section analysis (**Figure 5C**), the RCC xenografts in groups A, B, and C all exhibited high differentiation characteristics. The tumor cells were closely arranged, with large nuclei and deep staining. The terminal branches of the blood vessels were abundant. The nuclei were deeply stained and appeared bluish black, while the cytoplasm showed varying degrees of pink. Under low-power microscopy, rich neovascularization was observed in the xenograft tissues of each group, and the neoplasm was well encapsulated, dividing the cells into nest-like structures. Under high-power microscopy, significant atypia of tumor cells was visible, and the nuclei presented pathological nuclear division. Of note, some xenograft tissues in groups B and C exhibited phenomena such as necrosis, limited proliferation, nuclear fragmentation, and pyknosis.

### 3.6 Lathyrol decreases the expression of proteins involved in the TGF-β/Smad signaling pathway and genes in RCC xenografts

**Figure 6:**
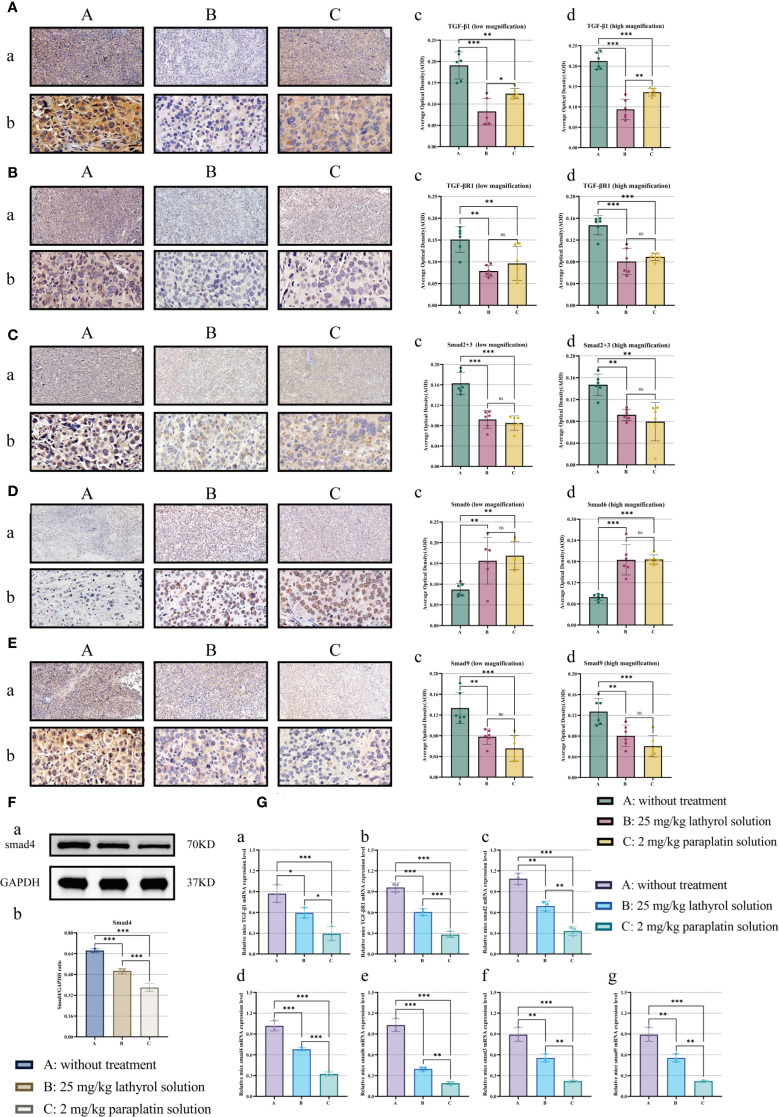
Part **(A)** shows the results of the detection of TGF-β1 protein expression using IHC. a represents the results at low magnification, b represents the results at high magnification, and c and d represent the statistical analysis of the AOD values. Part **(B)** shows the detection results of TGF-βR1 protein expression, using the same experimental method and a, b, c, and d as in **(A)** Part **(C)** shows the detection results obtained for Smad2 + 3 protein expression, using the same experimental methods and a, b, c, and d as in **(A)** Part **(D)** shows the detection results obtained for Smad6 protein expression, using the same experimental method and a, b, c, and d as in **(A)** Part **(E)** shows the detection results determined for E protein expression, using the same experimental methods and a, b, c, and d as in **(A)** Part **(F)** shows the WB experimental results, in which a shows the WB band, and b and c show the corresponding statistical analysis. Part **(G)** shows the mRNA expression results obtained for the TGF-β1 (a), TGF-βR1 (b), Smad2 (c), Smad3 (d), Smad4 (e), Smad6 (f), and Smad9 (g) proteins in Renca cells. A difference was considered statistically significant if *P* < 0.05, *P < 0.05, **P < 0.01, or ***P < 0.001; alternatively, a difference is considered not significant (ns) if *P* > 0.05.

After 14 days of drug treatment, the mRNA expression level of Smad6 in RCC xenografts of mice in groups A was lower than that in groups B and C; concurrently, the mRNA expression levels of TGF-β1, TGF-βR1, Smad2, Smad3, Smad4, and Smad9 in RCC xenografts of mice in groups B and C were also reduced compared with those in group A (**Figure 6**). Using WB and ICC detection, we observed that the protein expression levels of TGF-β1, TGF-βR1, and Smad6 in RCC xenografts of mice in group A were lower than those in groups B and C, while the protein expression levels of Smad2, Smad3, Smad4, and Smad9 in RCC xenografts of mice in groups B and C were also lower than those in group A. Compared with the results under *in vitro* experimental conditions (**Figure 3**), under *in vivo* experimental conditions, the mRNA expression levels of these proteins in treated Renca cells were consistent with the tends observed for the protein. These findings suggested that lathyrol and paraplatin might attenuate the activity of the TGF-β/Smad signaling pathway by influencing the expression of TGF-β/Smad signaling pathway proteins and inhibiting the mRNA transcription of these genes *in vivo*.

### 3.7 Lathyrol curtails the expression of cyclin in RCC xenografts and blocks the cell cycle

**Figure 7:**
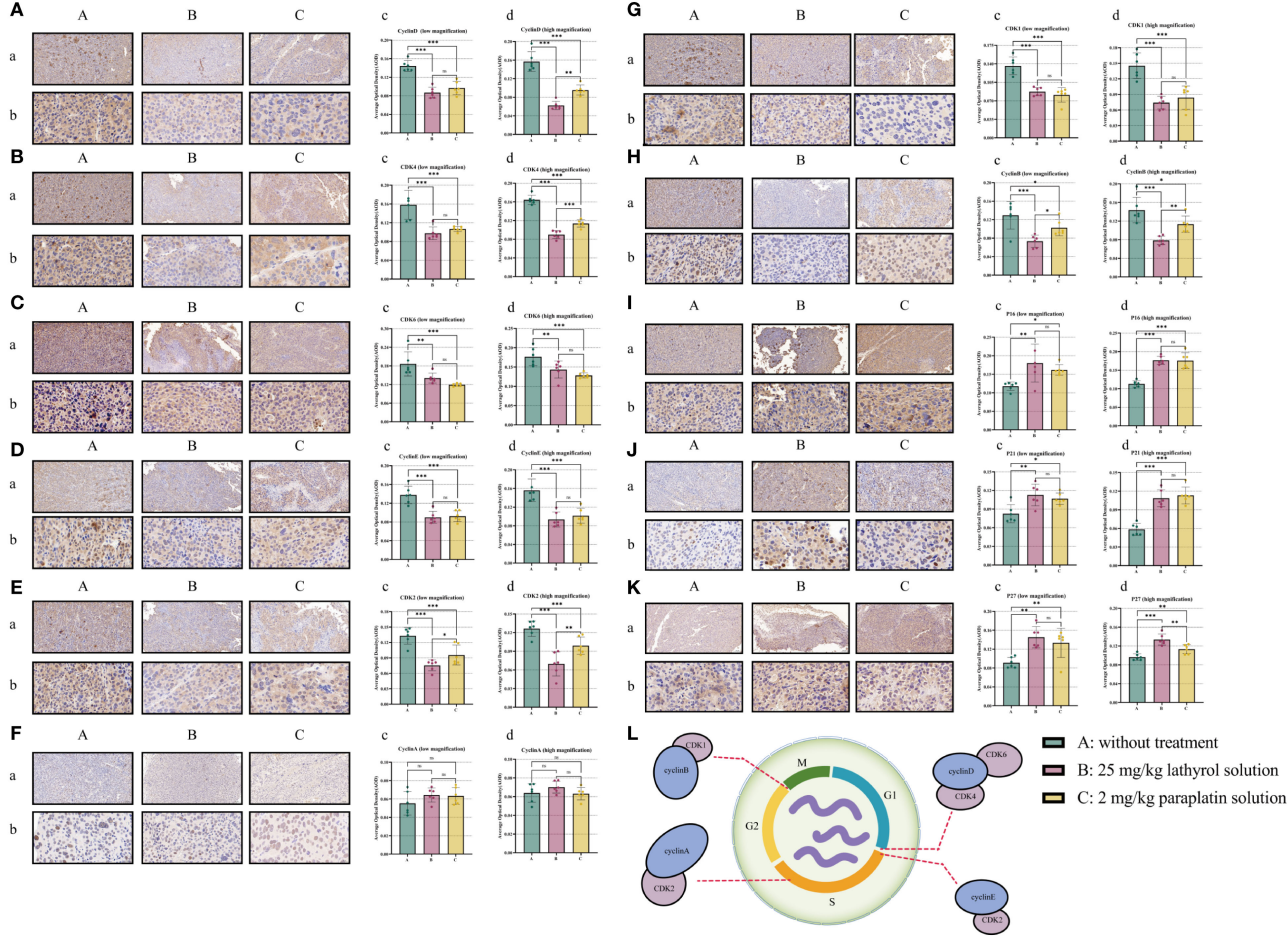
Parts **(A–L)** present the results of the IHC experiment. Part a displays the results under a low-power microscope, and b shows the results under a high-power microscope; c and d represent the statistical analysis of the AOD value. **(A)** cyclin D1, **(B)** CDK4, **(C)** CDK6, **(D)** cyclin E1, **(E)** CDK2, **(F)** CDK2, **(G)** CDK1, **(H)** cyclin B1, **(I)** P16, **(J)** P21 and **(K)** P2. Part **(L)** shows a schematic diagram of the cell cycle. A difference was considered statistically significant if *P* < 0.05, *P < 0.05, **P < 0.01, or ***P < 0.001; alternatively, a difference is considered not significant (ns) if *P* > 0.05.

After treatment with lathyrol and paraplatin, the expression of cyclinD1, cyclinB1, cyclinE1, CDK4, CDK6, CDK2, and CDK1 in group A RCC xenografts was greater than that in groups B and Cwhereas the expression of P16, P21, and P27 in groups B and C was greater than that in group A. However, lathyrol and paraplatin had a negligible effect on the expression of cyclin A1 protein in Renca cells *in vivo*, and the cell cycle might be blocked in the G1 phase. These findings suggested that lathyrol and paraplatin might affect the S phase of the cell cycle (**Figure 7L**), but they could still affect anti-RCC cells by altering the expression of cyclins.

### 3.8 Lathyrol inhibits the expression of PCNA and KI67 protein in RCC cells

**Figure 8:**
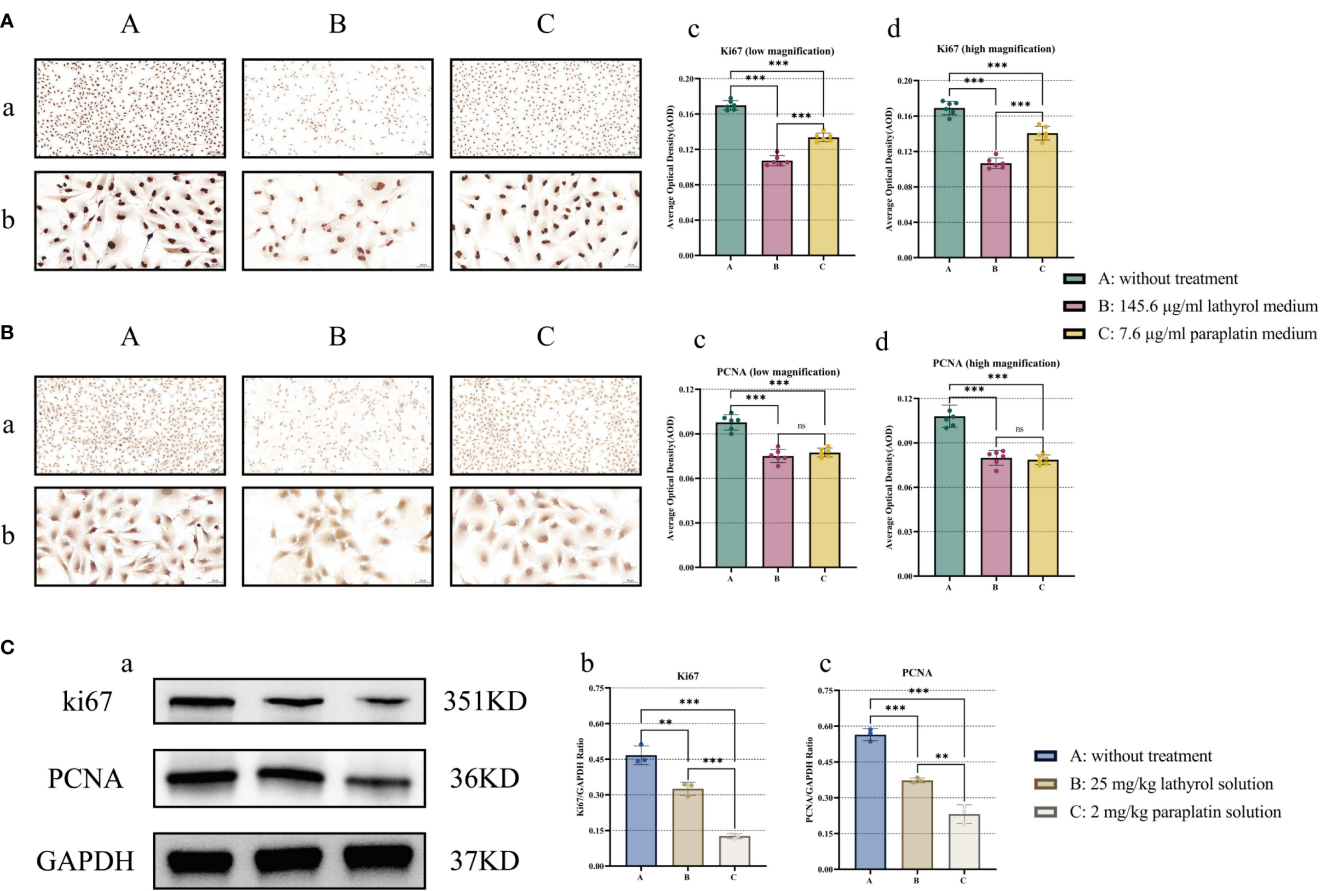
Components **(A, B)** present the outcomes of the ICC experiment, where a depicts the results under a low-power microscope, and b shows the results under a high-power microscope; c and d represent the statistical analysis of the AOD value. The results of the WB experiments are shown in panels **(C)**, where a is the band corresponding to the WB results and b and c are the results of the statistical analysis of the expression of the Ki67 and PCNA proteins. **(A)** ki67, **(B)** PCNA, and **(C)** Ki67 and PCNA *in vivo*. A difference was considered statistically significant if *P* < 0.05, *P < 0.05, **P < 0.01, or ***P < 0.001; alternatively, a difference is considered not significant (ns) if *P* > 0.05.

The PCNA protein is predominantly expressed in the cytoplasm, whereas the Ki67 protein is expressed in both the cytoplasm and the nucleus. After *in vivo* and *in vitro* experimental manipulations with lathyrol and paraplatin, the expression of PCNA and Ki67 proteins in RCC cells in groups B and C was repressed (**Figure 8**). These findings suggested that lathyrol could efficaciously decrease the protein expression of PCNA and Ki67 in RCC cells *in vivo* and *in vitro*, thereby impeding the proliferation of RCC cells and xenografts.

## 4 Discussion

In current medical practice, the treatment of RCC relies mainly on surgical resection. After surgery, doctors decide whether to use tyrosine kinase inhibitors (TKIs) such as sunitinib and imatinib for adjuvant therapy on the basis of the results of the pathological examination (16). These first-line clinical anticancer drugs and targeted drugs can efficaciously obstruct the growth and spread of neoplastic cells, thereby improving their therapeutic role. For patients who cannot tolerate surgical treatment, that is, those who have developed to advanced stages and have metastasis, radical surgical resection is impossible in the advanced stage of the tumor, and additional alternative treatment methods are needed (16, 17). Although there are relatively complete regimens for targeted therapy, immunotherapy, and combined targeted immune therapy for patients with RCC (18, 19), in actual clinical work, the side effects of treatment are often intolerable, causing patients to treatment (20, 21). Drug-related damage during treatment sometimes endangers the stability of vital signs, while traditional Chinese medicine has stable efficacy and better safety in this context (22–24). However, with the widespread application of anticancer drugs, some neoplastic cells may develop resistance to certain drugs, resulting in decreased drug sensitivity (25). The emergence of this resistance will significantly reduce the anti-tumor efficacy of patients, introducing new challenges to the treatment of RCC patients (26). Therefore, the medical community must continuously research and develop new drugs and treatment methods to address the problem of neoplasia resistance and improve the survival rate and quality of life of patients.

In this study, our objective was to validate the anticancer activity of lathyrol, using paraplatin as the control drug. Paraplatin, a second-generation platinum-based anticancer medication that is widely employed in clinical settings, is typically utilized in combination with other drugs to constitute a combined chemotherapy regimen for the management of related cancer disorders (27, 28). Its anticancer mechanism primarily involves interactions with DNA. Upon entering cells, paraplatin can establish cross-links with DNA molecules, particularly interstrand cross-links, thereby interfering with the normal replication and transcription processes of DNA (29). Its anti-cancer mechanism primarily entails the interaction with DNA. Upon entering the cell, paraplatin can establish cross-links with DNA molecules, particularly interstrand cross-links, thereby interfering with the normal replication and transcription processes of DNA (30, 31). Additionally, the DNA damage caused by paraplatin activates the intracellular DNA damage response mechanism, including the activation of the p53 protein (31), which, in turn, triggers the apoptotic program and inhibits the proliferation of cancer cells, leading to cancer cells deaths (30, 32). These mechanisms of paraplatin render it an efficacious drug for the treatment of diverse cancers (33, 34). Hence, to compare the anticancer effect and mechanism of lathyrol, we employed paraplatin as a control to verify the treatment outcomes of lathyrol against RCC. In a preliminary investigation, we discovered that lathyrol could inhibit the activity of RCC cells both *in vitro* and *in vivo*, and we established a regression equation between cell activity and drug concentration on the basis of the results of the CCK-8 experiment. Concurrently, we compared the specific IC values of human RCC 786-O cells and mouse RCC Renca cells, and the findings revealed no significant difference between the two, suggesting that the therapeutic effects of lathyrol in 786-O and Renca cells were comparable. Therefore, to more realistically simulate the *in vivo* environment, Renca cells and partially immunosuppressed BALB/c mice were ultimately selected to construct an RCC xenograft animal model, and subsequent experiments were conducted to more clearly detect the phenotypic attenuation of lathyrol in the corresponding species in the *in vivo* environment for the treatment of RCC xenografts.

In the TGF-β/Smad signaling pathway transduction system, TGF-β1 and TGF-βR1, which serve as the initiating elements of this pathway, play crucial roles in the activation and function of the entire pathway (9, 35); moreover, Smads 2, 3, and 4 are significant cascade effector factors of this pathway, connecting the precedent and subsequent pathway, and the Smad2/3/4 complex is also an essential protein that enters the nucleus and performs the regulatory transcriptional function of this pathway (36, 37). Smads 6 and 7 have similar functions, primarily playing roles in negative feedback regulation and reducing signal transduction (38, 39). The mechanism through which Smad9 promotes tumorigenesis and development remains unclear; however, its expression in tumors is directly proportional to tumor malignancy and inversely proportional to patient prognosis (40–42). Using *in vitro* experimental conditions, we assessed the expression of certain proteins involved in the TGF-β/Smad signaling pathway in RCC cells and RCC xenograft tumors. The results demonstrated that following treatment with lathyrol and paraplatin, the expression of essential proteins in the TGF-β/Smad signaling pathway in Renca cells, including Smads 2, 3, 4, and 9, was inhibited. In contrast, the protein expression of TGF-β1, TGF-βR1, and Smad6 tended to increase. Nevertheless, at the gene expression level, the expression pattern of mRNA was distinct from the Western blot detection results at the protein level. After treatment with lathyrol and paraplatin, the mRNA expression of Smads 6 and 9 in Renca cells decreased, whereas the mRNA expression of TGF-β1, TGF-βR1, Smad2, Smad3, and Smad4 increased. *In vivo*, the expression trends of marker proteins and mRNAs related to the TGF-β/Smad signaling pathway in mice with RCC were consistent, indicating that the mRNA and protein expression of TGF-β1, TGF-βR1, Smad2, Smad3, Smad4, and Smad9 decreased, whereas the expression of Smad6 protein increased, although its mRNA expression decreased. Interestingly, lathyrol attenuates the expression of certain proteins in the TGF-β/Smad signaling pathway and promotes the expression of inhibitory Smad (I-Smad) proteins in both *in vivo* and *in vitro*, thereby inhibiting the signal transmission and regulatory function of the TGF-β/Smad pathway in RCC cells and exerting corresponding anticancer effects (43, 44). However, *in vitro*, lathyrol primarily blocks the function of related proteins, whereas *in vivo*, it further retards protein synthesis by inhibiting the mRNA transcription function of genes encoding proteins in the TGF-β/Smad pathway. In addition, published evidence indicates that suppression of the TGF-β/Smad pathway can reduce Smad protein expression. For instance, Kokabu et al. demonstrated that PPM1A phosphatase decreases Smad1/5/8 protein levels by activating the proteasomal pathway, independent of classical dephosphorylation at phosphorylation sites, with PPM1A deficiency enhancing BMP/Smad pathway activity (45). Similarly, Matsuura et al. reported that mutation of CDK phosphorylation sites in Smad3 (Thr8/Thr178/Ser212) enhances its antiproliferative function, while pathway inhibition downregulates Smad3 expression (46). Clinically, Maliekal et al. reported that the loss or mutation of Smad2/4 directly correlates with TGF-β pathway dysfunction in cervical cancer (47).

On the basis of this collective evidence, we detected key TGF-β/Smad pathway protein expression levels as a functional readout. Our experimental results revealed that lathyrol inhibited Smad protein expression but promoted TGF-β1 and TGF-βR1 expression *in vitro* while reducing the expression of their corresponding mRNAs, in contrast with the *in vivo* findings. This apparent paradox aligns with established regulatory principles: SMAD protein expression involves transcriptional induction and post-translational modifications (e.g., ubiquitination-mediated degradation), which may cause protein-level reduction despite increased mRNA expression under specific conditions (48, 49). According to Miyazawa et al., such ligand/receptor induction functions as negative feedback to suppress signaling (48), whereas Moustakas revealed that all Smads (R-/I-Smads) undergo ubiquitin tagging and targeted degradation in nuclear/cytoplasmic compartments, resulting in the formation of autoinhibitory signaling circuits (49). Therefore, we hypothesize that distinct lathyrol mechanisms occur in different environments: (*in vivo*) it suppresses key TGF-β/Smad protein expression by inhibiting mRNA production; (*in vitro*) it promotes Smad degradation via ubiquitin labeling, proteasomal activation, or alternative pathways, thereby inhibiting pathway function while triggering a compensatory upregulation of TGF-β1/TGF-βR1 and Smad mRNAs through negative feedback loops.

In the metabolic activities of cells, ensuring the normal operation of the cell cycle is of vital importance for cell proliferation and functional performance (50). Similarly, in the treatment of malignant diseases, by interfering with the cell cycle process of malignant cells, the proliferation of cancer cells can be effectively reduced, apoptosis can be induced, and malignant behaviors such as the infiltration of cancer cells can be suppressed (51, 52). The TGF-β/Smad pathway has a significant effect on the normal function of the cell cycle (53, 54), and by influencing the signal transduction of the TGF-β/Smad pathway, the cell cycle of tumor cells can be disrupted, thereby suppressing the malignant behavior of carcinoma cells (55, 56), an essential angle we plan to explore. The cell cycle comprises four main stages: the G1 phase before DNA synthesis, the S phase of DNA synthesis, the G2 phase after DNA synthesis, and the M phase of mitosis (57). Throughout the cell cycle, regulatory molecules, including cyclins, cyclin-dependent kinases (CDKs), and cyclin-dependent kinase inhibitors (CKIs) (58), together constitute the cyclin-CDK-CKI regulatory network (59–61). Cyclin protein binds to the corresponding CDK, forming a necessary checkpoint in the cell cycle (62). Using Western blot and immunohistochemistry/immunocytochemistry (IHC/ICC) analysis, we discovered that both lathyrol and paraplatin could affect the expression of cyclin proteins, which play a blocking role in the cell cycle, and also impact the expression of cell cycle regulatory proteins CDK and CKI, indirectly influencing the normal operation of the RCC cell cycle. More specifically, after treatment with lathyrol and paraplatin, protein expression levels of cyclin D1, cyclin E1, and cyclin A1 in Renca cells and mouse xenograft tumors decreased, while those of CDK4, CDK6, CDK2, and CDK1 decreased. In contrast, the expression levels of the CKI proteins P16, P27, and P21 increased. Additionally, the effects of lathyrol and paraplatin on the expression of cyclinB1 protein were relatively minor, indicating that these two compounds mainly had a role in blocking the cell cycle of renal cell carcinoma (RCC) cells before M phase of the cell cycle. Notably, *in vitro* revealed that the restrictive effect of lathyrol on the protein expression of cyclinD1, cyclinE1, and cyclinA1 was not as prominent as that of paraplatin. However, *in vivo*, the inhibitory effect of lathyrol on the protein expression of cyclin D1 was slightly stronger than that of paraplatin, whereas the inhibitory effect on the protein expression of cyclin A1 was weaker than that of paraplatin. With respect to the expression of the cyclin E1 protein, there was no significant difference in the repressive effect of the two on expression. On the basis of these findings, we hypothesize that compared with lathyrol, paraplatin, a widely utilized anticancer drug, may have a more significant therapeutic effect. Moreover, on the basis of the cell cycle schematic diagram (**Figure 7N**), we considered both medications capable of directly hindering RCC cells from entering the G1/S phase, impede DNA synthesis (S phase), and obstruct the G2/M checkpoint of cells *in vitro*, thereby influencing cell cycle progression. In the *in vivo* milieu, paraplatin can also inhibit the expression of cyclin D1 protein in Renca cells, but compared with lathyrol, paraplatin is more efficacious for inhibiting the expression of the cyclin E1 and cyclin A1 proteins. These findings suggested that lathyrol might primarily play a fundamental role in blocking G1 phase of the cell cycle *in vivo*, whereas paraplatin might mainly play a role in blocking S and G2 phases in RCC cells. These outcomes also indicated that lathyrol possessed anticancer effects and mechanisms comparable to those of clinical anticancer drugs both *in vivo* and *in vitro*. Additionally, considering that lathyrol was capable of promoting the expression of P16, P21, and P27 in Renca cells both *in vivo* and *in vitro*, we surmised that this drug compound could enhance the aging process of cancer cells by exerting its inherent tumor suppressor effect and might also impact the senescence-associated secretory phenotype (SASP) in the *in vivo* environment, thereby influencing immune function; that is, it can regulate immune aging and immune function in patients with carcinoma (63).

Ultimately, we undertook an exhaustive examination of the effects of lathyrol and paraplatin on the proliferation phenotype of renal cancer Renca cells. The experimental results demonstrated that both compounds could appreciably impede the proliferation of Renca cells and mouse RCC xenografts. To further validate this discovery, we meansured the expression levels of PCNA and Ki67 (**Figure 8**). The reduction in the expression of crucial markers of cell proliferation further confirmed the anticancer effects of lathyrol and paraplatin on cell proliferation. Additionally, by observing mouse RCC xenografts via HE staining, we discerned that following treatment with lathyrol and paraplatin, partial tissue necrosis and cell lysis occurred in RCC xenografts. The proliferation and apoptosis of cancer cells play crucial roles in tumorigenesis, development, and treatment. In the field of oncology research, the function and mechanism of Ki67, a nuclear protein closely affiliated with the cell cycle, are important. As important indicators of apoptosis and proliferation, in addition to being capable of sensitively reflecting cells in the proliferative stage in tumor tissues, it can also reveal the growth rate and potential invasive capacity of cells. Elevated Ki67 expression is typically associated with high proliferative activity of carcinoma cells and an unfavorable prognosis, and the higher the positive rate of Ki67, the greater is the proportion of tumor cells in the growth cycle, and the greater is the degree of malignancy. Hence, in clinical practice, the expression level of Ki67 has emerged as a significant biomarker for evaluating the malignancy of neoplasms and predicting the prognosis of cancer patients (64, 65). Its expression level can be modulated by diverse intracellular and extracellular factors, such as growth factors, hormones, and cyclins, and may also be influenced by various factors such as tumor type, pathological grade, patient age, and sex, resulting in varying degrees of expression outcomes (66, 67). Proliferating cell nuclear antigen (PCNA) is a protein that plays a pivotal role in the cell cycle, particularly in DNA replication and cell proliferation (68). As a marker of cell proliferation, the expression level of PCNA is generally correlated with the proliferative activity and prognosis of cancers. By interacting with various proteins, PCNA participates in the regulation of DNA replication and the process of DNA damage repair. In neoplastic cells, owing to certain genetic or epigenetic alterations, the expression level of PCNA may be upregulated, leading to accelerated DNA replication, uncontrolled cell proliferation, and ultimately facilitated tumor growth (69). Furthermore, the expression level of PCNA is strongly regulated by the cell cycle, leading to increased G1/S transition and reaching a peak in S phase (70). Moreover, as a marker for assessing the proliferative status of cells, PCNA is closely associated with the proliferative activity and DNA damage repair capacity of cancer cells, and its expression level is frequently utilized as an indicator of tumor prognosis. Like Ki67, a high expression level of PCNA typically implies a high neoplasm recurrence rate and an unfavorable prognosis (71, 72). The *in vitro* experiments revealed that the expression of Ki67 decreased following the treatment of Renca cells with lathyrol and cisplatin. After *in vivo* intervention in mice with RCC xenografts, the protein expression of PCNA and Ki67 decreased. Combined with the results of the CCK-8 assay, these findings suggested that lathyrol could inhibit the proliferative ability of RCC Renca cell xenograft neoplasms and could exert an anticancer effect similar to that of clinical drugs. The underlying mechanism might be related to the influence of lathyrol on the TGF-β/Smad signaling pathway in Renca cells, thereby suppressing the expression of crucial regulatory proteins in the cell cycle.

In conclusion, we initially discovered that lathyrol could impede the signal transduction of the TGF-β/Smad signaling pathway by influencing the expression of elemental proteins within the pathway, arresting the cell cycle progression of Renca cells, and thereby affecting their proliferation. In general, lathyrol and paraplatin significantly inhibited the proliferation of renal cancer Renca cells and mouse RCC xenografts, suggesting a new research direction and potential treatment alternatives for renal cancer therapy. Nevertheless, regarding the application of lathyrol in the treatment of patients with RCC, further exploration is still necessary. On the one hand, solely targeting the TGF-β/Smad signaling pathway for medical intervention may not be the most favorable option. Bioinformatics data indicate that in patients with RCC, low expression of genes related to the TGF-β/Smad signaling pathway may also have a detrimental effect on prognosis and reduce survival rates. Additionally, in terms of gene expression differences, the expression of genes involved in the TGF-β/Smad signaling pathway in normal tissues is sometimes even greater than that in tumor tissues, suggesting that the occurrence of RCC is not always attributed to abnormal activation of the TGF-β/Smad signaling pathway. Hence, further investigations of the mechanism underlying the occurrence and development of RCC are needed. On the other hand, our research has several limitations. Lathyrol, a potential multitarget anticancer drug may affect the cell cycle progression of RCC cells by influencing the signal transduction of the TGF-β/Smad pathway, or by directly influencing the expression of cyclin proteins and the cell cycle regulatory proteins CDK and CKI. Furthermore, although we detected the protein and mRNA expression levels of the marker targets of the TGF-β/Smad signaling pathway, for other targets of lathyrol and paraplatin, as well as the mRNA and protein expression of cell cycle and proliferation phenotype markers, further exploration is still needed.

As medical technology progresses, analyses of the active ingredients of traditional Chinese medicine and its therapeutic mechanism have gradually become clearer and more transparent (73). Through continuous exploration and application of modern medical technology, the potential of traditional Chinese medicine in this treatment field has been further explored and utilized, allowing a deeper understanding of the composition and mechanism of action and thereby providing new treatment ideas and methods for tumor patients (74). Recently, some scholars and researchers have explored traditional Chinese medicine and reported that it can not only directly suppress and kill neoplastic cells through its own active ingredients but also indirectly kill cancer cells by improving the overall physical condition of patients and enhancing the immunity of the body (75). This indirect effect is achieved mainly by improving the immune function of the body, enabling the body’s own immune system to more effectively identify and attack cancer cells, thereby inhibiting cancer proliferation and invasion (76, 77). Several clinical workers have also reported that by combining first-line anticancer drugs and traditional Chinese medicine, the treatment efficacy for cancer patients and prognosis can be improved (78, 79). Therefore, using traditional Chinese medicine to prevent the proliferation and invasion ability of tumors and improve the survival prognosis of patients has become an effective alternative treatment method (80). This method provides not only new hope for patients who are insensitive or intolerant to traditional Western medicine but also an adjuvant treatment method for patients who have already received Western medicine treatment, helping them achieve better recovery and improving quality of life.

However, this study has several limitations that warrant consideration. Notably, our experimental models relied exclusively on murine-derived Renca renal carcinoma cells for both *in vivo* and *in vivo* analyses, without validation in human renal carcinoma cell lines or patient-derived xenograft (PDX) models in immunodeficient mice—an approach that could provide critical insights into species-specific responses. Furthermore, while we demonstrated functional inhibition of the TGF-β/Smad signaling pathway, the precise molecular mechanisms underlying this suppression require further investigation to elucidate potential compensatory pathways. Additionally, although the absence of observable behavioral toxicity is encouraging, a comprehensive safety evaluation necessitates ongoing dedicated toxicology studies addressing long-term metabolic impacts and organ-specific effects. Addressing these questions constitutes a primary focus of our next phase of research, which will substantially strengthen the foundation for advancing lathyrol toward clinical trials and future therapeutic applications in renal carcinoma.

## 5 Conclusion

On the basis of the results of the above analysis, lathyrol exhibits significant anticancer activity both *in vitro* and *in vivo*. Its mechanism of action may involve the modulation of the expression of marker proteins in the TGF-β/Smad signaling pathway. We infer that lathyrol inhibits signal transduction through this pathway, thereby blocking the cell cycle progression of Renca cells and preventing the proliferation of RCC cells.

## Data Availability

The raw data supporting the conclusions of this article will be made available by the authors, without undue reservation.
