# Correction: Lathyrol inhibits the proliferation of Renca cells by altering expression of TGF-β/Smad pathway components and subsequently affecting the cell cycle

**DOI:** 10.3389/fonc.2025.1725790

**Published:** 2025-11-17

**Authors:** Shengyou Song, Yalin Song

**Affiliations:** 1 Department of Education, Shandong Provincial Hospital, Jinan, China; 2 Department of Urology, Zaozhuang Municipal Hospital, Zaozhuang, China

**Keywords:** lathyrol, renal cell cancer, TGF-β/Smad signalingsmad signal pathway, cell cycle, proliferation

In the published article, a caption of **Figure 5B** was missing. The corrected caption of **Figure 5** appears below.

**Figure 5:**
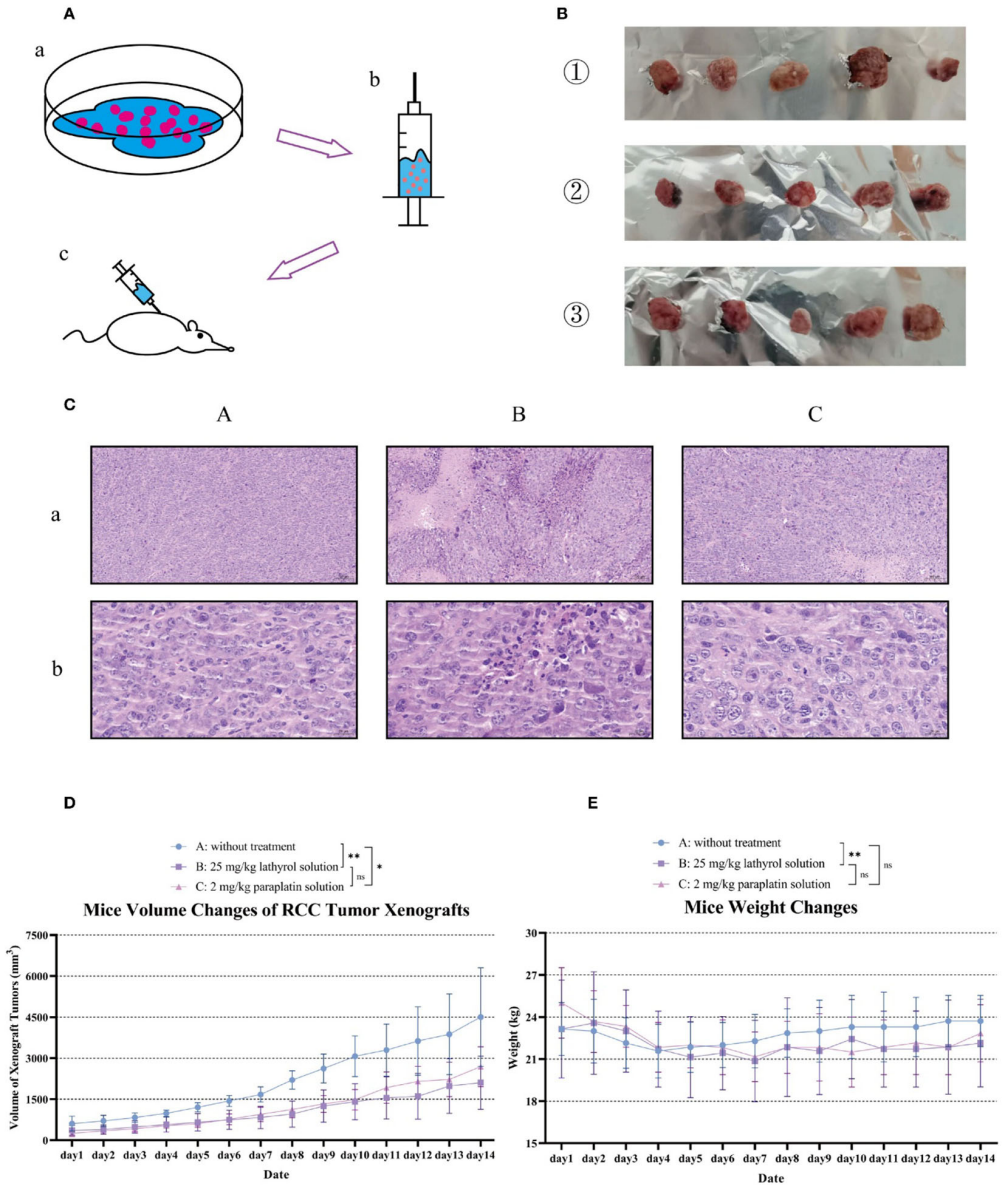
Construction and evaluation of therapeutic results in a mouse RCC model. Part **(A)** describes the construction process of the mouse RCC model. First, the cells were digested with trypsin, and appropriate amounts of mouse RCC Renca cells were collected. Then, these cells were injected subcutaneously into the axilla of the mice. After more than one week, the formation of xenografts was observed in the axilla of the mice. When the RCC xenograft volumes of mice in each experimental group were similar, treatment was initiated. Part **(B)** shows the RCC xenograft samples isolated from mice in each group after 14 days of treatment. Among them, ① represents the RCC xenograft samples of mice in group A, ② represents those in group B, and ③ represents those in group C. This figure was previously presented in the author’s (Shengyou Song) unpublished postgraduate dissertation. Part **(C)** shows the analysis of hematoxylin-eosin (HE) pathological staining of RCC xenograft tumors in each group of mice. Part **(D)** presents the growth curves of RCC xenografts in each group of mice to evaluate the growth dynamics of the cancers. Part **(E)** shows the weight changes of the mice in each group during the experiment. A difference was considered statistically significant if *P* < 0.05, *P < 0.05, **P < 0.01, or ***P < 0.001; alternatively, a difference is considered not significant (ns) if *P* > 0.05.

“Construction and evaluation of therapeutic results in a mouse RCC model. Part (A) describes the construction process of the mouse RCC model. First, the cells were digested with trypsin, and appropriate amounts of mouse RCC Renca cells were collected. Then, these cells were injected subcutaneously into the axilla of the mice. After more than one week, the formation of xenografts was observed in the axilla of the mice. When the RCC xenograft volumes of mice in each experimental group were similar, treatment was initiated. Part (B) shows the RCC xenograft samples isolated from mice in each group after 14 days of treatment. Among them, ① represents the RCC xenograft samples of mice in group A, ② represents those in group B, and ③ represents those in group C. This figure was previously presented in the author’s (Shengyou Song) unpublished postgraduate dissertation. Part (C) shows the analysis of hematoxylin-eosin (HE) pathological staining of RCC xenograft tumors in each group of mice. Part (D) presents the growth curves of RCC xenografts in each group of mice to evaluate the growth dynamics of the cancers. Part (E) shows the weight changes of the mice in each group during the experiment. A difference was considered statistically significant if P < 0.05, *P < 0.05, **P < 0.01, or ***P < 0.001; alternatively, a difference is considered not significant (ns) if P > 0.05.”

The original article has been updated.

