# Supplementary material for: Lathyrol inhibits the proliferation of Renca cells by altering expression of TGF-β/Smad pathway components and subsequently affecting the cell cycle
Source: Front Oncol. 2025 Oct 8;15:1629962. doi: 10.3389/fonc.2025.1629962 (PMC12541422; doi:10.3389/fonc.2025.1629962)
Supplement: Supplementary file 10 [file Supplementaryfile1.pdf]

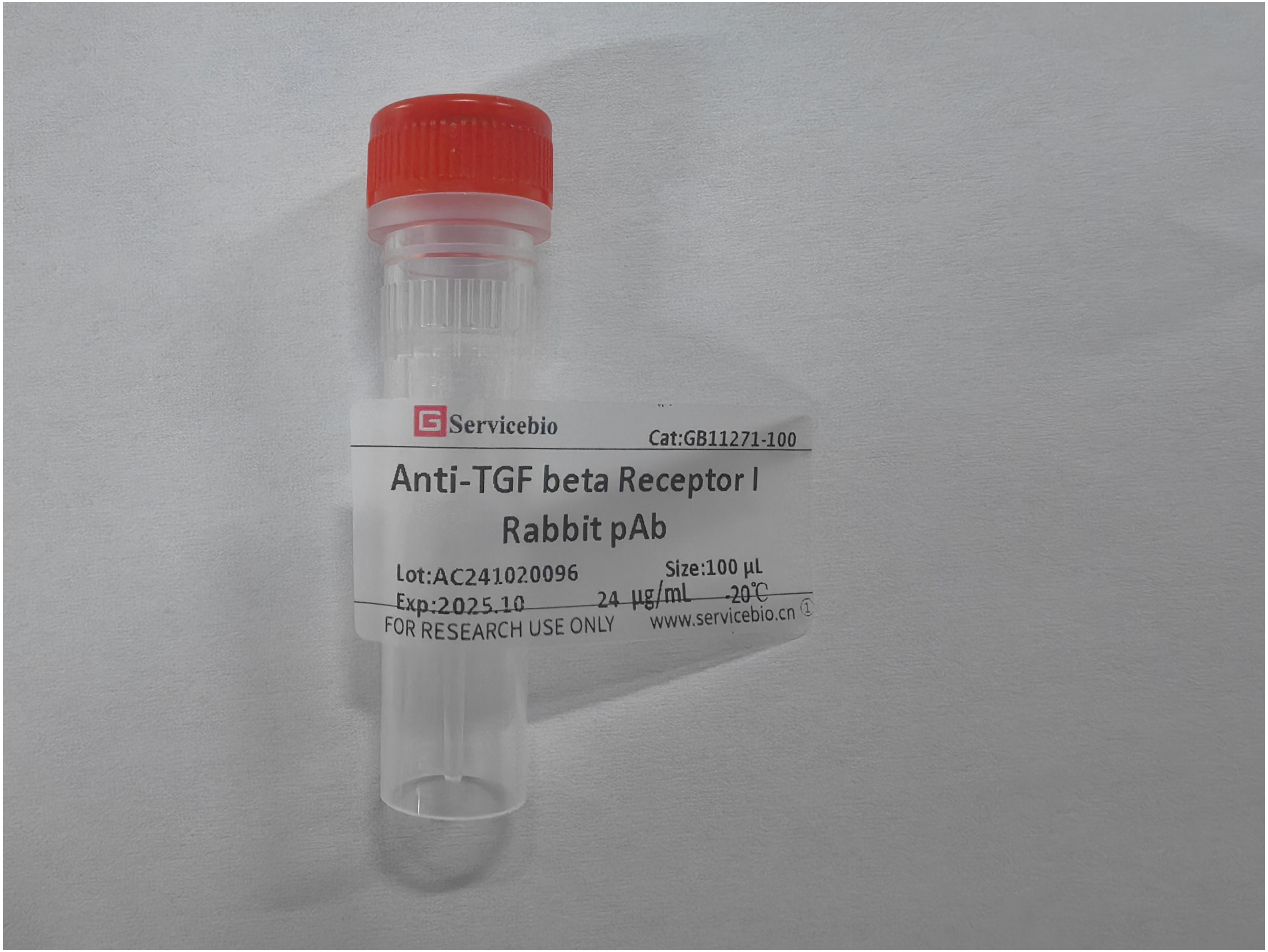
Servicebio
Cat:GB11271-100
Anti-TGF beta Receptor I
Rabbit pAb
Lot:AC241020096
Size:100 μL
Exp:2025.10
24 μg/mL
-20℃
FOR RESEARCH USE ONLY
www.servicebio.cn ①

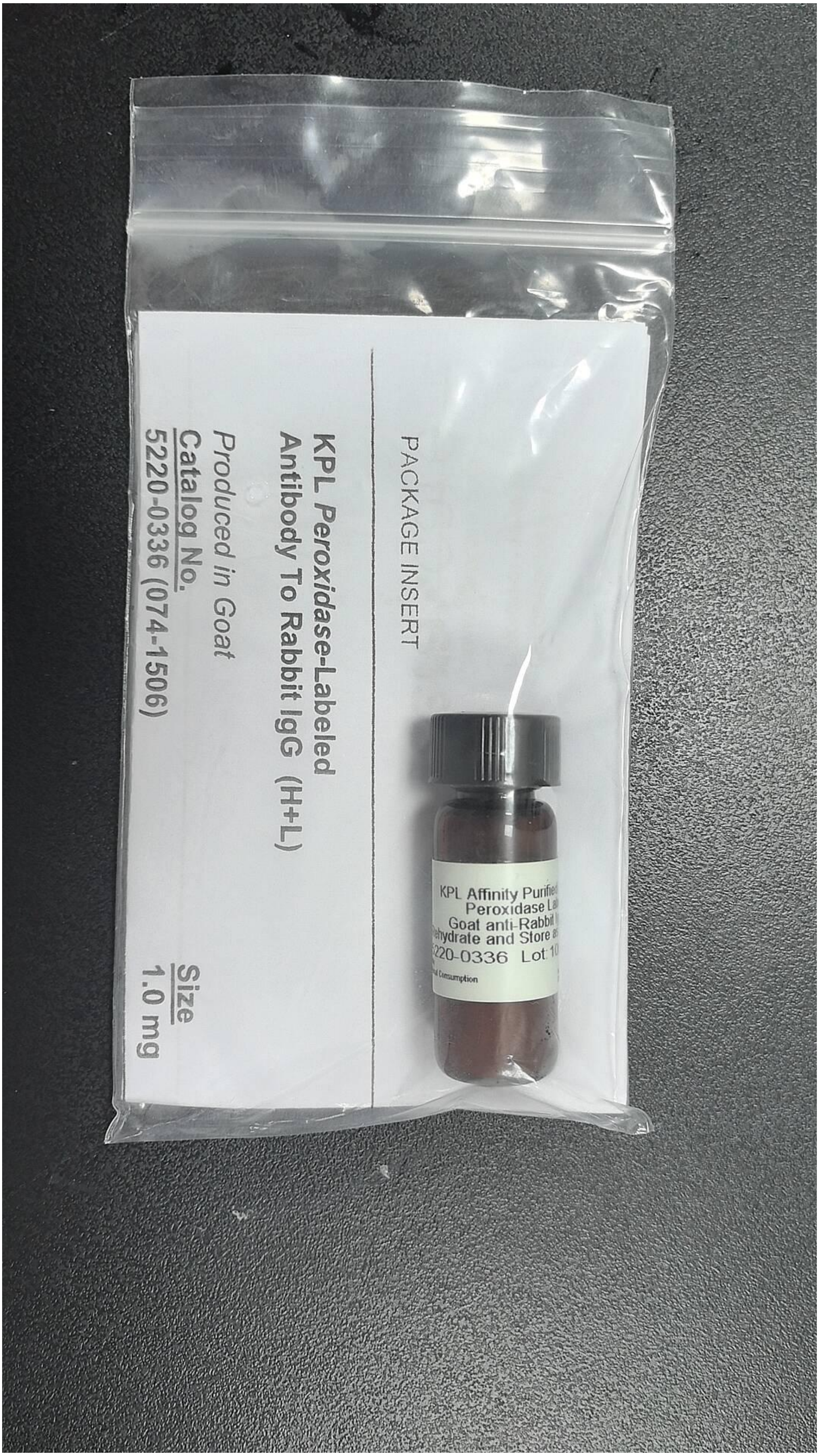
PACKAGE INSERT
KPL Peroxidase-Labeled
Antibody To Rabbit IgG (H+L)
Produced in Goat
Catalog No.
5220-0336 (074-1506)
Size
1.0 mg
KPL Affinity Purified
Goat anti-Rabbit
Rehydrate and Store
Consumption

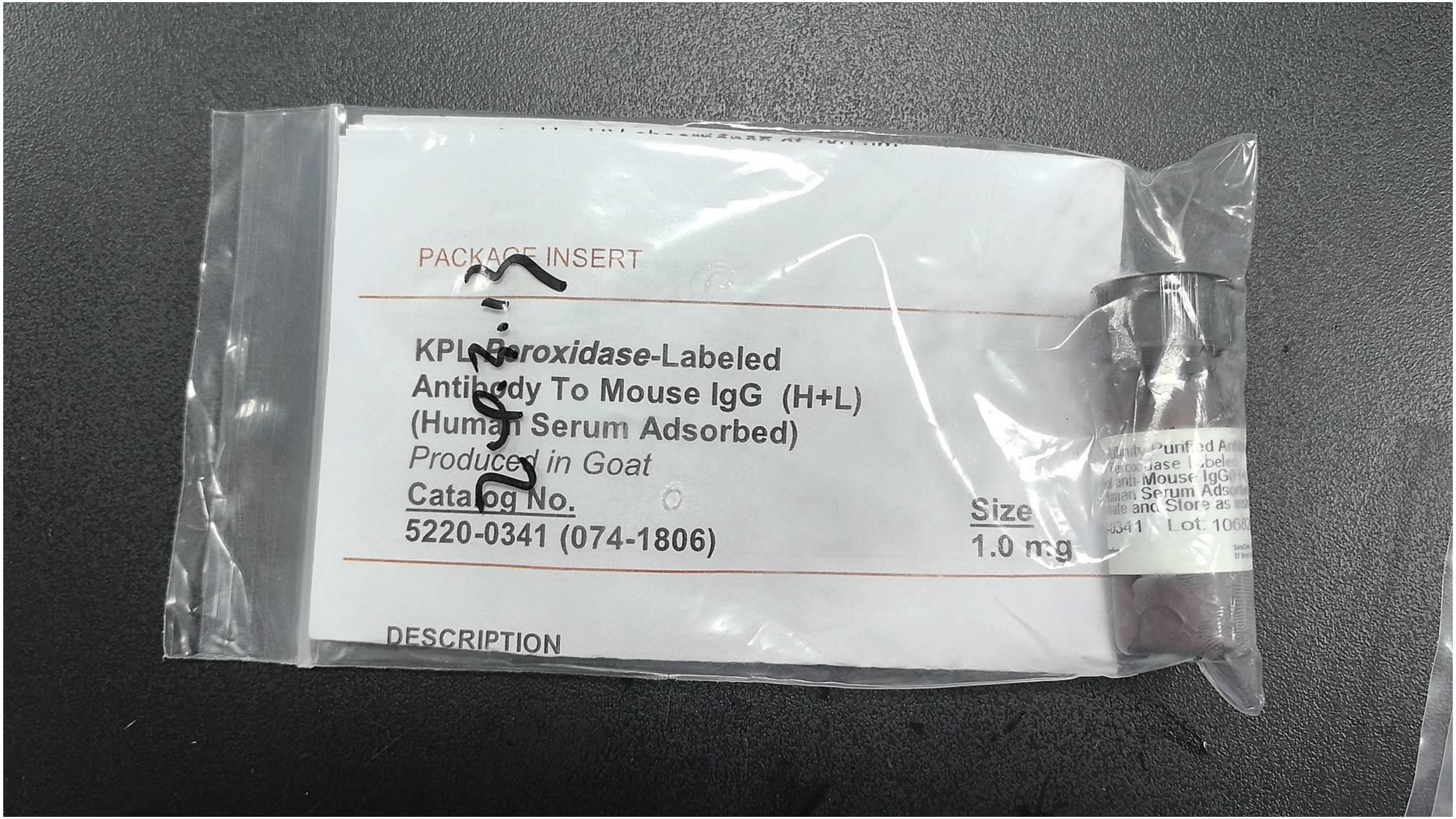
PACKAGE INSERT
KPL Peroxidase-Labeled
Antibody To Mouse IgG (H+L)
(Human Serum Adsorbed)
Produced in Goat
Catalog No.
5220-0341 (074-1806)
Size
1.0 mg
DESCRIPTION

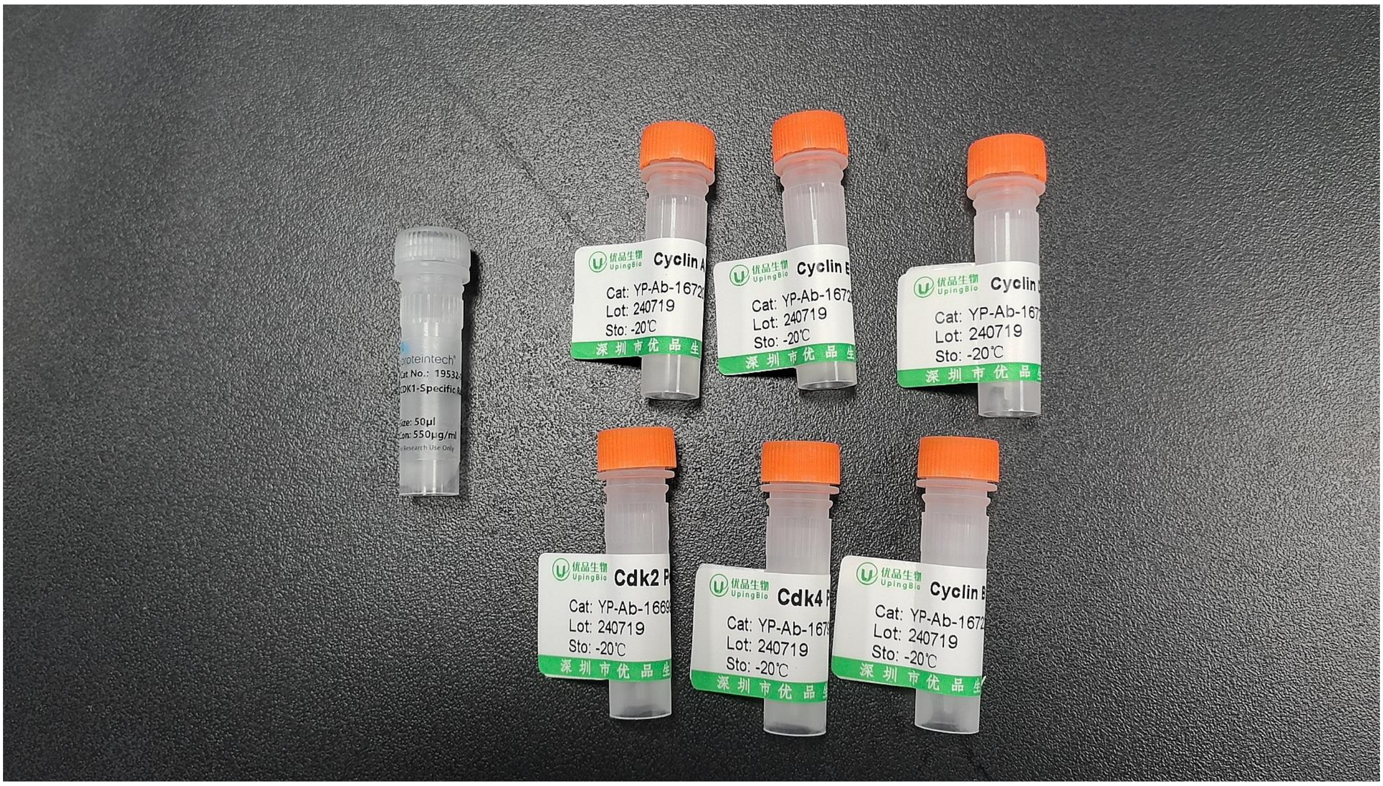
proteintech
Cat No.: 19532
DK1-Specific
Size: 50μl
550μg/ml
Research Use Only
优品生物
UpingBio
Cyclin A
Cat: YP-Ab-1672
Lot: 240719
Sto: -20℃
深 圳 市 优 品 生
Cyclin B
Cat: YP-Ab-1672
Lot: 240719
Sto: -20℃
深 圳 市 优 品 生
Cyclin
Cat: YP-Ab-167
Lot: 240719
Sto: -20℃
深 圳 市 优 品
Cdk2 P
Cat: YP-Ab-1669
Lot: 240719
Sto: -20℃
深 圳 市 优 品
Cdk4 P
Cat: YP-Ab-167
Lot: 240719
Sto: -20℃
深 圳 市 优 品
Cyclin B
Cat: YP-Ab-1672
Lot: 240719
Sto: -20℃
深 圳 市 优 品 生

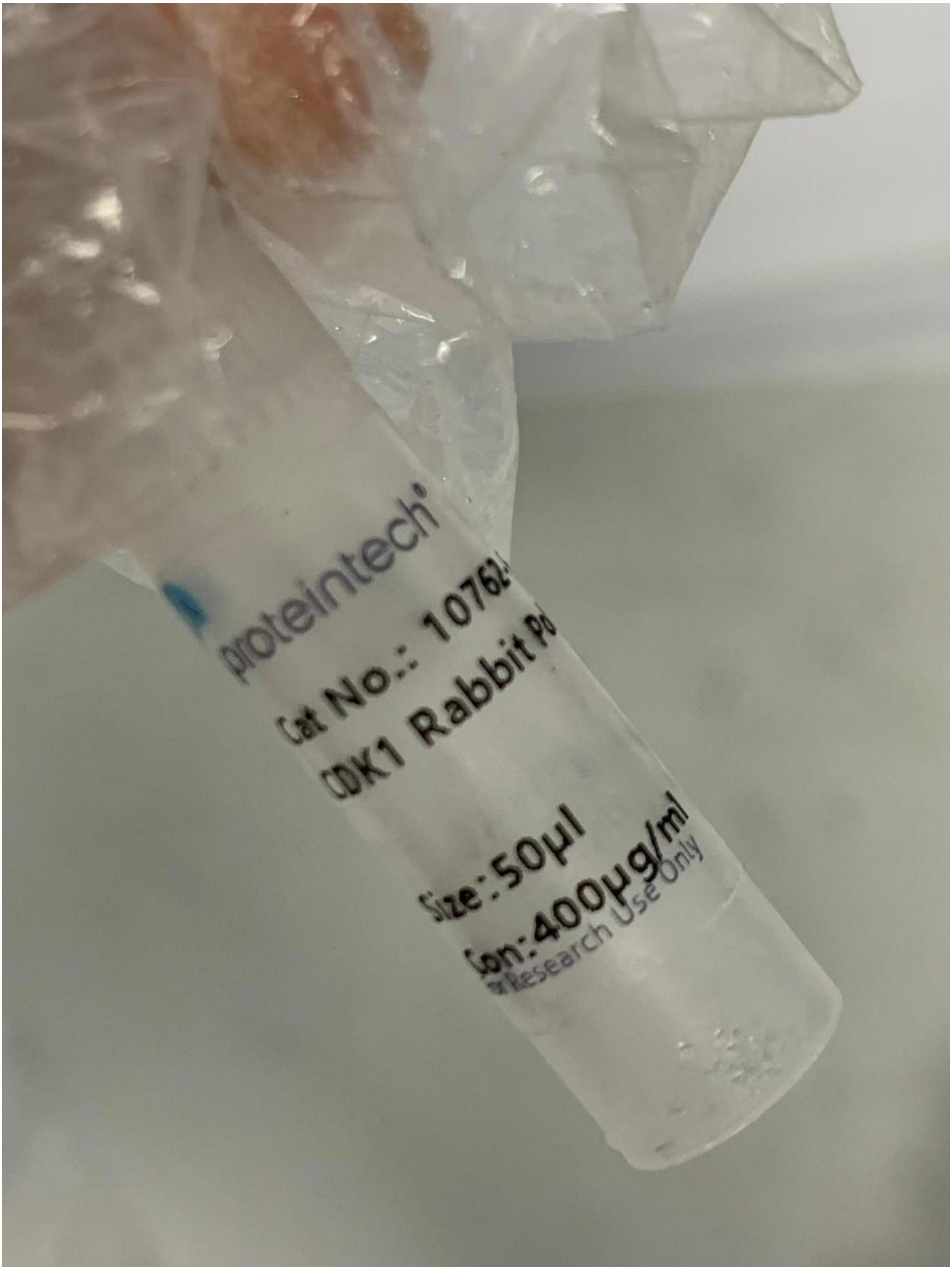
proteintech
Cat No.: 1076
CDK1 Rabbit Po
Size: 50μl
Con: 400μg/ml
Research Use Only

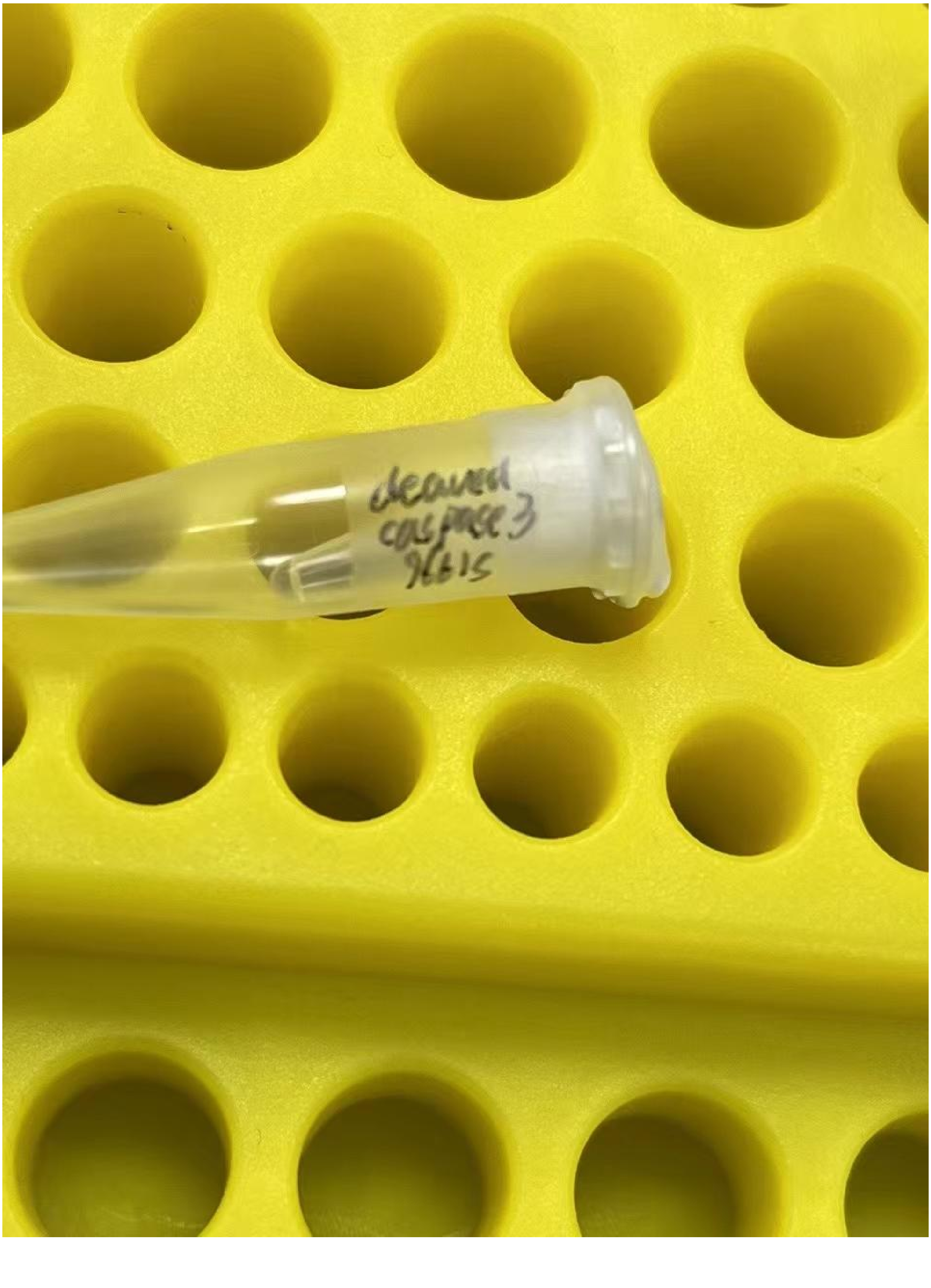
cleaved
caspase 3

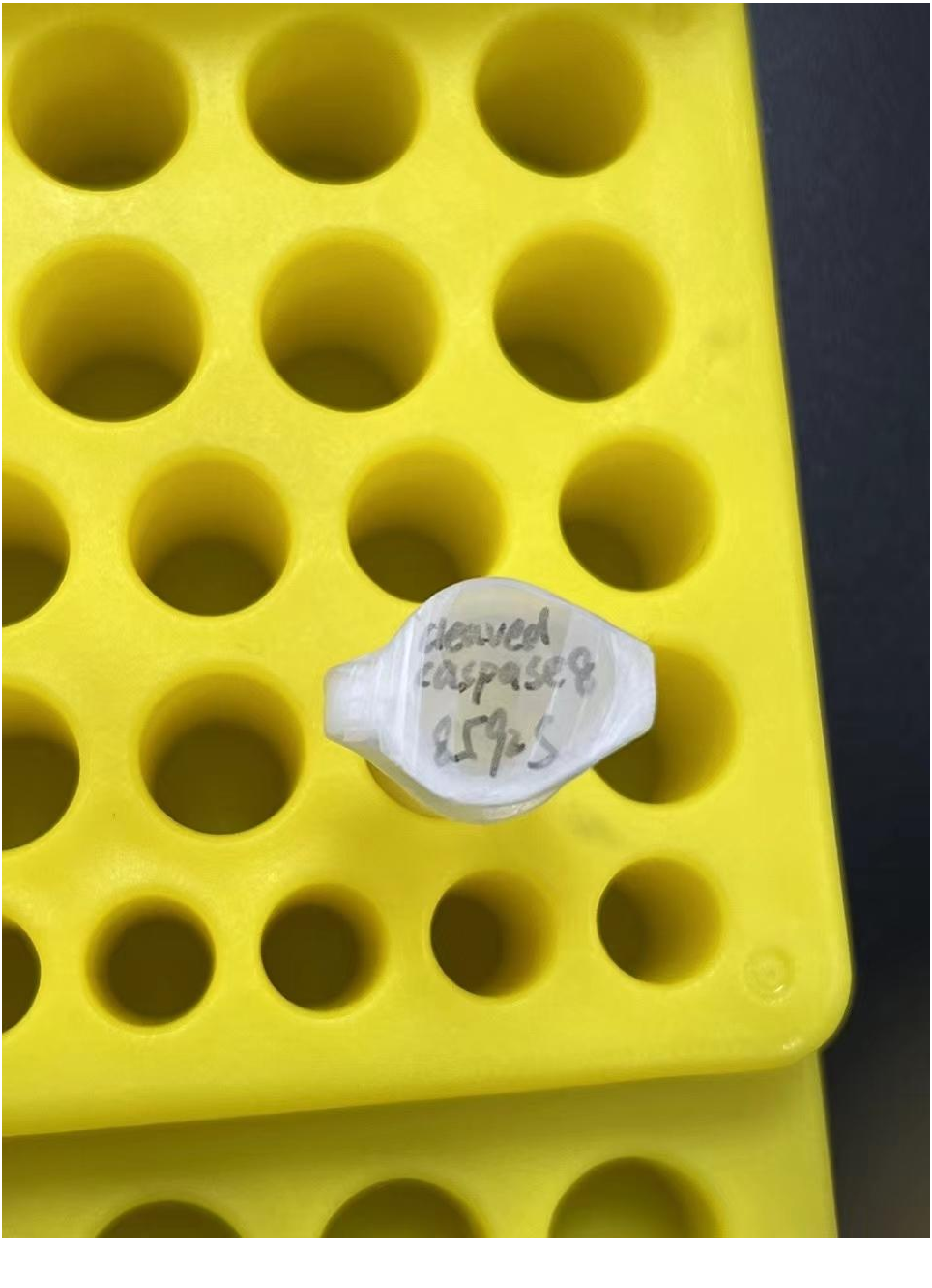
cleaved
caspase 8

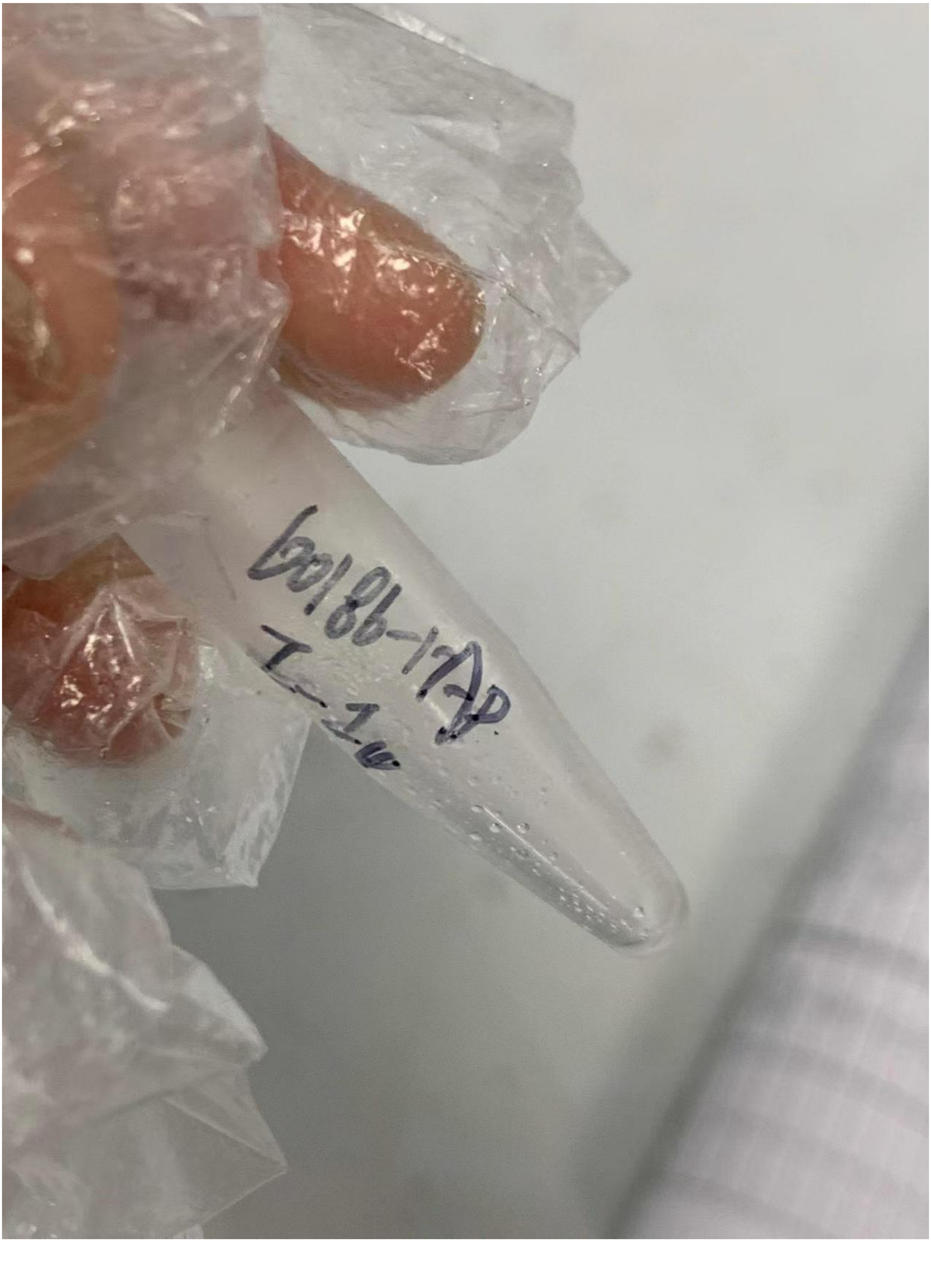

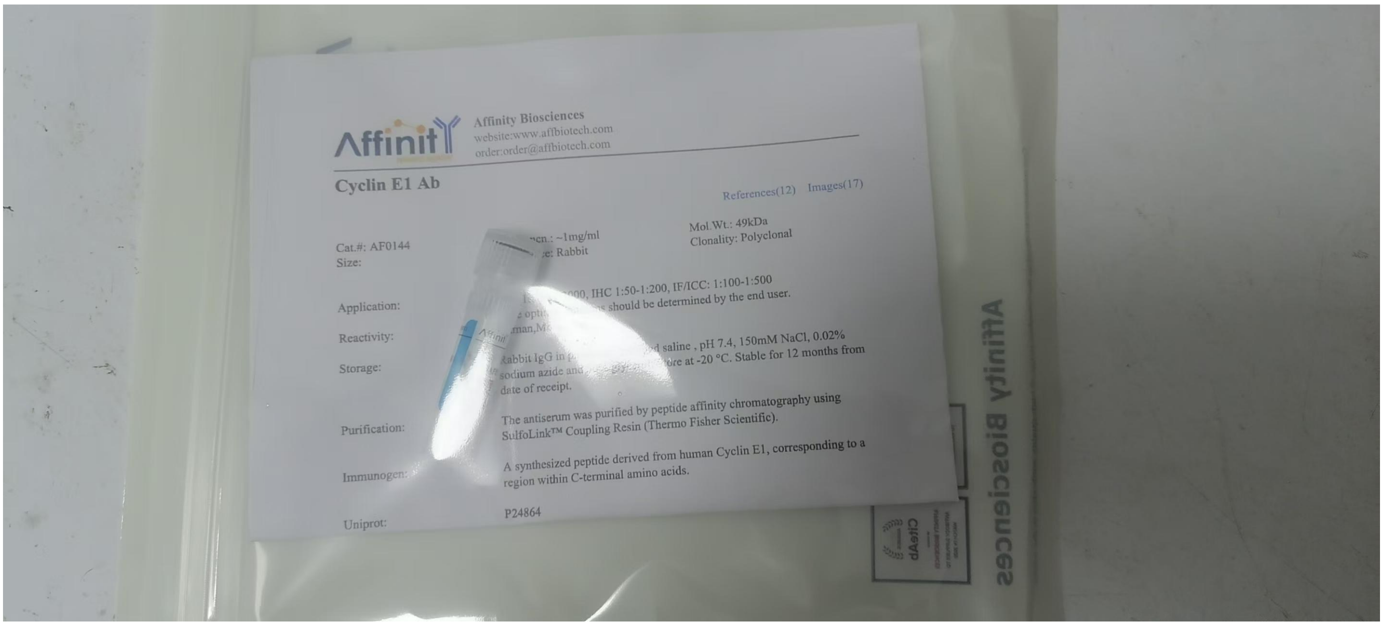

Affinity Biosciences
website:www.affbiotech.com
order:order@affbiotech.com
Cyclin E1 Ab
References(12)
Images(17)
Cat.#: AF0144
Size:
Mol.Wt.: 49kDa
Clonality: Polyclonal
Application:
IHC 1:50-1:200, IF/ICC: 1:100-1:500
should be determined by the end user.
Reactivity:
Storage:
saline , pH 7.4, 150mM NaCl, 0.02%
Stable for 12 months from
date of receipt.
Purification:
The antiserum was purified by peptide affinity chromatography using SulfoLink™ Coupling Resin (Thermo Fisher Scientific).
Immunogen:
A synthesized peptide derived from human Cyclin E1, corresponding to a region within C-terminal amino acids.
Uniprot:
P24864

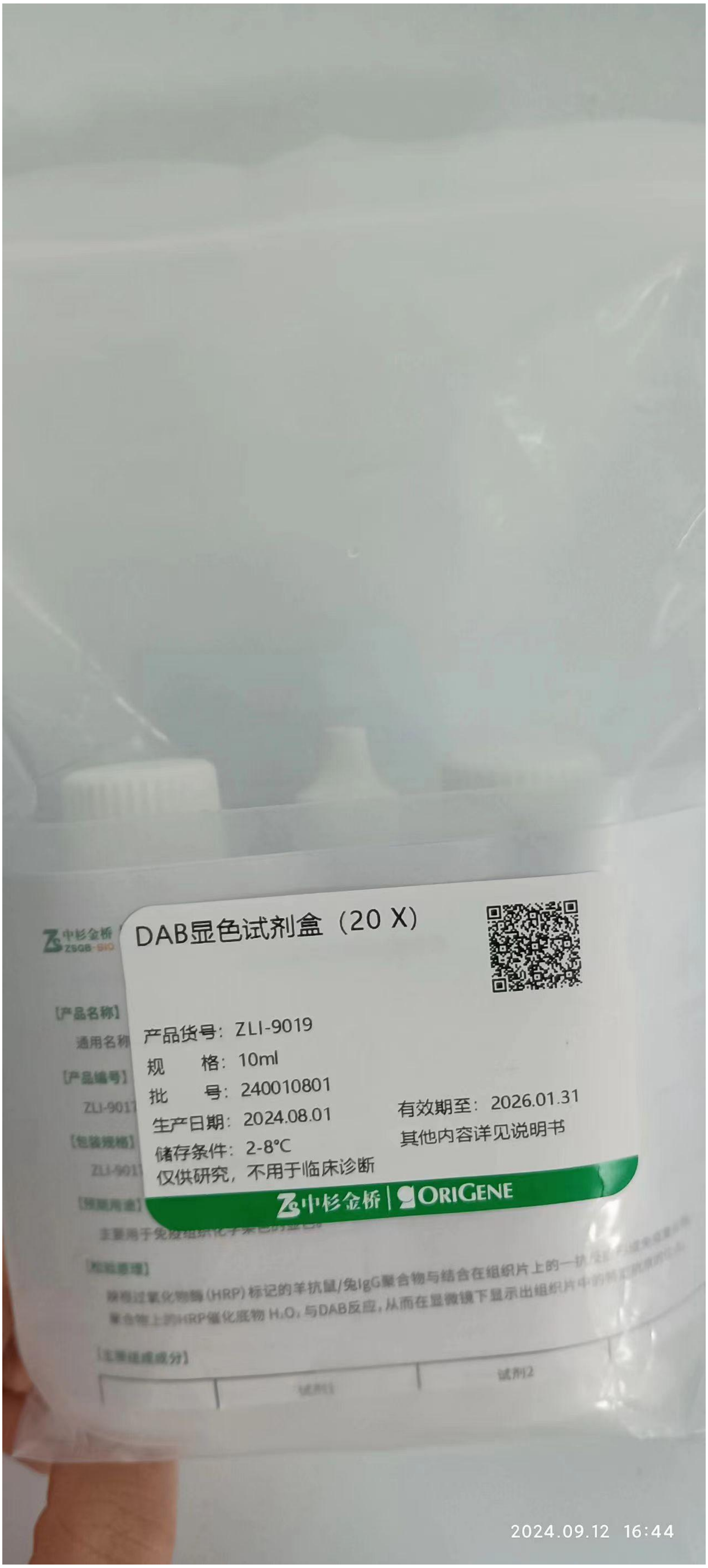
DAB显色试剂盒（20 X）
产品货号：ZLI-9019
规 格：10ml
批 号：240010801
生产日期：2024.08.01
储存条件：2-8℃
仅供研究，不用于临床诊断
有效期至：2026.01.31
其他内容详见说明书
中杉金桥 | ORIGENE
2024.09.12 16:44

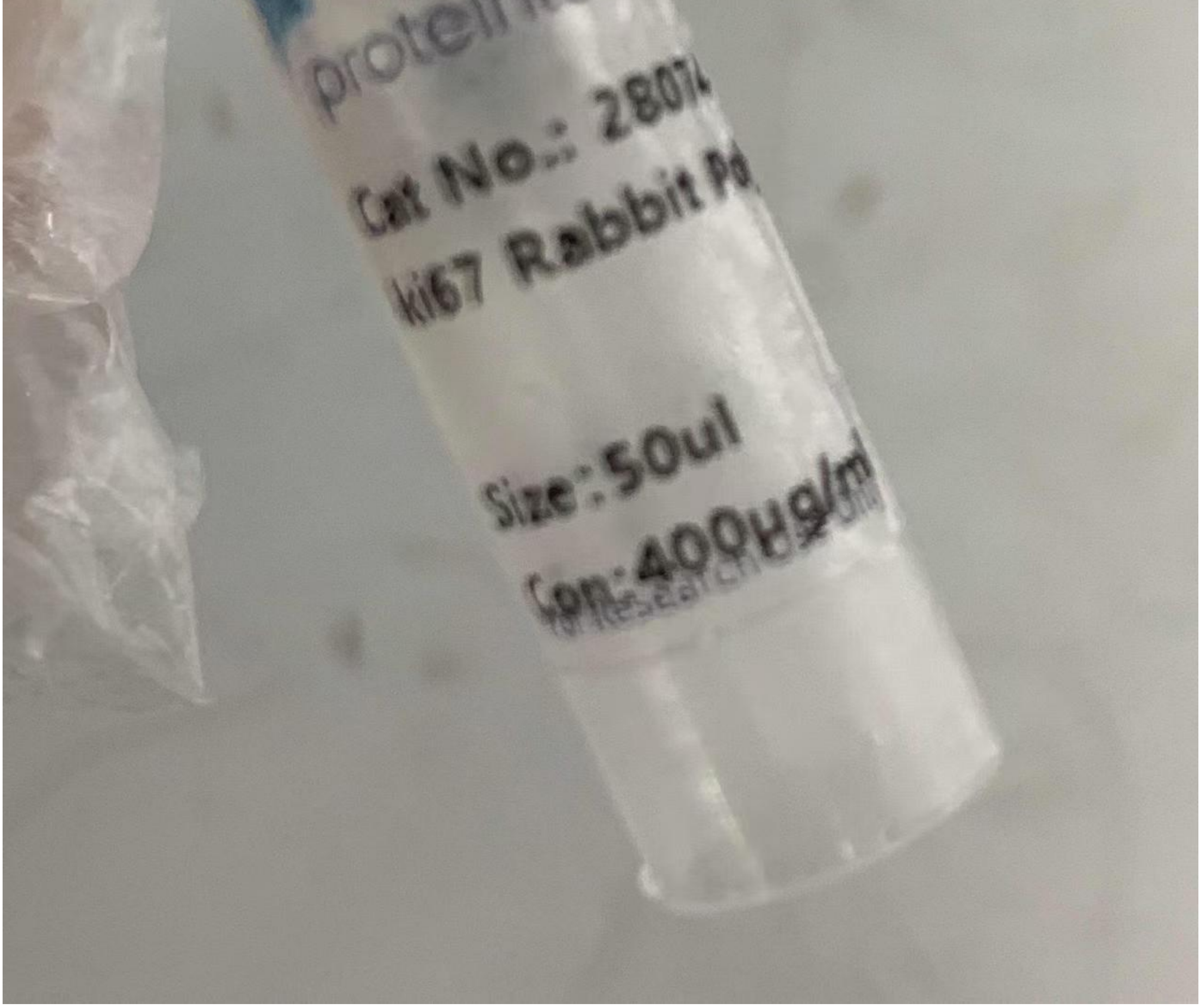
proteintech
Cat No.: 2807
Ki67 Rabbit Po
Size:50ul
Con:400μg/m

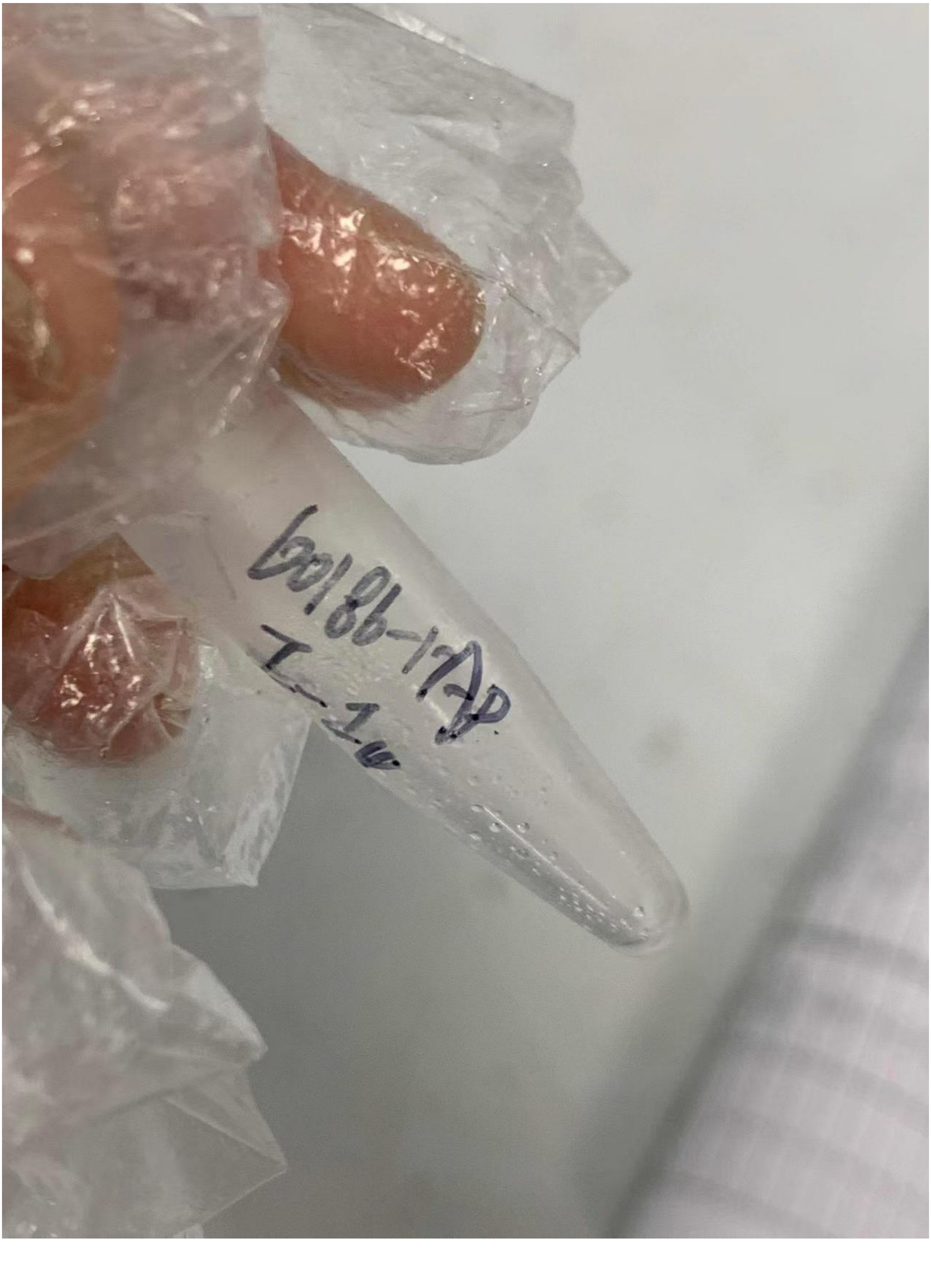

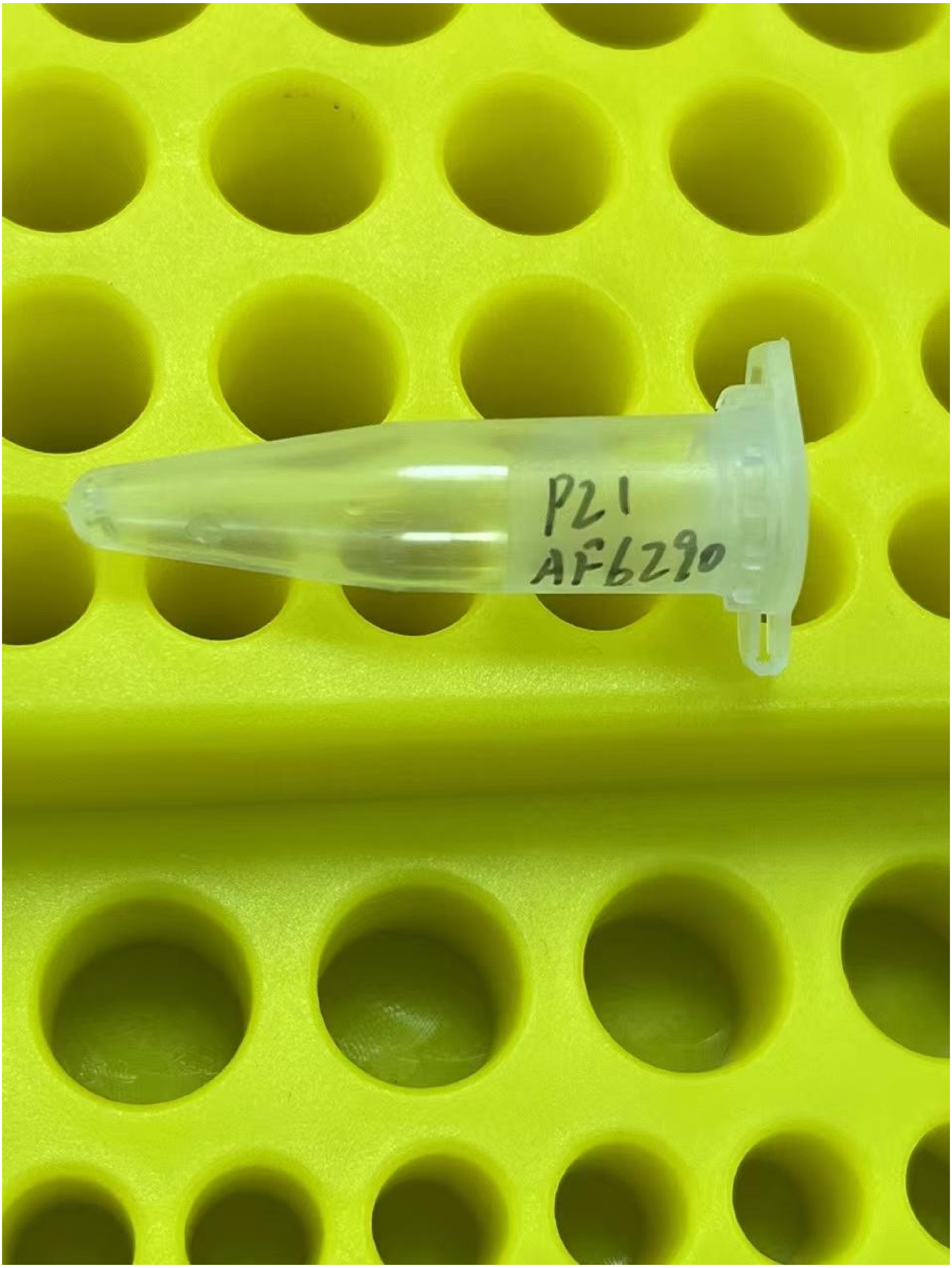
P21
AF6290

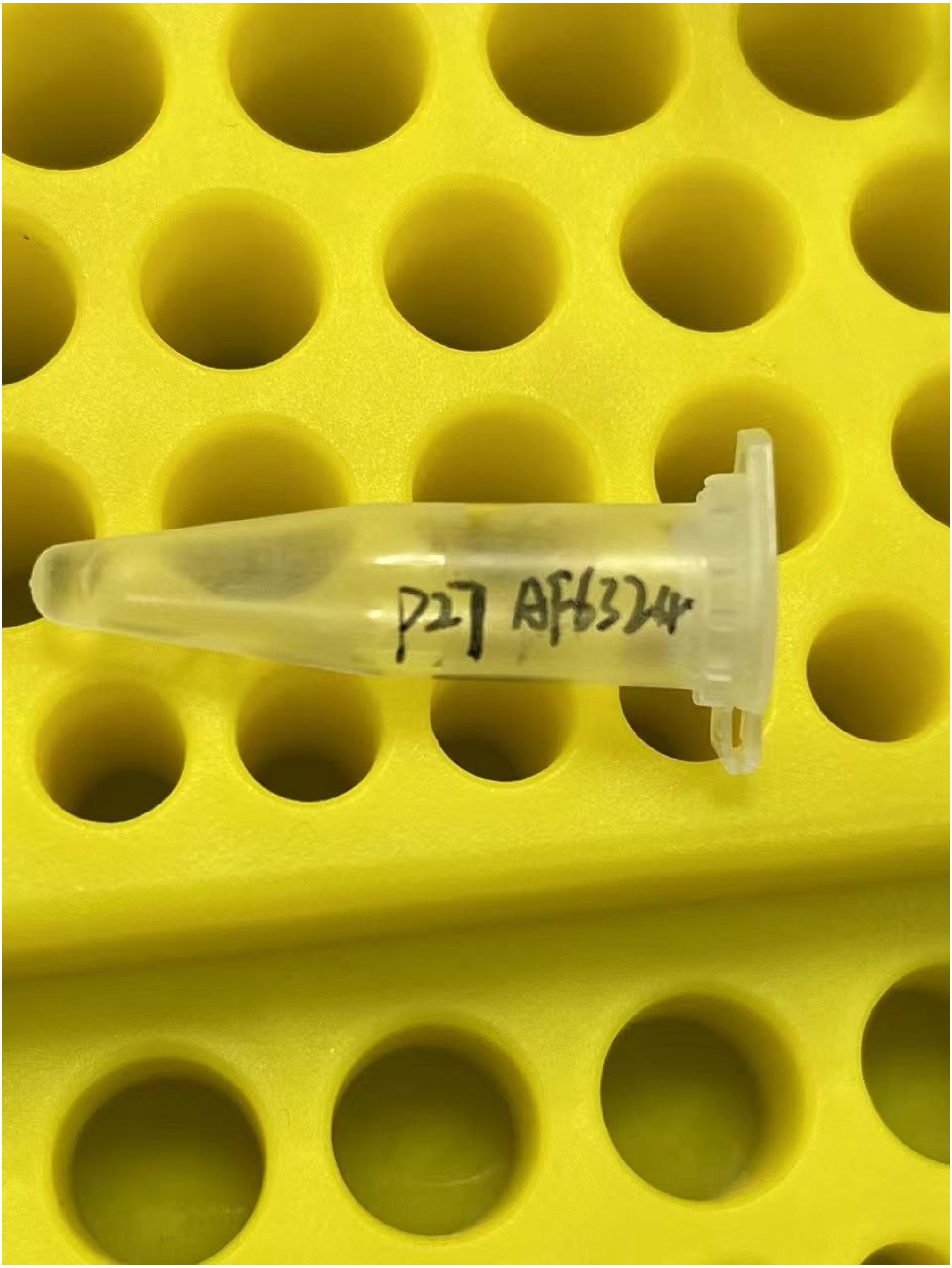
P27 AF6324

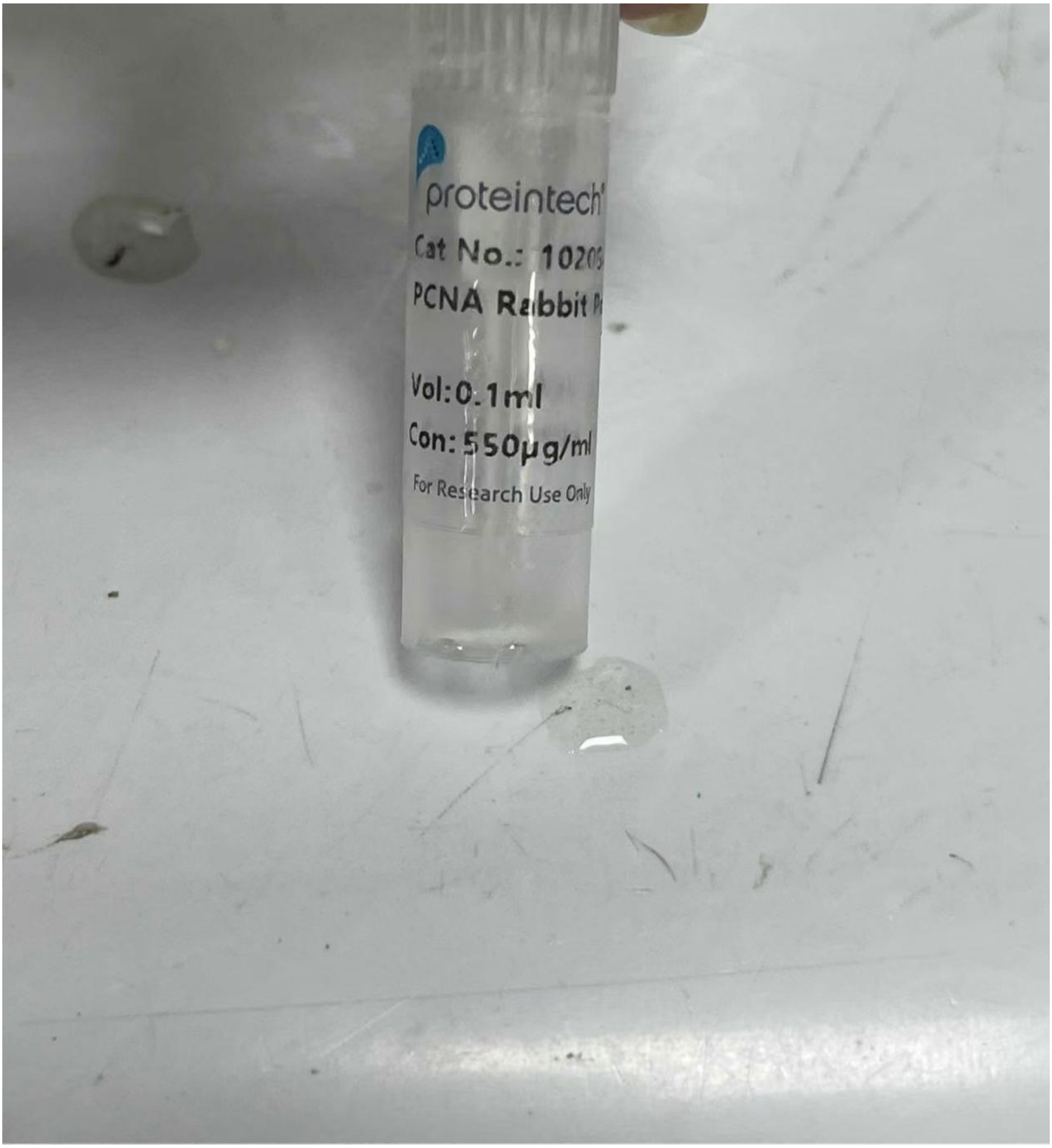
proteintech
Cat No.: 1020
PCNA Rabbit P
Vol:0.1ml
Con: 550μg/ml
For Research Use Only

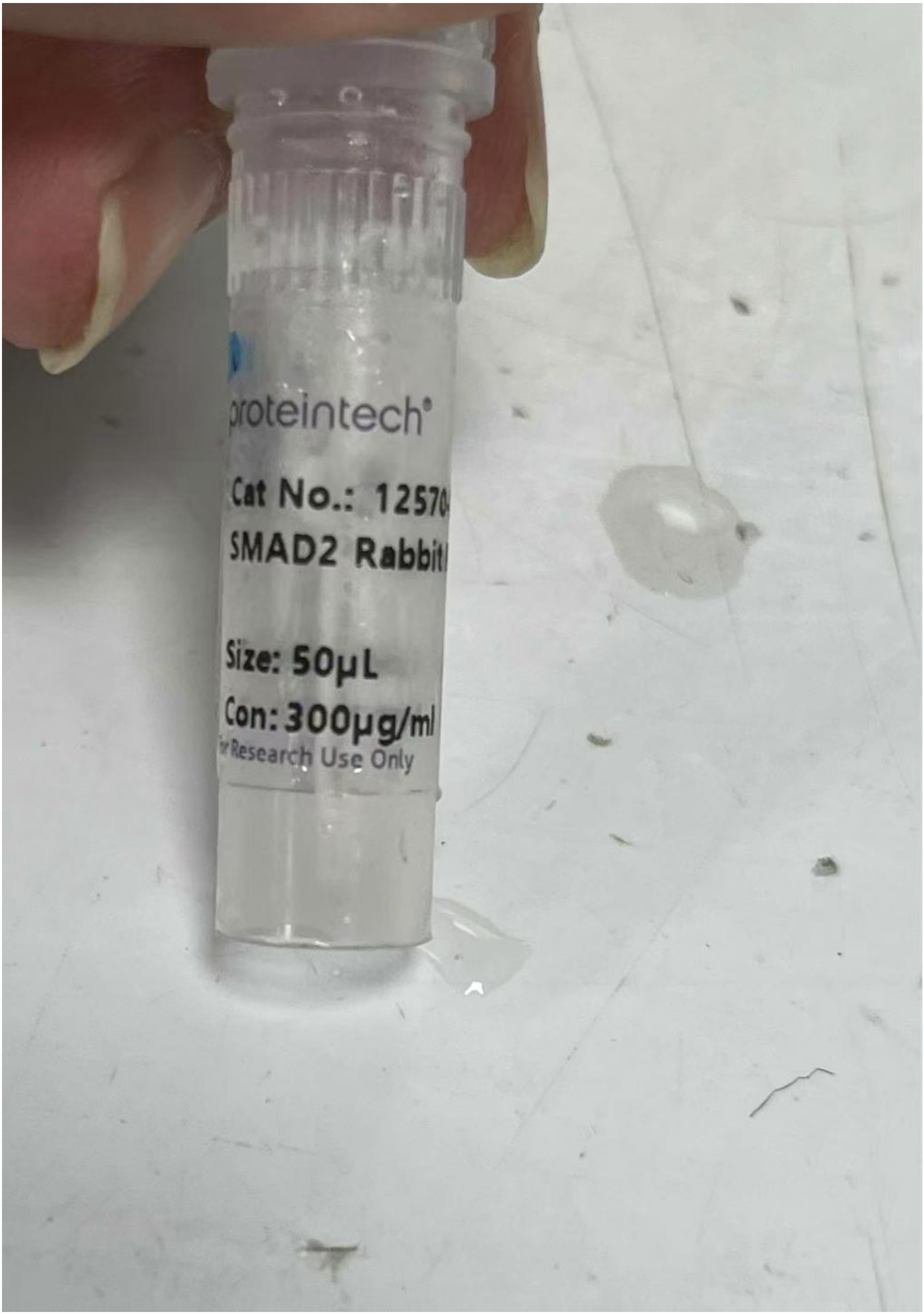
proteintech®
Cat No.: 12570
SMAD2 Rabbit
Size: 50μL
Con: 300μg/ml
Research Use Only

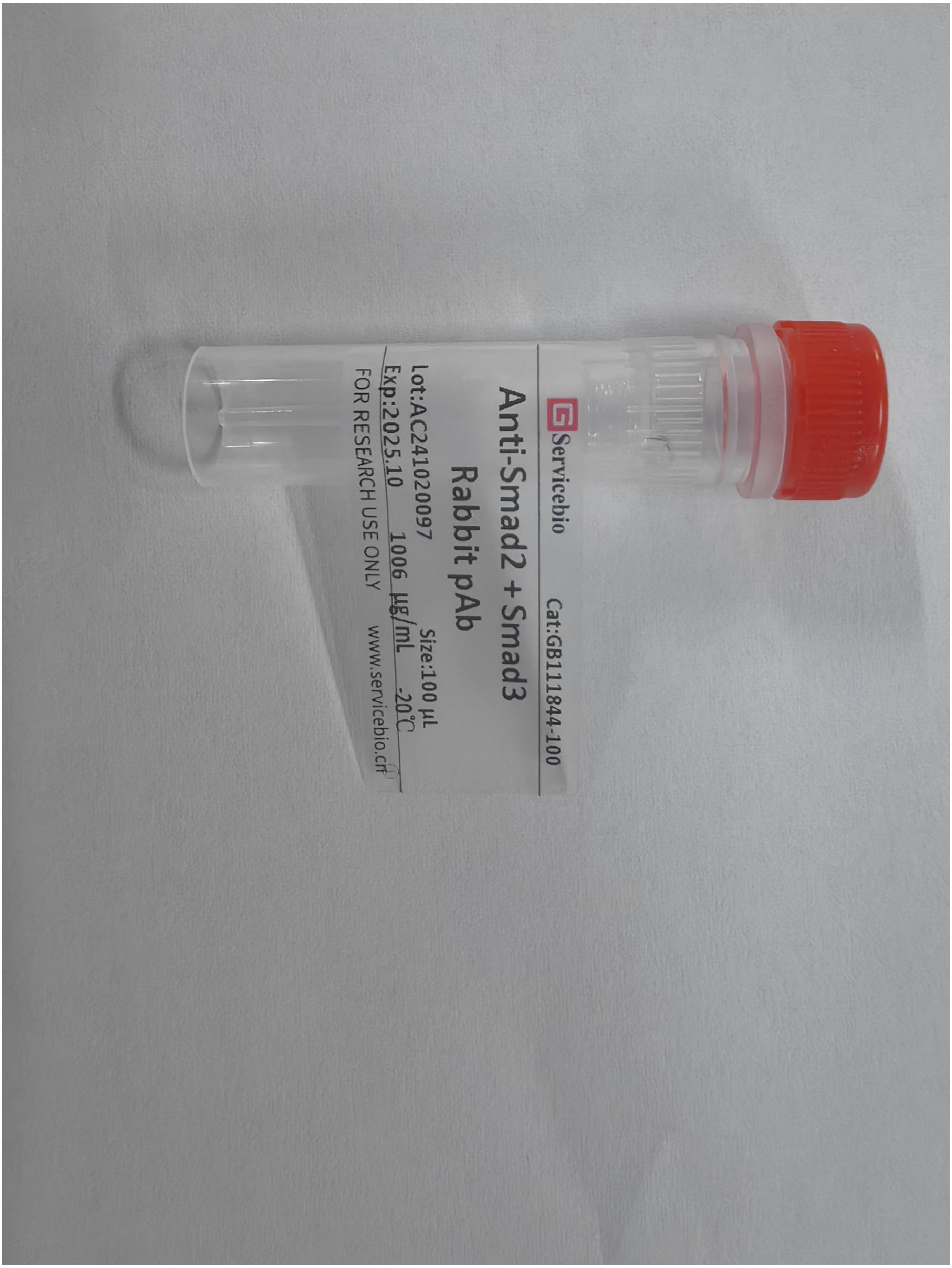
Servicebio
Cat:GB111844-100
Anti-Smad2 + Smad3
Rabbit pAb
Lot:AC241020097
Size:100 μL
Exp:2025.10
1006 μg/mL
-20°C
FOR RESEARCH USE ONLY
www.servicebio.cn

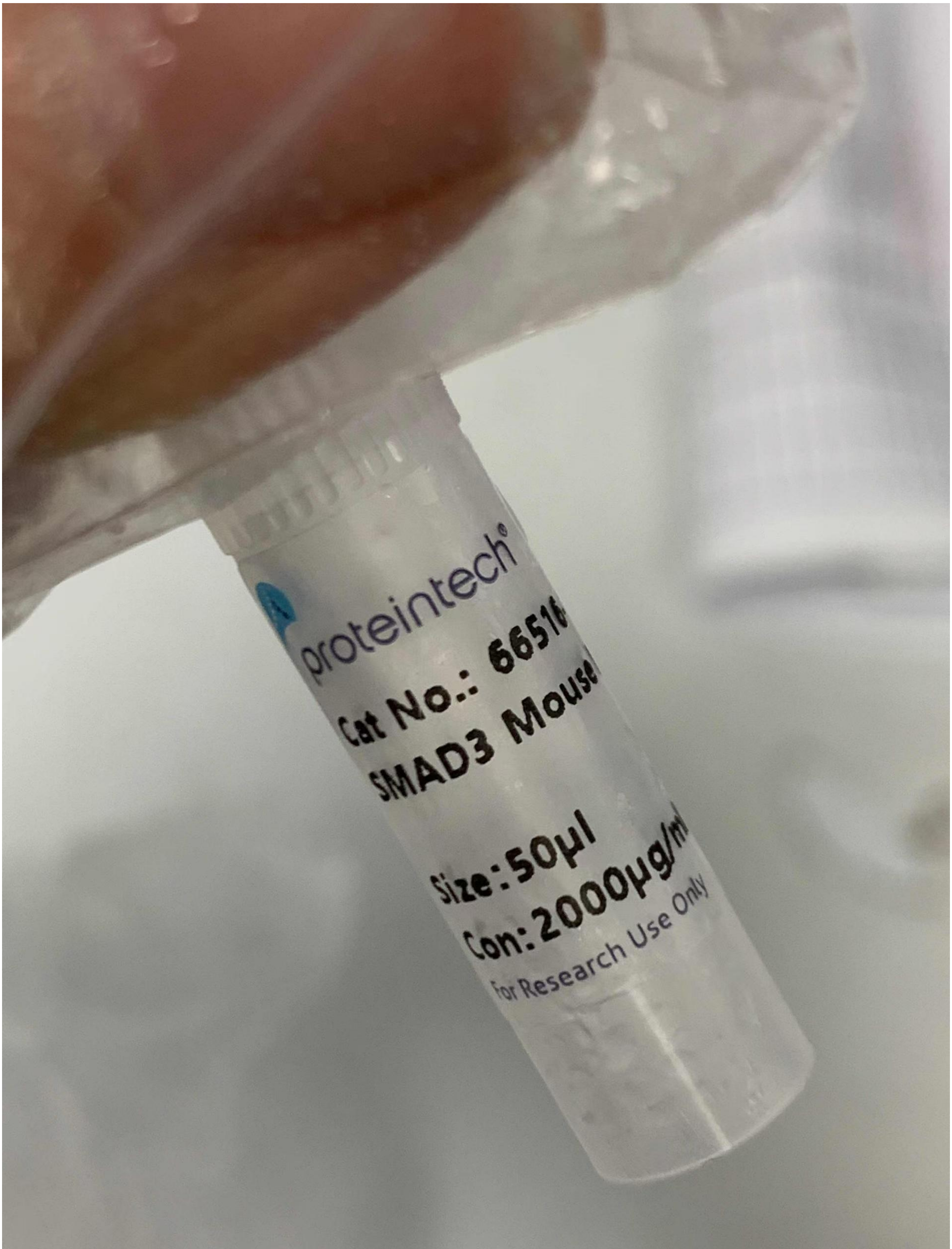
proteintech
Cat No.: 6651
SMAD3 Mouse
Size:50µl
Con:2000µg/m
For Research Use Only

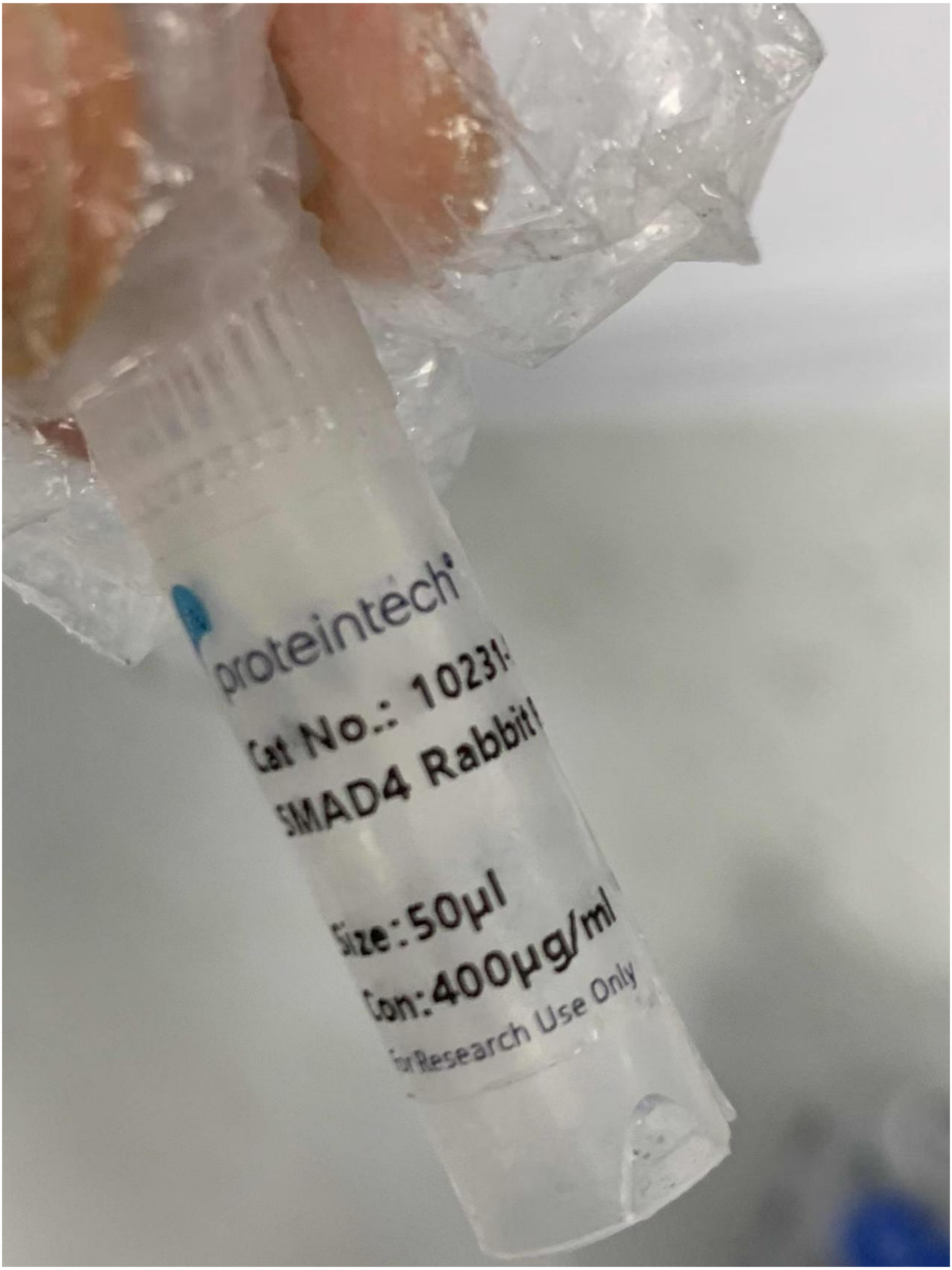
proteintech
Cat No.: 10231
SMAD4 Rabbit
Size:50µl
Con:400µg/ml
For Research Use Only

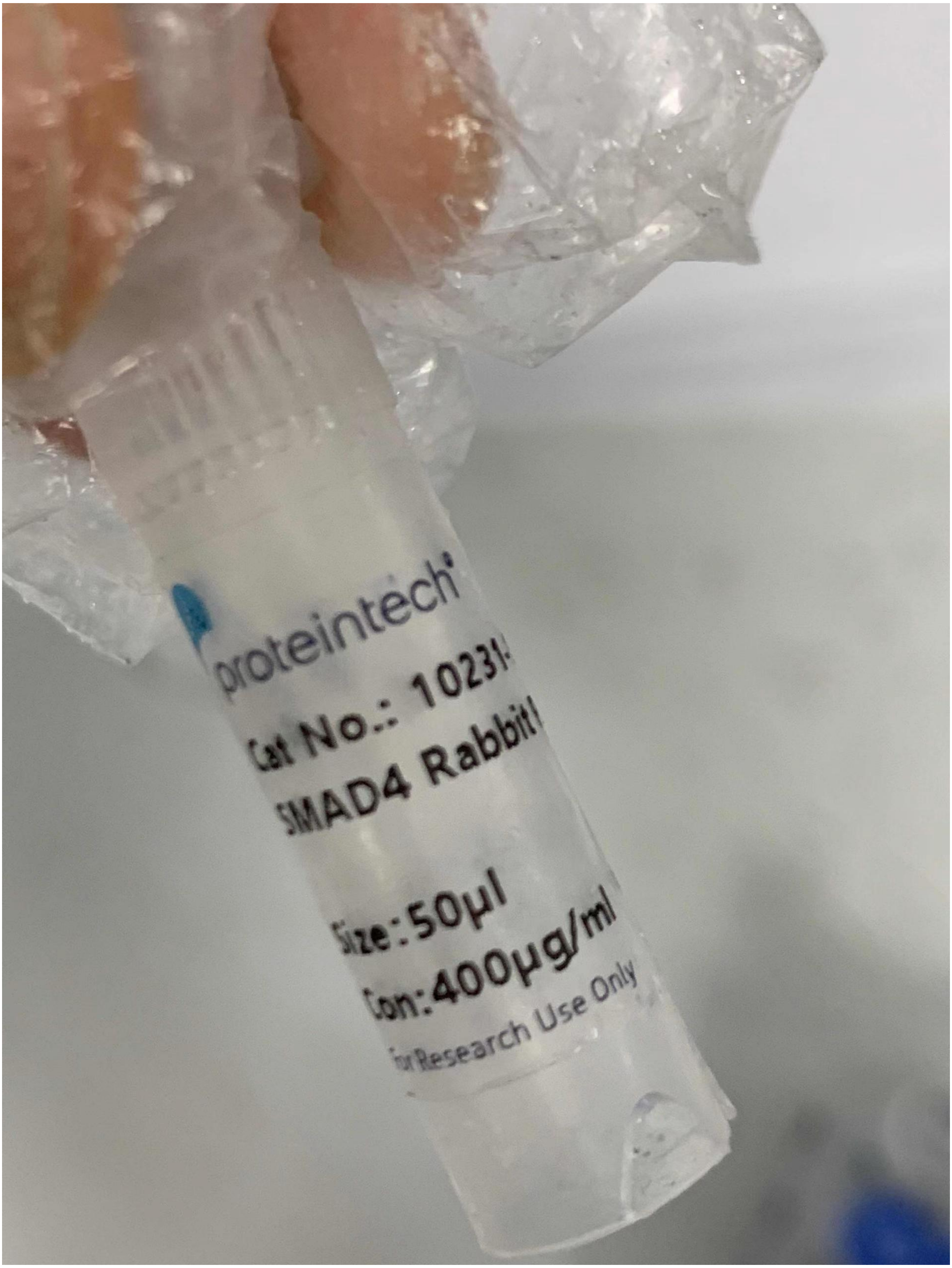
proteintech
Cat No.: 10231
SMAD4 Rabbit
Size:50µl
Con:400µg/ml
For Research Use Only

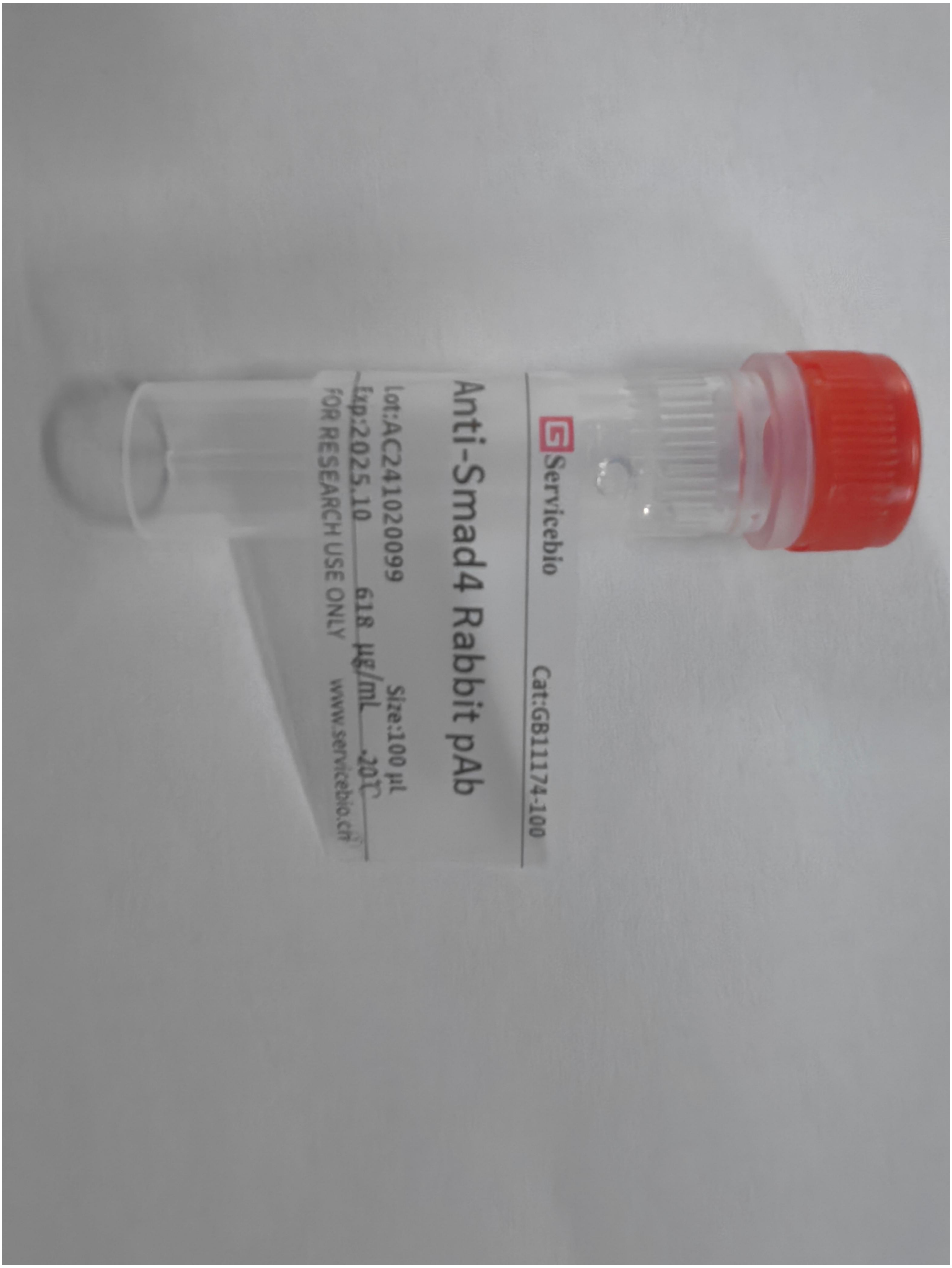
Servicebio
Cat:GB11174-100
Anti-Smad4 Rabbit pAb
Lot:AC241020099
Size:100 µl
Exp:2025.10
618 µg/mL
-20℃
FOR RESEARCH USE ONLY
www.servicebio.cn

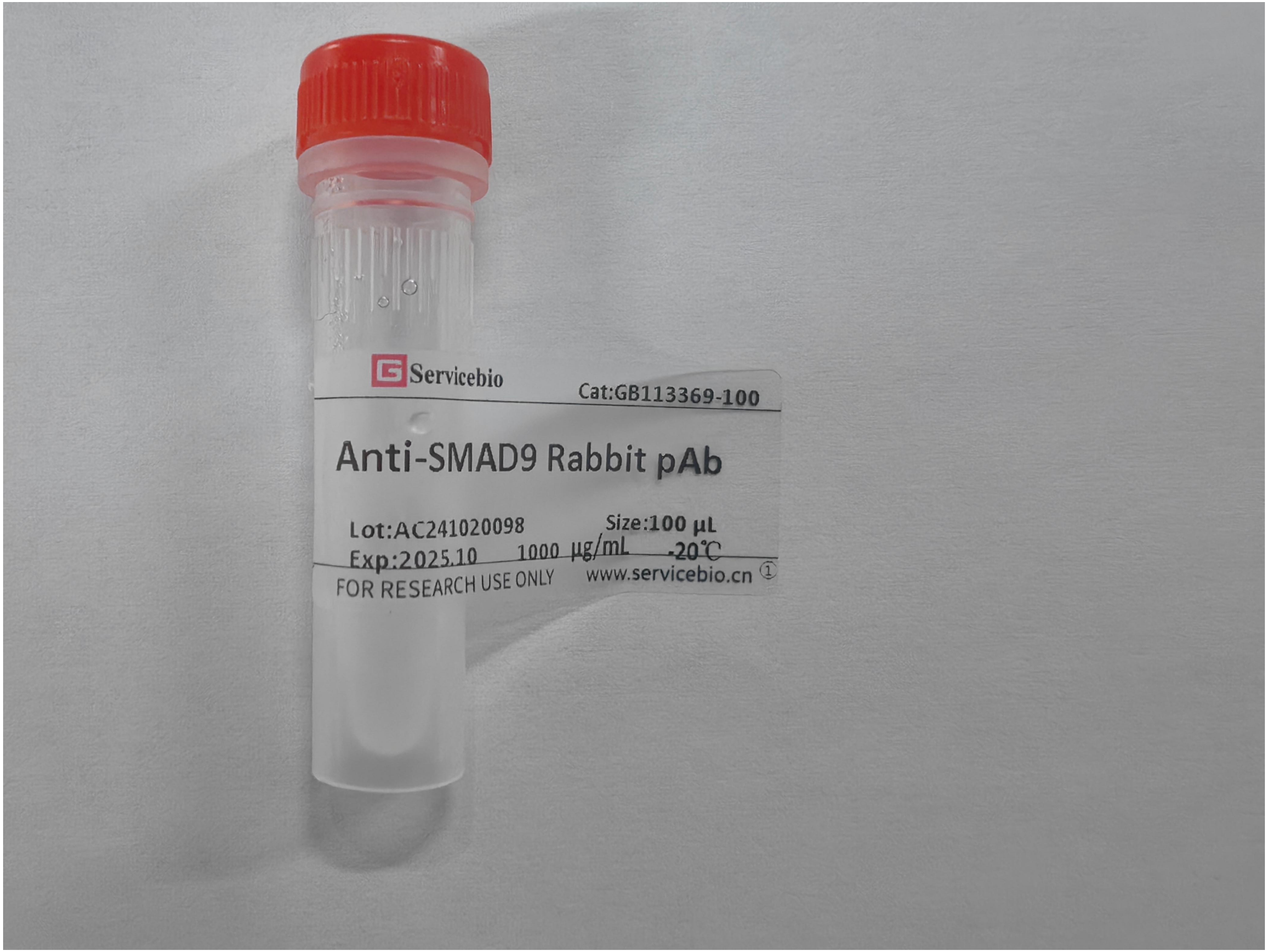
Servicebio
Cat:GB113369-100
Anti-SMAD9 Rabbit pAb
Lot:AC241020098
Size:100 μL
Exp:2025.10
1000 μg/mL
-20℃
FOR RESEARCH USE ONLY
www.servicebio.cn

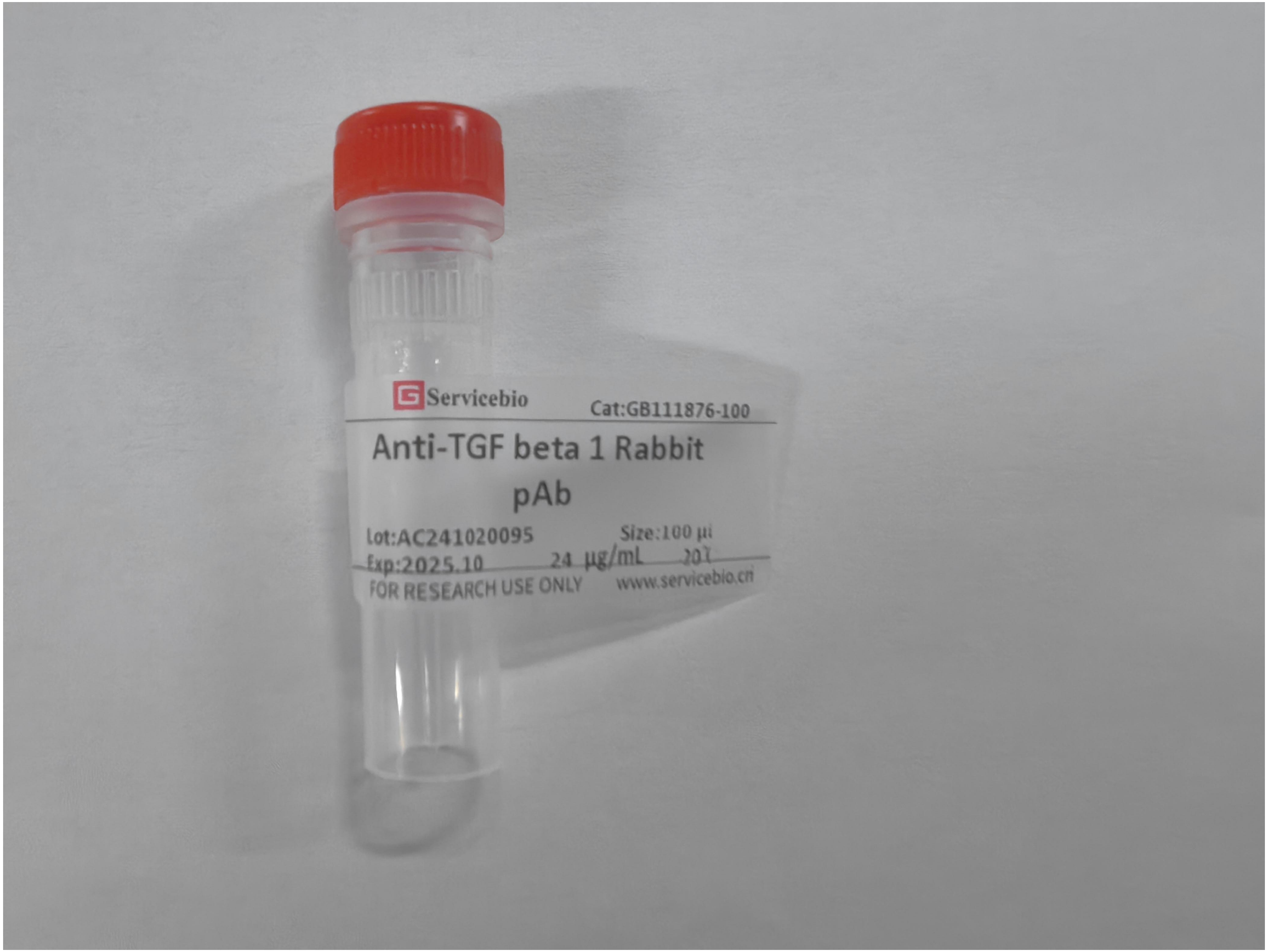
Servicebio
Cat:GB111876-100
Anti-TGF beta 1 Rabbit
pAb
Lot:AC241020095
Size:100 μl
Exp:2025.10
24 μg/mL
FOR RESEARCH USE ONLY
www.servicebio.cn
