# Supplementary material for: Lathyrol inhibits the proliferation of Renca cells by altering expression of TGF-β/Smad pathway components and subsequently affecting the cell cycle
Source: Front Oncol. 2025 Oct 8;15:1629962. doi: 10.3389/fonc.2025.1629962 (PMC12541422; doi:10.3389/fonc.2025.1629962)
Supplement: Supplementary file 11 [file Supplementaryfile2.pdf]

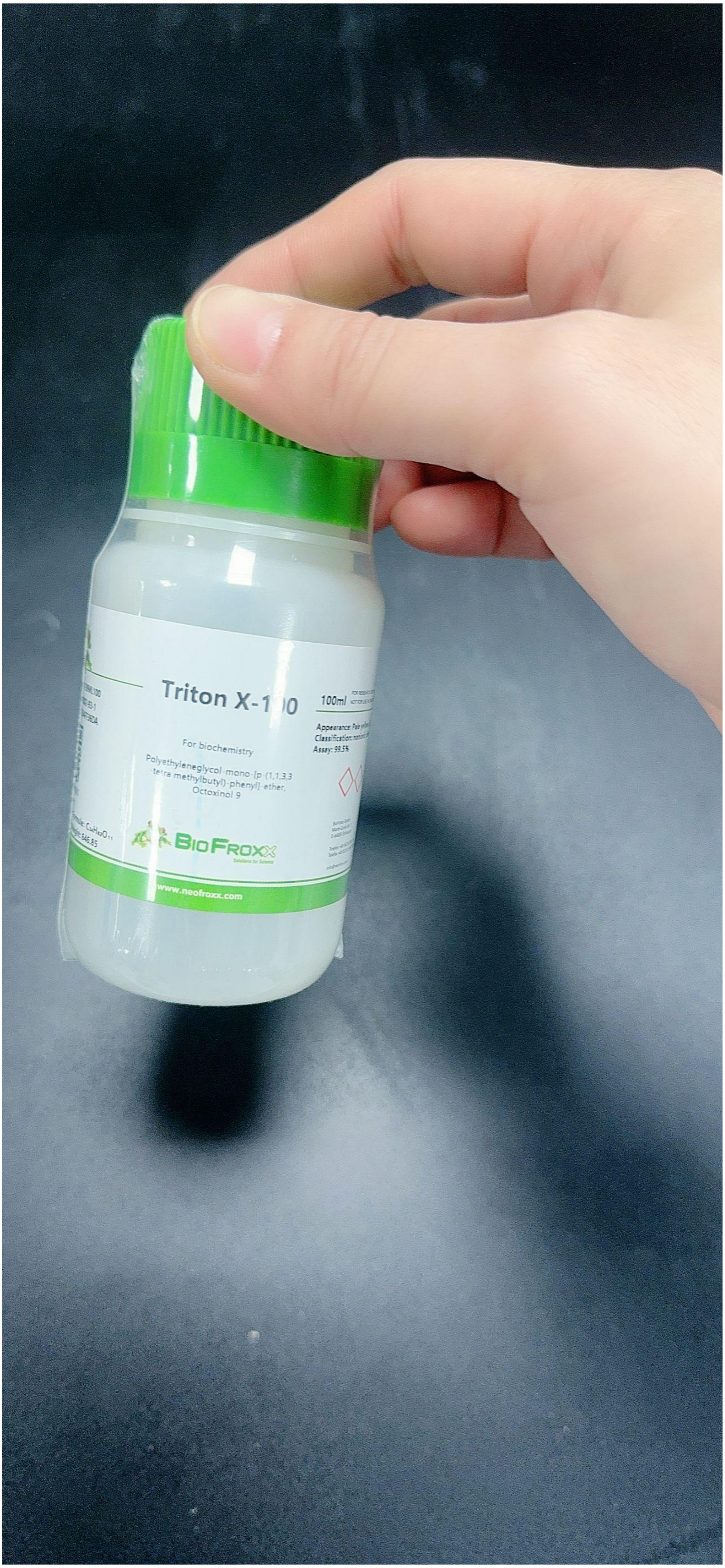
Triton X-1 0
100ml
For biochemistry
Polyethyleneglycol-mono-[p-(1,1,3,3
-tetra methylbutyl)-phenyl]-ether,
Octoxinol 9
Assay: 99.5%
BioFroxx
www.neofroxx.com

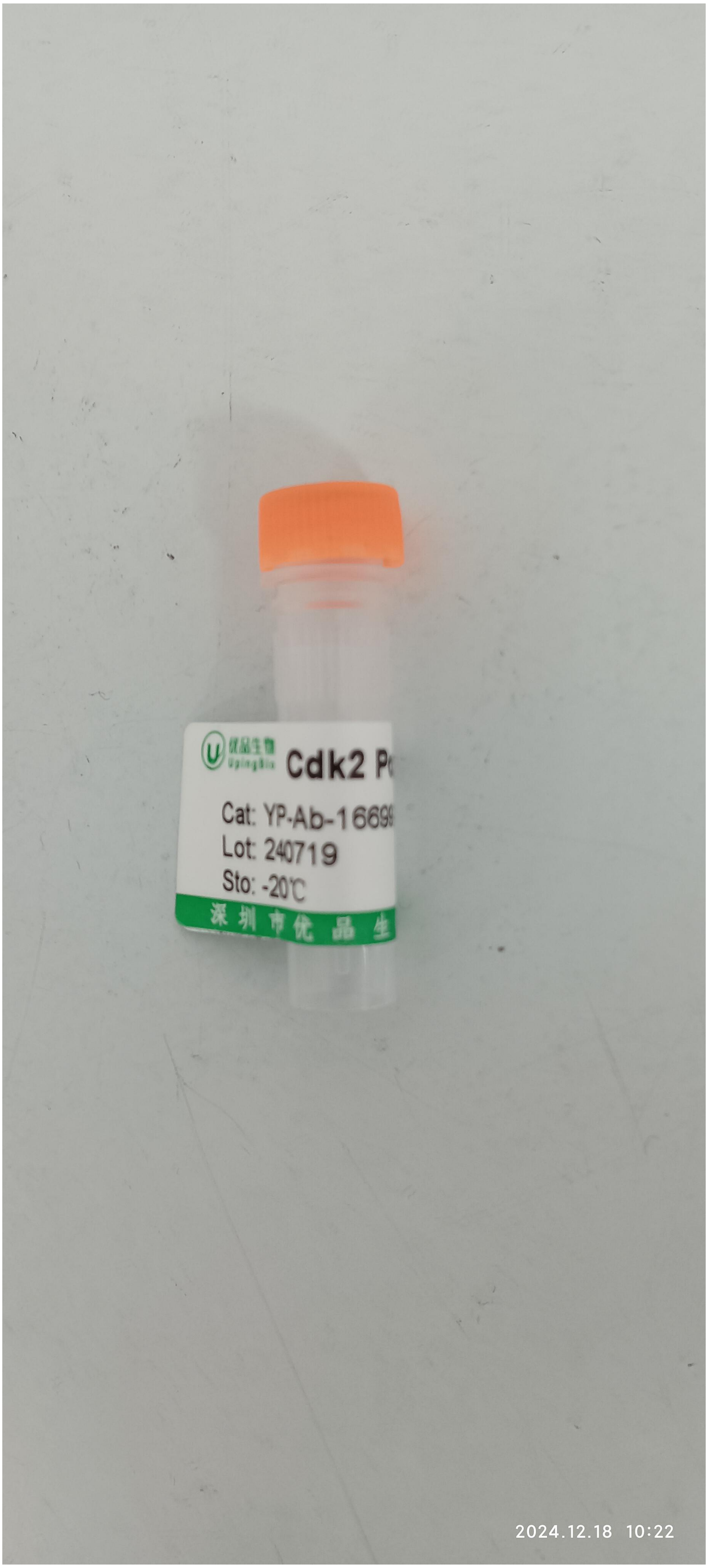
优品生物
UpingBio
Cdk2 Po
Cat: YP-Ab-1669
Lot: 240719
Sto: -20℃
深圳市优品生
2024.12.18 10:22

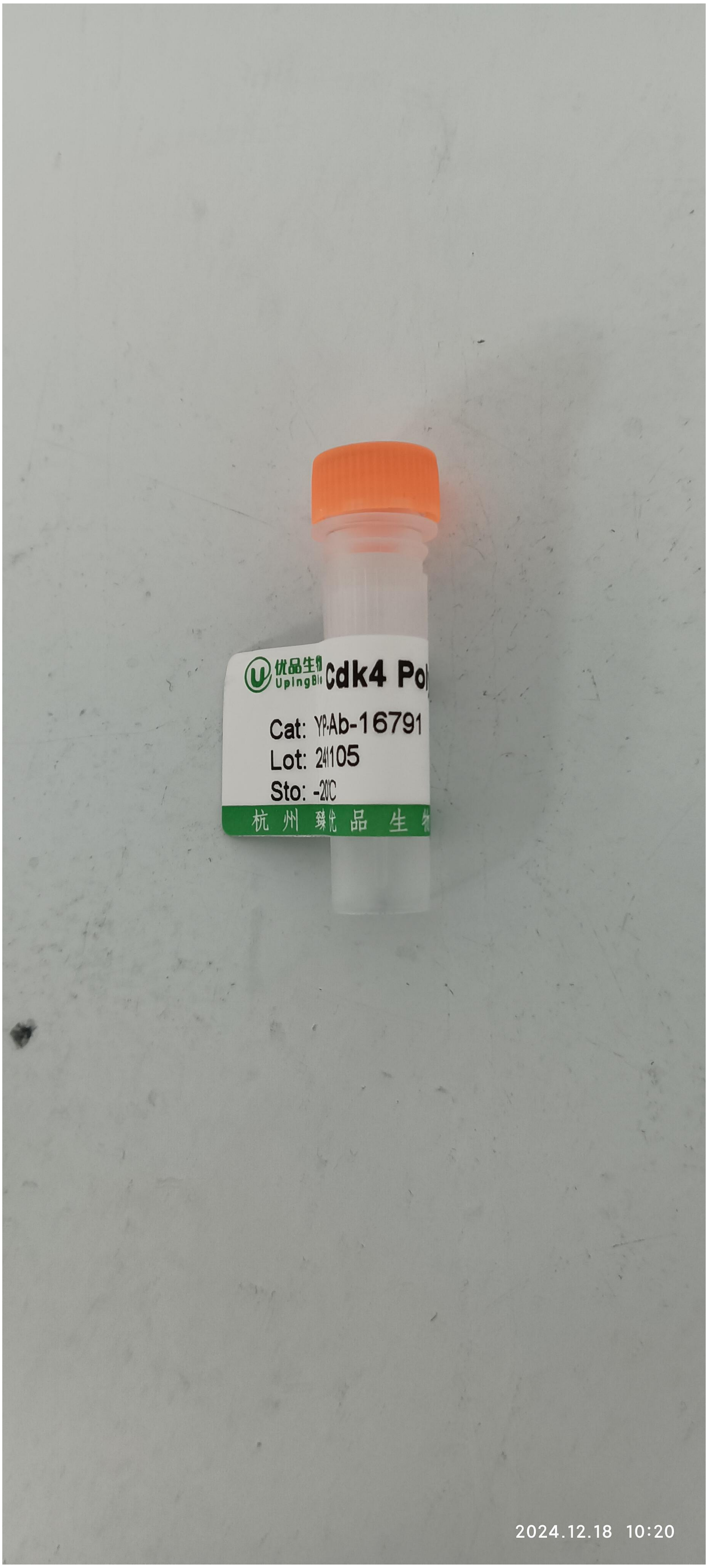
优品生物
Cdk4 Po
Cat: YP-Ab-16791
Lot: 241105
Sto: -20℃
杭 州 臻优 品 生
2024.12.18 10:20

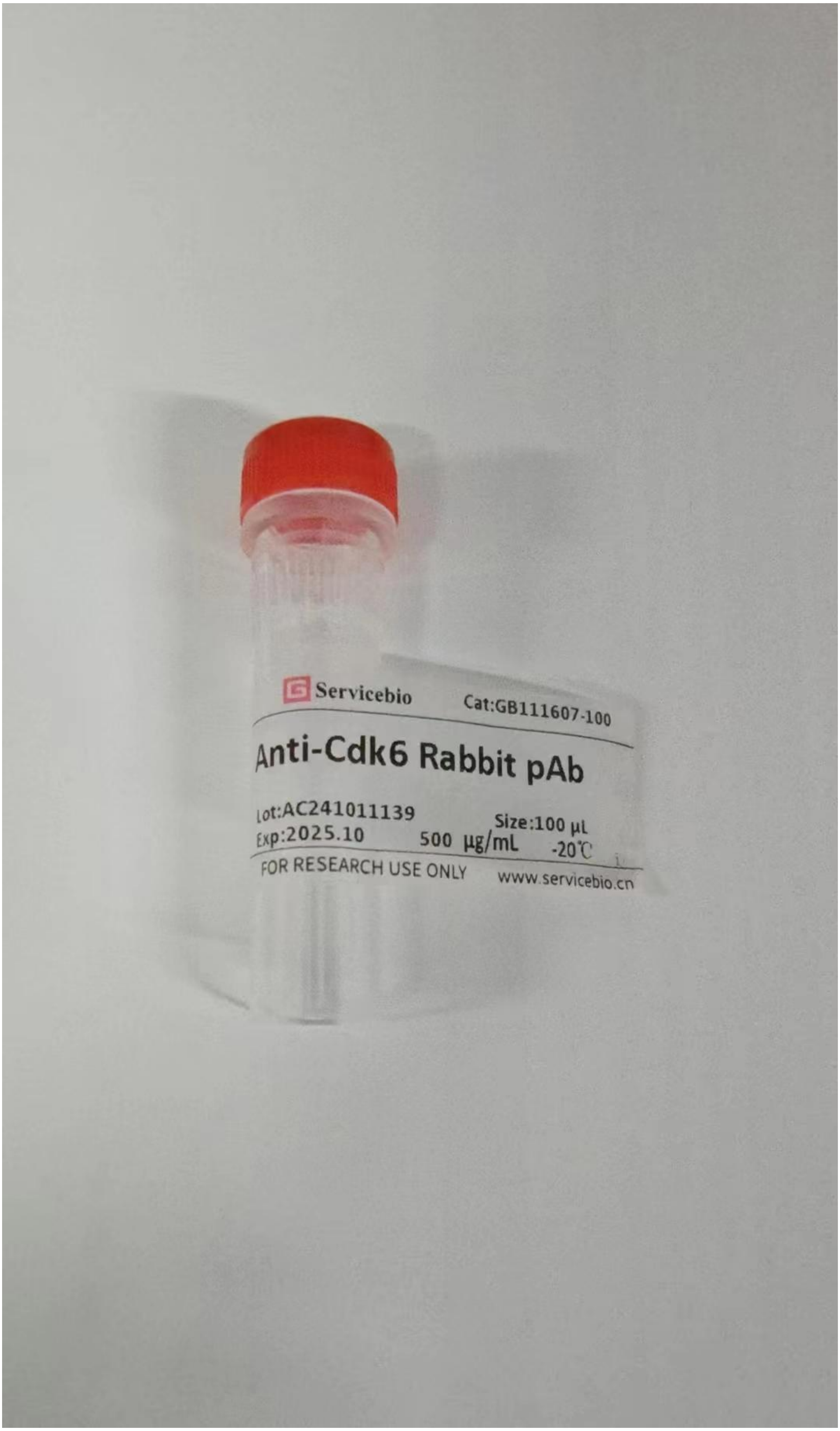
Servicebio
Cat:GB111607-100
Anti-Cdk6 Rabbit pAb
Lot:AC241011139
Size:100 μL
Exp:2025.10
500 μg/mL
-20℃
FOR RESEARCH USE ONLY
www.servicebio.cn

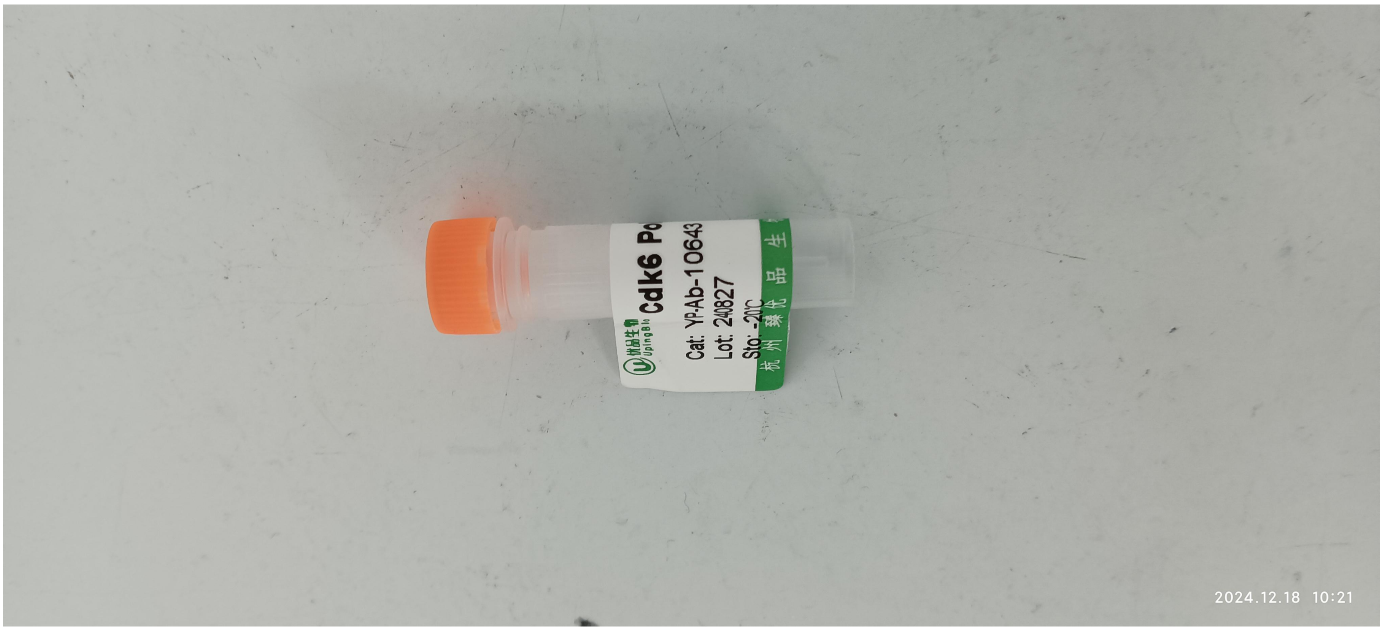
优品生物
UpingBio
Cdk6 Po
Cat: YP-Ab-10643
Lot: 240827
Sto: -20°C
杭州臻优品生
2024.12.18 10:21

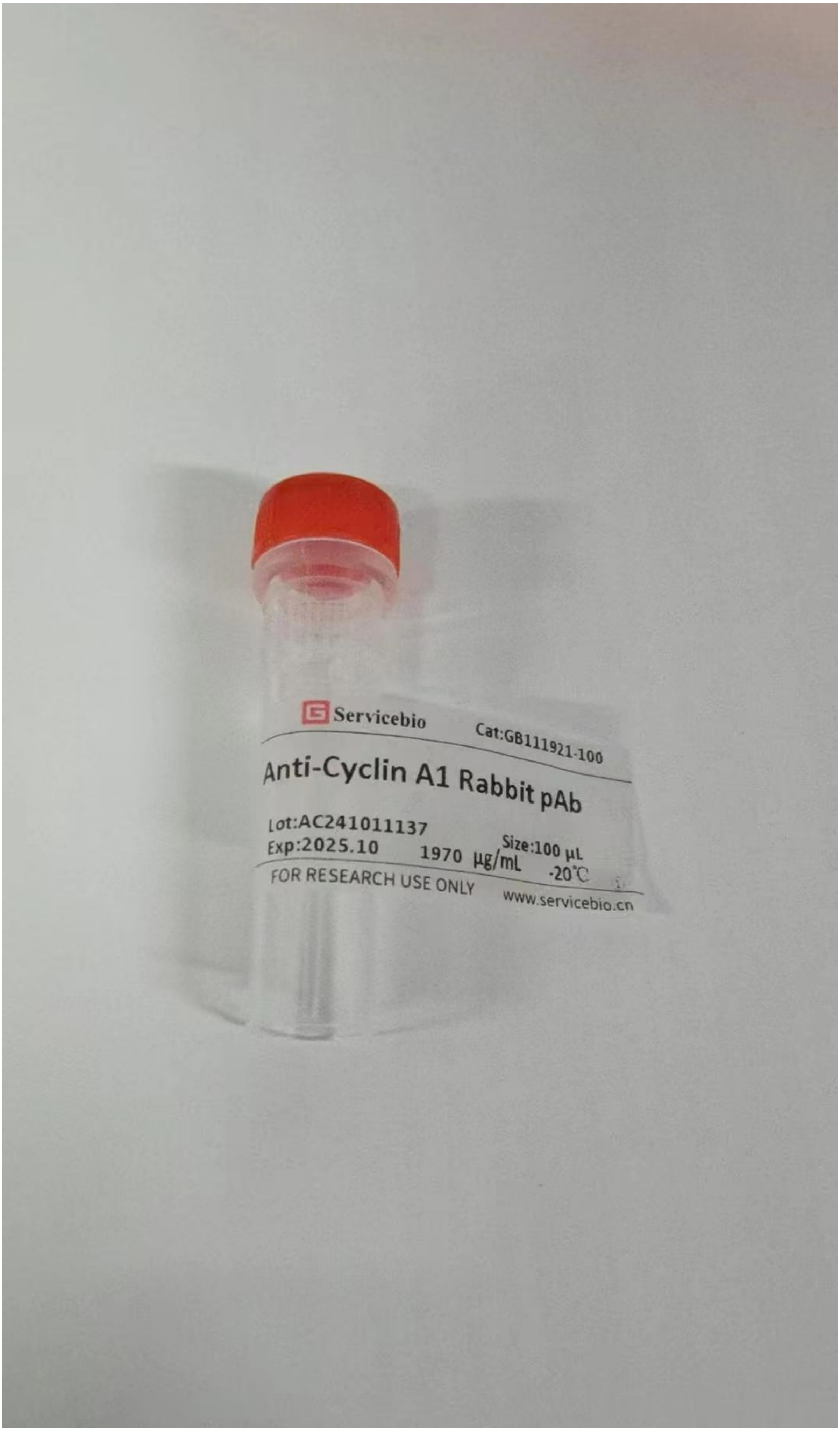
Servicebio
Cat:GB111921-100
Anti-Cyclin A1 Rabbit pAb
Lot:AC241011137
Exp:2025.10
Size:100 μL
1970 μg/mL
-20℃
FOR RESEARCH USE ONLY
www.servicebio.cn

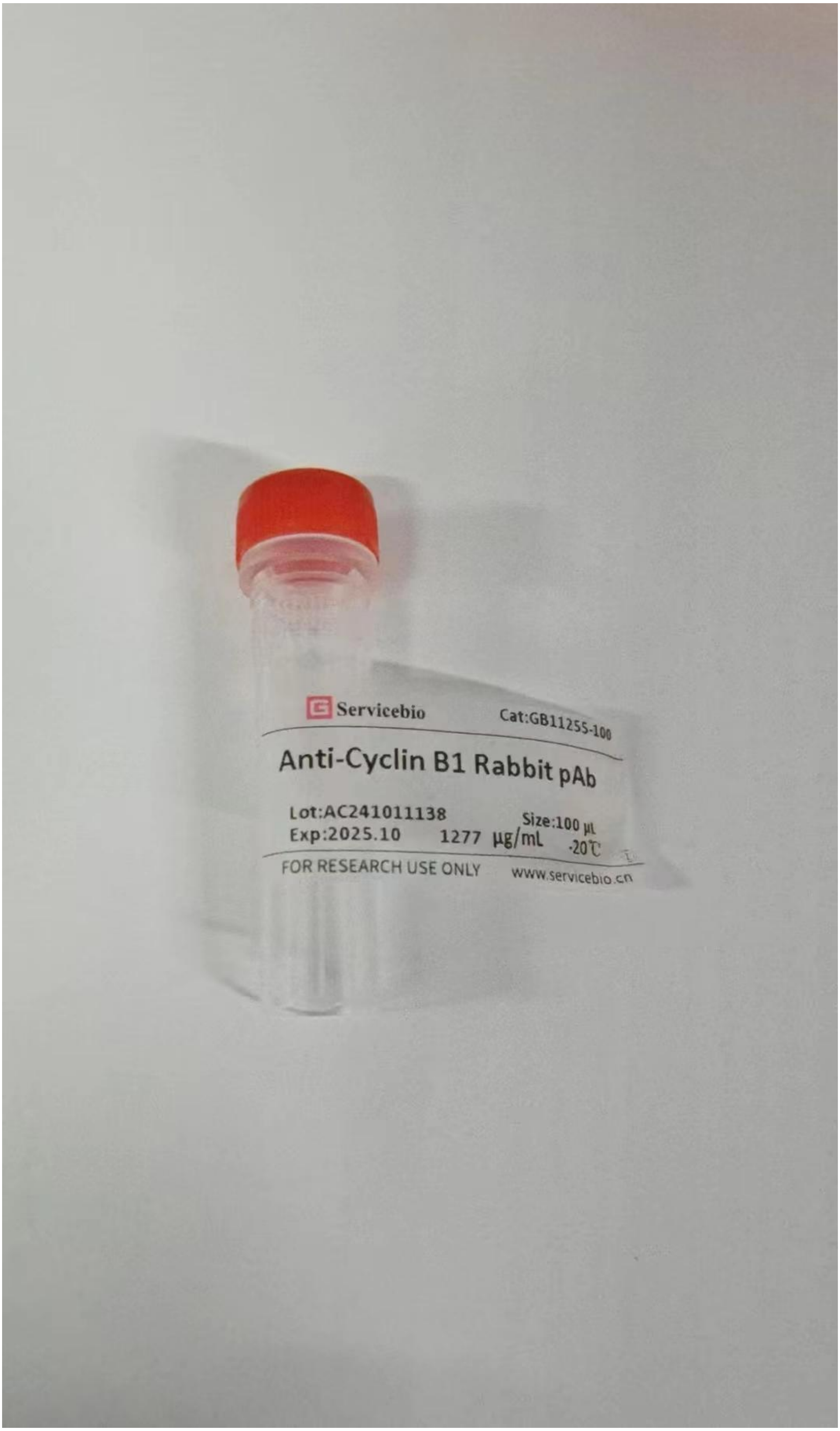
Servicebio
Cat:GB11255-100
Anti-Cyclin B1 Rabbit pAb
Lot:AC241011138
Size:100 μL
Exp:2025.10
1277 μg/mL
-20℃
FOR RESEARCH USE ONLY
www.servicebio.cn

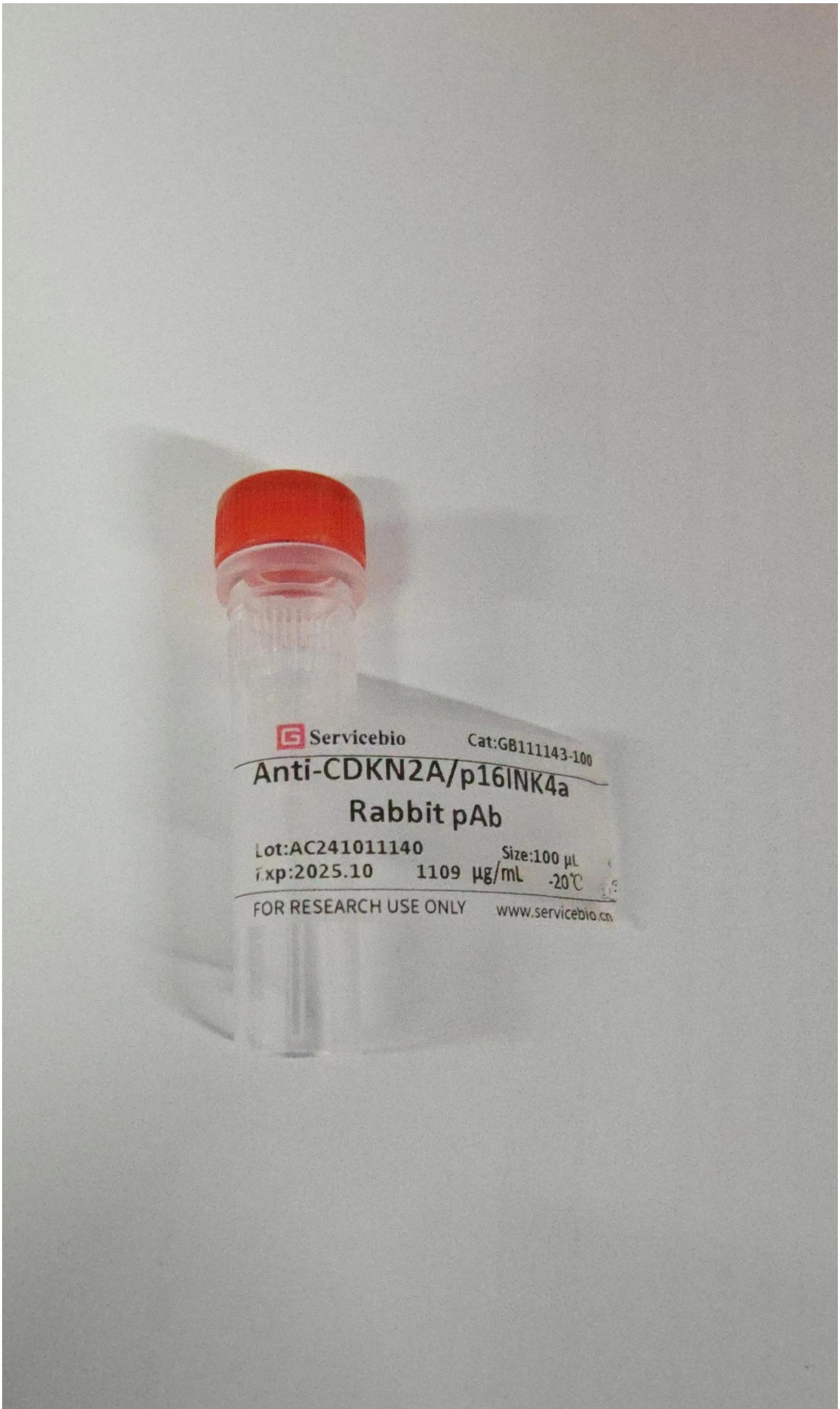

Servicebio
Cat:GB111143-100
Anti-CDKN2A/p16INK4a
Rabbit pAb
Lot:AC241011140
Size:100 μL
xp:2025.10
1109 μg/mL
-20℃
FOR RESEARCH USE ONLY
www.servicebio.cn

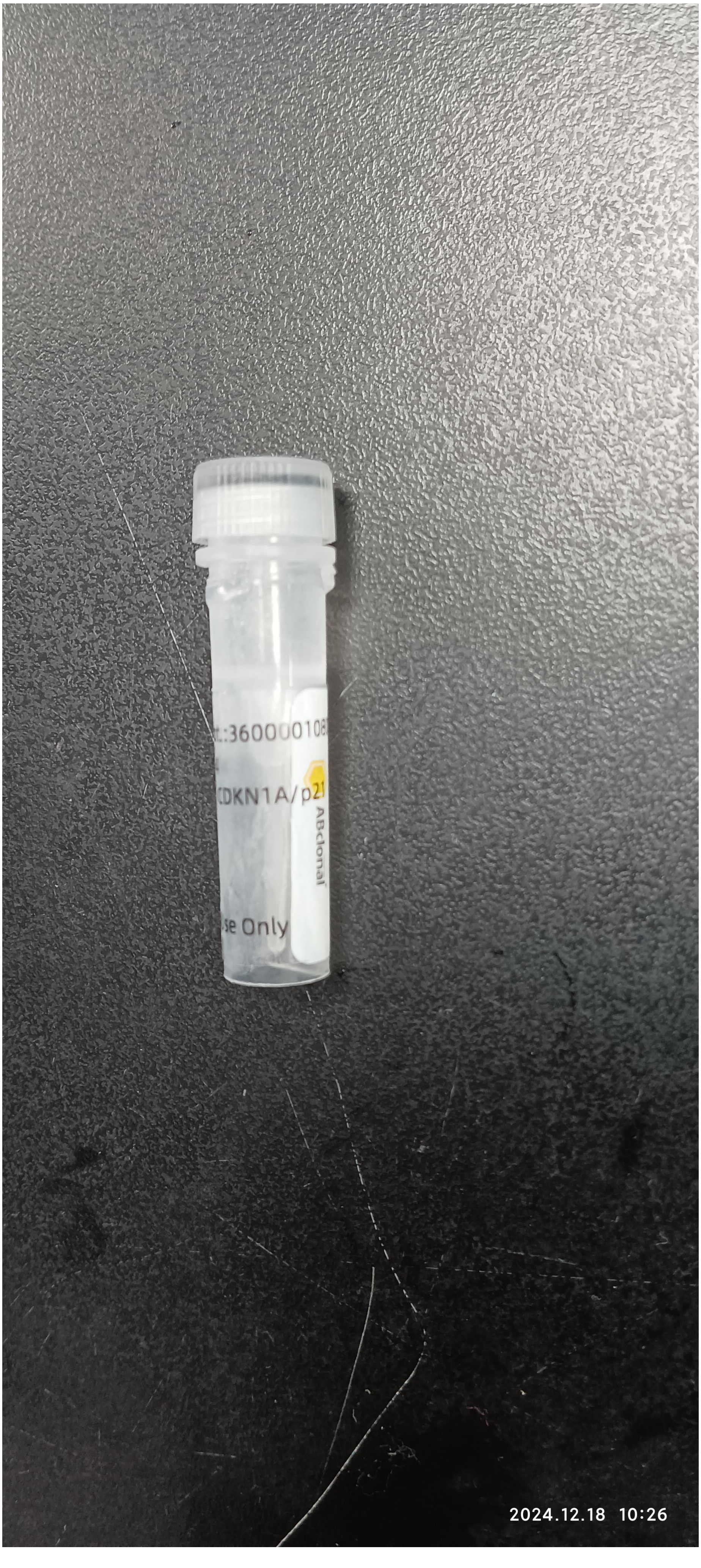

t:360000108
CDKN1A/p21
ABclonal
se Only
2024.12.18 10:26

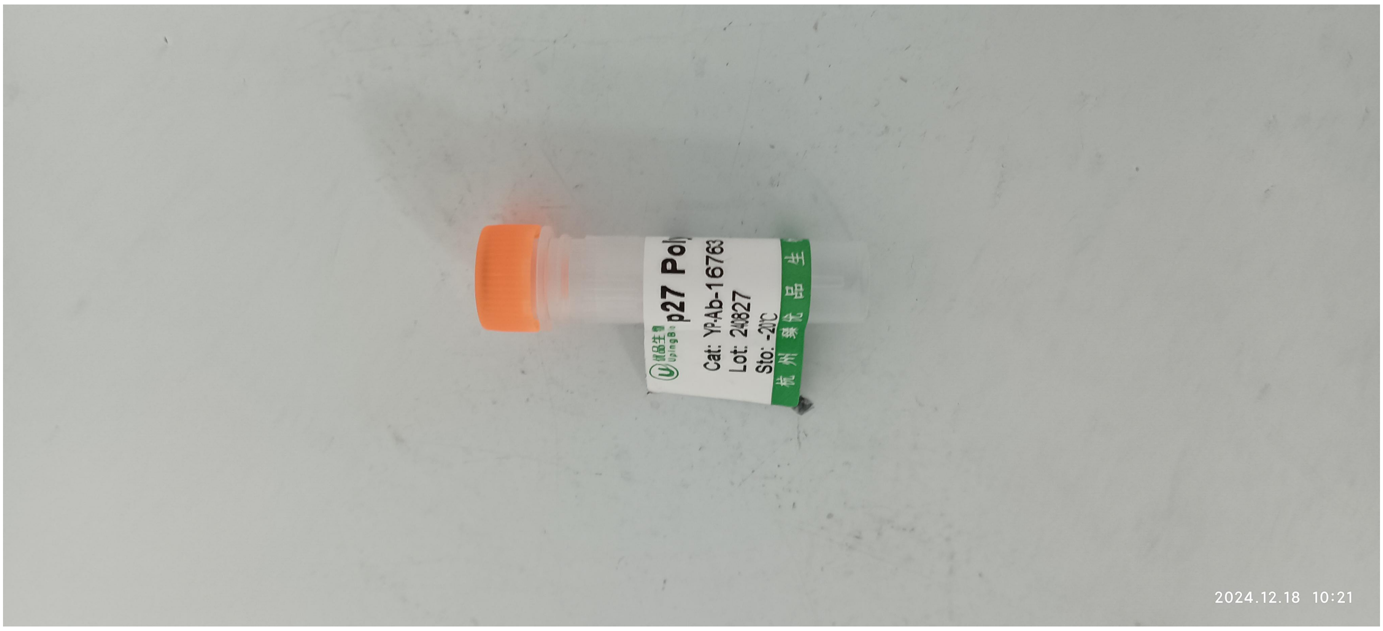
p27 Pol
Cat: YP-Ab-16763
Lot: 240827
Sto: -20℃
2024.12.18 10:21

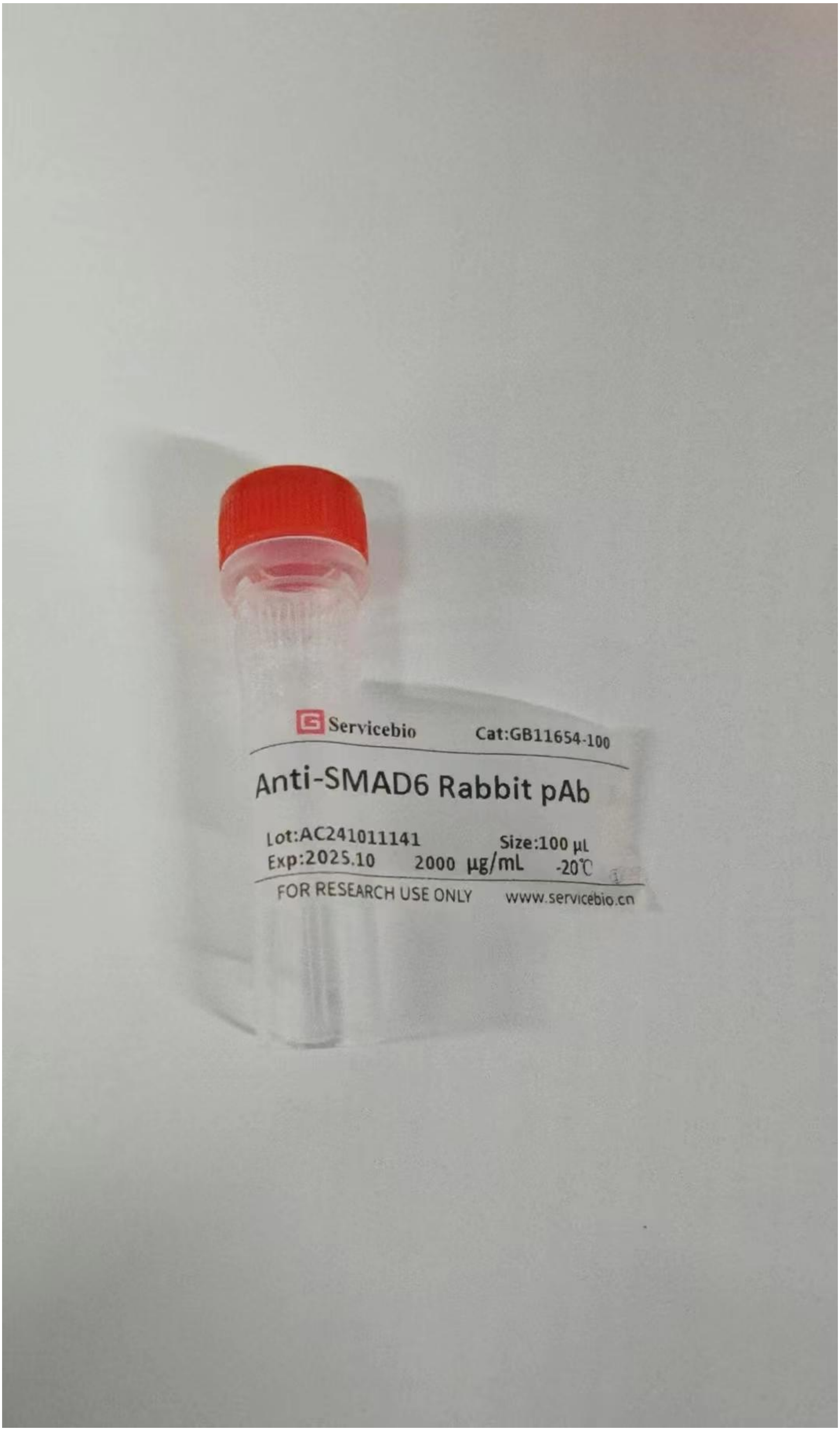
Servicebio
Cat:GB11654-100
Anti-SMAD6 Rabbit pAb
Lot:AC241011141
Size:100 µL
Exp:2025.10
2000 µg/mL
-20℃
FOR RESEARCH USE ONLY
www.servicebio.cn

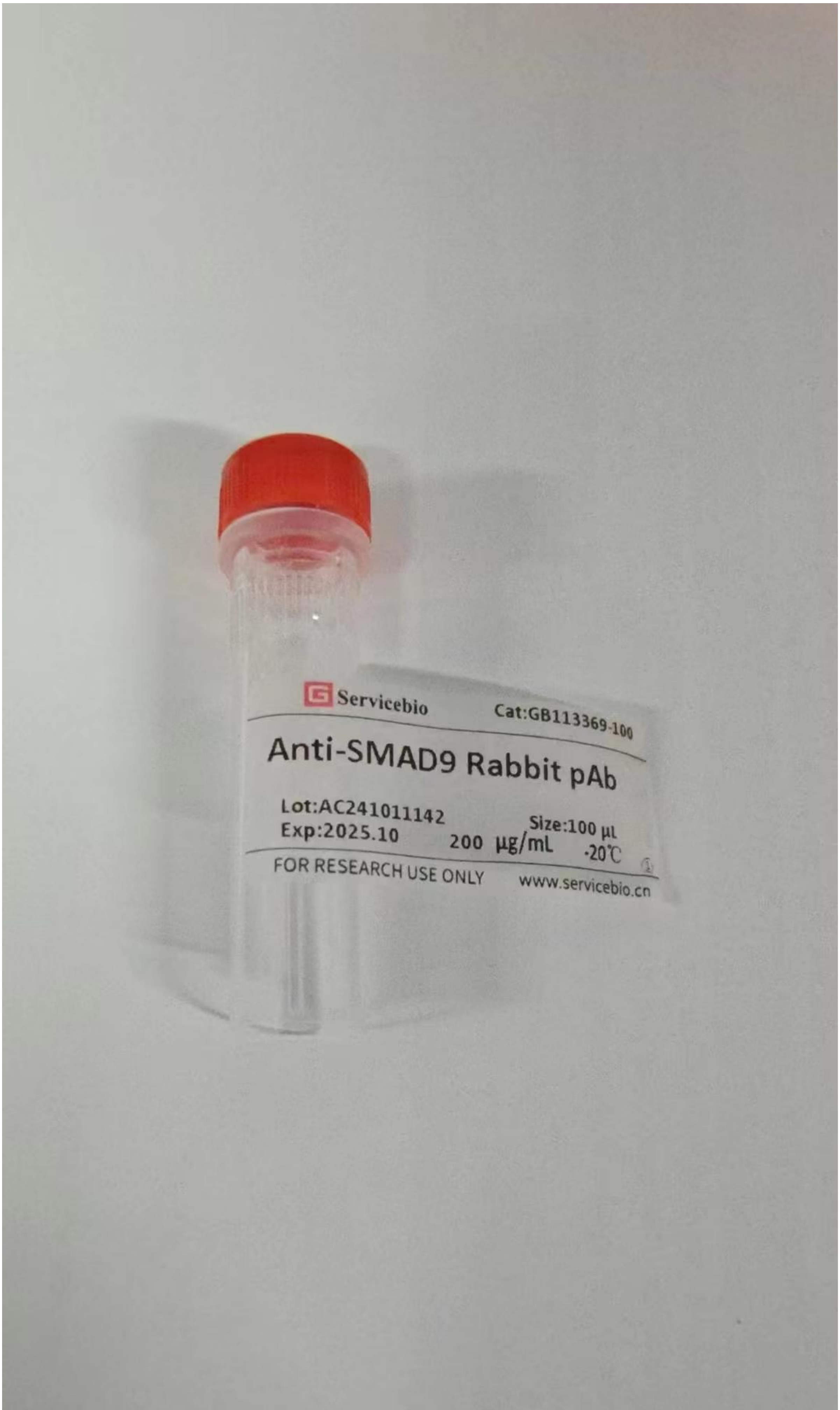
Servicebio
Cat:GB113369-100
Anti-SMAD9 Rabbit pAb
Lot:AC241011142
Exp:2025.10
Size:100 μL
200 μg/mL
-20℃
FOR RESEARCH USE ONLY
www.servicebio.cn

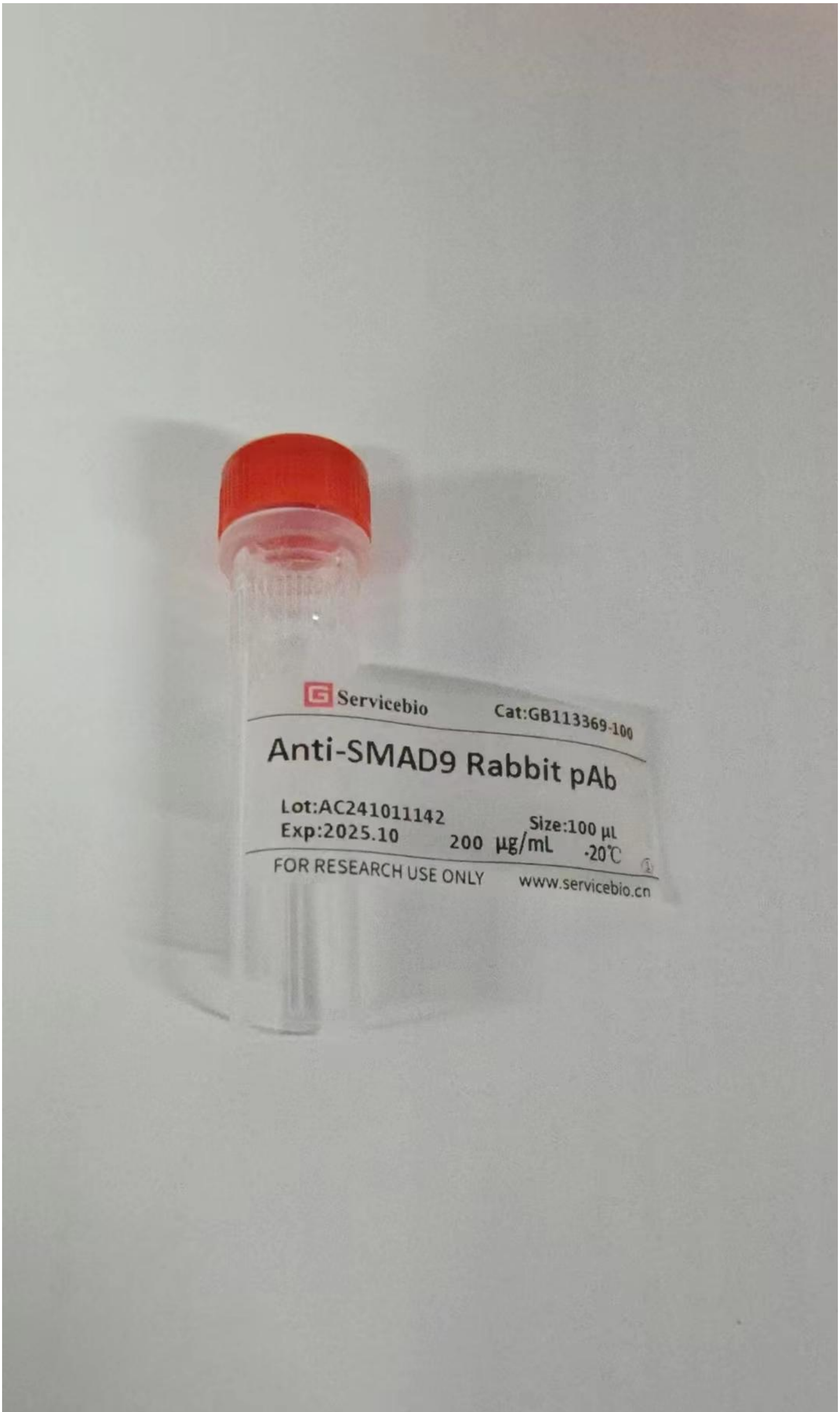
Servicebio
Cat:GB113369-100
Anti-SMAD9 Rabbit pAb
Lot:AC241011142
Exp:2025.10
Size:100 μL
200 μg/mL
-20℃
FOR RESEARCH USE ONLY
www.servicebio.cn
